# Tailored Transition‐Metal Coordination Environments in Imidazole‐Modified DNA G‐Quadruplexes

**DOI:** 10.1002/chem.201903445

**Published:** 2019-10-10

**Authors:** Philip M. Punt, Guido H. Clever

**Affiliations:** ^1^ Faculty of Chemistry and Chemical Biology TU Dortmund University Otto-Hahn-Straße 6 44227 Dortmund Germany

**Keywords:** bioinorganic chemistry, coordination chemistry, DNA, G-quadruplexes, imidazole

## Abstract

Two types of imidazole ligands were introduced both at the end of tetramolecular and into the loop region of unimolecular DNA G‐quadruplexes. The modified oligonucleotides were shown to complex a range of different transition‐metal cations including Ni^II^, Cu^II^, Zn^II^ and Co^II^, as indicated by UV/Vis absorption spectroscopy and ion mobility mass spectrometry. Molecular dynamics simulations were performed to obtain structural insight into the investigated systems. Variation of ligand number and position in the loop region of unimolecular sequences derived from the human telomer region (htel) allows for a controlled design of distinct coordination environments with fine‐tuned metal affinities. It is shown that Cu^II^, which is typically square‐planar coordinated, has a higher affinity for systems offering four ligands, whereas Ni^II^ prefers G‐quadruplexes with six ligands. Likewise, the positioning of ligands in a square‐planar versus tetrahedral fashion affects binding affinities of Cu^II^ and Zn^II^ cations, respectively. Gaining control over ligand arrangement patterns will spur the rational development of transition‐metal‐modified DNAzymes. Furthermore, this method is suited to combine different types of ligands, for example, those typically found in metalloenzymes, inside a single DNA architecture.

## Graphical abstract

**Unimolecular DNA G‐quadruplexes**, modified with metallo‐enzyme‐inspired imidazole moieties, can be fine‐tuned to bind a variety of transition‐metal cations in their loop region.

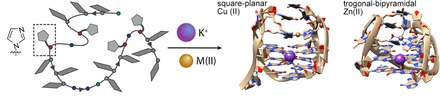

## Introduction

In the biological world, transition metals are key players in countless central processes ranging from structural stabilization over electron transport and oxygen metabolism to enzyme catalysis.^1^ Which metal suits which function largely depends on its redox properties, accessible spin states and Lewis acidity/basicity. Embedded in a protein environment, these properties are fine‐tuned by structurally defined and highly conserved coordination spheres, typically consisting of proteinogenic amino acid side chains and backbones.^2^

The first shells of biological coordination sites usually share some of the following principles: 1) controlled number of donors 2) their precise, 3‐dimensional positioning around the metal cation, 3) heteroleptic donor combinations, and 4) mixtures of weaker and stronger coordinating ligands.^3^ Often neglected, however, is a thorough discussion of the second coordination sphere, which is in fact no less important than the first shell. It is involved in shaping the geometry of the active site and fine‐tuning the electronic environment. It may further control substrate and product transport, selectivity, proton‐shuttling through channels, electron transport and many further processes.^3^, ^4^

In this respect, the synthetic branch of bioinorganic chemistry usually investigates the design and synthesis of chelating coordination environments, often podands or macrocycles, meant to resemble the first coordination sphere found within metalloproteins and other metal‐binding biopolymers. A classical approach to mimic such coordination environments is the tedious multistep synthesis of chelate ligands and corresponding model complexes. Improving or modifying the structure and function of these coordination compounds requires a laborious variation of donor site number, position and chemistry, if no modular approach can be chosen.^5^

A different strategy to tackle this issue is to follow closer the natural pattern and utilize peptides or proteins to design defined coordination environments. Therefore, natural protein scaffolds can be covalently or noncovalently modified with catalytically active metal complexes, even those that do not occur in nature. Alternatively, de novo designed folding motifs—typically alpha helices and beta barrels—are synthesized and connected to arrange ligating amino acid side chains in the desired way around a metal binding site. A related strategy builds on reducing a natural metalloprotein to its minimal functional unit by removing all parts not mandatory for its function.^6^

This concept cannot be applied only to proteins but also to other biopolymers including nucleic acids. Transition‐metals have been embedded into nucleic acid structures by the non‐/covalent attachment of chelate ligands. Examples include cerium complexes acting as sequence‐specific DNA cleavers, palladium complexes for the generation of singlet oxygen, Cu^II^‐complexes for the enantioselective catalysis of Friedel–Crafts reactions, Michael Additions, sulfoxidations or *syn* hydrations.^7^

In contrast to these embedding strategies, the field of metal‐mediated base pairing studies metal coordination as replacement of the canonical Watson–Crick hydrogen bonds.^8^ Previously, this concept was mostly applied in duplex DNA and only a limited number of articles reported about metal‐mediated base interaction in higher secondary structures.^9^ Examples include metal‐coordinating triplex DNA,9a i‐motifs9b and G‐quadruplex structures.9c The latter ones assemble from guanine‐rich sequences, in which four guanine residues form planar G‐tetrads through circular Hoogsteen base pairing. Under incorporation of central monovalent cations, typically Na^+^ or K^+^, the tetrads stack via π–π interactions to give G‐quadruplexes.^10^

Recently, our group was the first to report an example of a transition‐metal‐binding G‐quadruplex in which one of the G‐tetrads was replaced by four pyridine ligands, allowing for the complexation of Cu^II^ and Ni^II^ cations.9c This concept was employed to realize the Cu^II^‐triggered inhibition of thrombin via a specific quadruplex aptamer, a molecular EPR ruler (with two copper centers acting as spin labels) and a Cu^II^‐switchable peroxidase mimic consisting of a DNA G‐quadruplex adduct with hemin.^11^, 12a

Inspired by the natural amino acid histidine, we herein report the incorporation of a new imidazole‐based ligand into tetra and unimolecular G‐quadruplexes.^12^ Although the inclusion in tetramolecular G‐quadruplexes offer only limited control of ligand number (multiples of four) and arrangement (3′ and/or 5′ end),^11^, 12a, 13 we herein show that unimolecular G‐quadruplexes present a highly robust system to vary the position and number of incorporated ligands. We present how this approach allows us to fine‐tune the coordination environment of different transition‐metal cations with respect to their preferred coordination number and geometry. This further enables the design of systems with coordinatively unsaturated metal centers, being potentially attractive for applications in catalysis. Gaining high control over the construction of specific coordination environments by a straightforward DNA sequence design represents a fundamental prerequisite on the way to develop new DNA‐based metalloenzyme mimics.

## Results and Discussion

**Figure 1:**
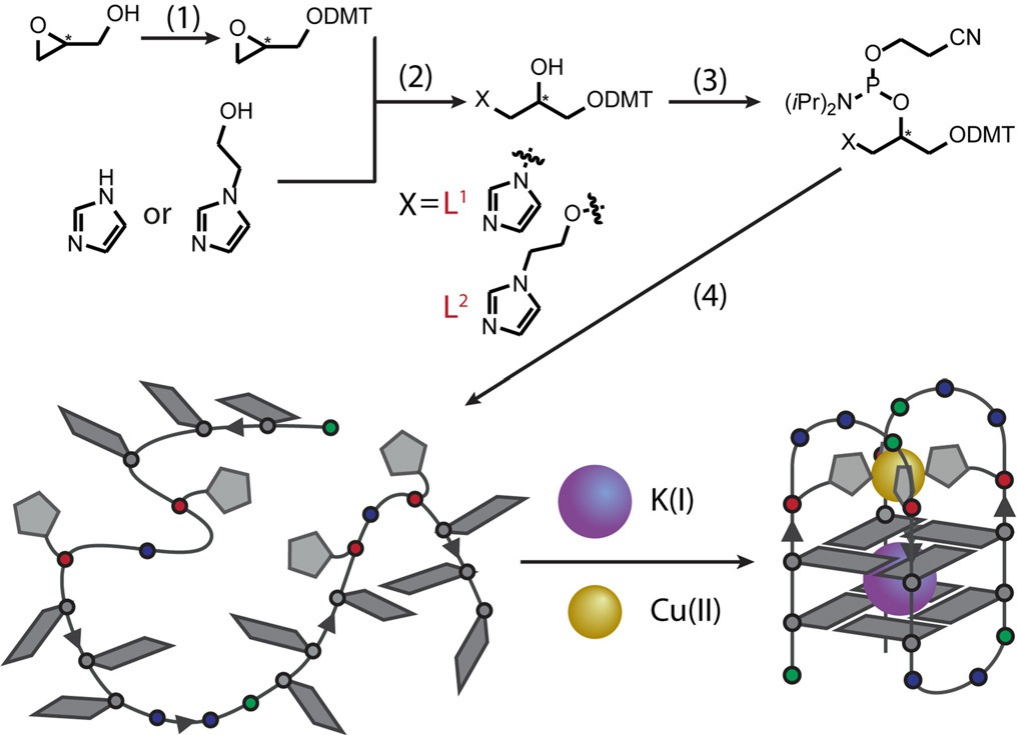
Synthesis of imidazole ligands **L^1^** and **L^2^**. (1) DMT‐Cl, glycidol and Et_3_N in CH_2_Cl_2_, (2) **L^1^**: imidazole and DMT‐glycidol in 1,4‐dioxane at 80 °C or **L^2^**: 1‐(2‐hydroxyethyl)imidazole, DMT‐glycidol and NaH in DMF at 40 °C, (3) CEDIP‐Cl, imidazole derivative and DIPEA in CH_2_Cl_2_ at rt, (4) DNA solid phase synthesis. DIPEA=*N*,*N*‐diisopropylethylamine; CEDIP‐Cl=2‐cyanoethyl *N*,*N*‐diisopropylchlorophosphoramidite.

For our study, the previously reported12a imidazole‐based ligand **L^1^** and its derivative **L^2^**, which bears an additional ethanol linker, were incorporated in both of their enantiomeric forms (**L^1*R/S*^**, **L^2*R/S*^**) into a number of tetramolecular and unimolecular G‐quadruplex sequences. The phosphoramidites needed for solid‐phase synthesis were accessed according to procedures reported before.12a An initial nucleophilic ring opening of DMT‐protected (*R*/*S*)‐glycidol (DMT = 4,4′‐dimethoxytrityl) was followed by a phosphorylation reaction to access phosphoramidite building blocks. Both stereoisomers with either *R*‐ or *S*‐configuration at the carbon in the branching position were prepared (Figure 1). DNA solid‐phase synthesis was performed according to standard protocols (Supporting Information). Coupling times for **L^1^** and **L^2^** were extended to maximize coupling efficiencies which were usually >99 % per coupling. After solid‐phase synthesis, DNA samples were deprotected in aqueous ammonia at 55 °C and purified in “DMT‐on” mode by using reversed‐phase HPLC. After purification, the final DMT‐group was removed by using C18‐Sepak cartridges and aqueous TFA (2 %).

G‐quadruplex formation was characterized by UV/Vis‐based melting experiments, thermal difference spectra (TDS), CD spectroscopy and mass spectrometry. In the case of melting experiments, a temperature‐dependent change of absorption at 295 nm was observed, which is characteristic for G‐quadruplex denaturation/renaturation.

**Figure 2:**
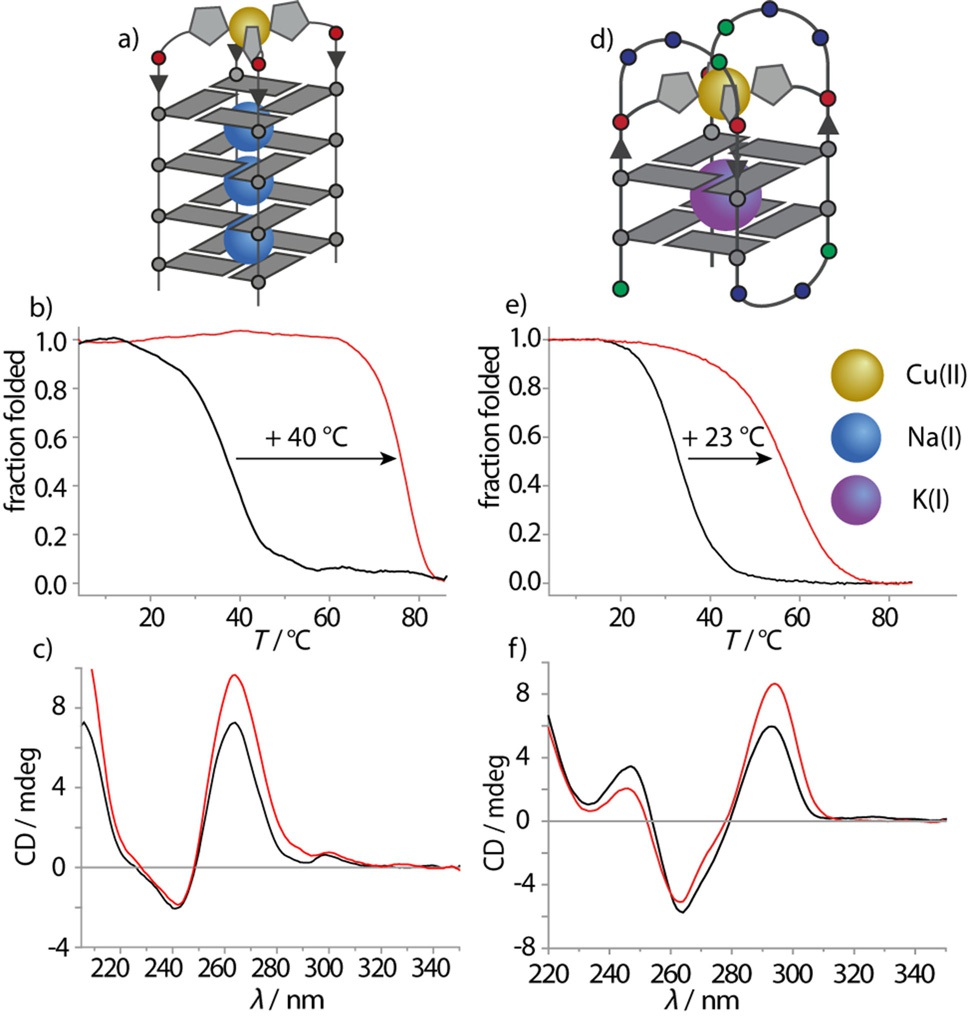
a) Schematic representation of G_4_ **L** in complex with Cu^II^, b) thermal denaturation spectra of G_4_ **L^2*S*^** before (black) and after (red) Cu^II^ addition, c) CD spectra of G_4_ **L^2*S*^** before (black) and after (red) Cu^II^ addition, d) schematic representation of htel**L**_4_, e) thermal denaturation spectra of htel**L^2*S*^**_4_ before (black) and after (red) Cu^II^ addition, f) CD spectra of htel**L^2*S*^**_4_ before (black) and after (red) Cu^II^ addition. Samples were prepared in 100 mm NaCl (tetramolecular) or KCl (unimolecular), 10 mm LiCaCo pH 7.2 and 0.94 or 1.88 μm Cu^II^ (tetramolecular and unimolecular, respectively) at G‐quadruplex concentrations of 3.75 μm (tetramolecular) and 1.875 μm (unimolecular).

Incorporation of **L^1^** or **L^2^** at the 5′ terminal position of tetramolecular G‐quadruplex G_4_ **L** resulted in the formation of a parallel strand arrangement as indicated by CD spectroscopy, showing a characteristic positive Cotton effect at approximately 264 nm (Figure 2). An additional maximum could be observed at 295 nm, the origin of which is still under debate.^13^ Thermal denaturation experiments revealed a slightly higher stability for G_4_ **L^2^** [*T*_m_ (G_4_ **L^2*R*^**)=34 °C, *T*_m_ (G_4_ **L^2*S*^**)=36 °C; *T*_m_=melting temperature] compared to G_4_ **L^1^** (*T*_m_(G_4_ **L^1*R*^**)=32 °C, *T*_m_(G_4_ **L^1*S*^**)=31 °C]. In line with our previous observations, this effect could be explained by the longer linker of **L^2^** that facilitates better π‐stacking of the imidazole moieties with the upper G‐tetrad, contributing to a higher thermal stability.^13^ Subsequently, transition‐metal binding was investigated. In accordance with our previously reported results for **L^1^**, the **L^2^**‐modified G‐quadruplexes G_4_ **L^2*R*^** and G_4_ **L^2*S*^** were observed to complex Cu^II^, Ni^II^, Zn^II^ and Co^II[14]^ cations as indicated by strong thermal stabilization effects (Table 1; Supporting Information, Table S3) by retaining the all‐parallel topology. All sequences were found to bind one equivalent of the respective metal cation, whereas the addition of further amounts resulted in no significant extra stabilization, thus indicating specific binding to the imidazole‐lined cavity in a 1:1 fashion. Interestingly, G_4_ **L^2^** shows a higher thermal stability compared to G_4_ **L^1^** in absence of transition metals. After addition of Cu^II^, however, G_4_ **L^1^** [Δ*T*_m_ (G_4_ **L^1*S*^**)=+48 °C, Δ*T*_m_ (G_4_ **L^1*R*^**)=+51 °C] was significantly more stabilized compared to G_4_ **L^2^** [Δ*T*_m_ (G_4_ **L^2*R*^**)=+40 °C, Δ*T*_m_ (G_4_ **L^2*S*^**)=+40 °C]. This effect can be explained by stronger π–π interactions between **L^2^** and the neighboring G‐tetrad that need to break up to facilitate metal coordination and, thus, reduce the metal‐mediated thermal stabilization.^13^ In addition, although for **L^1^** the stereochemical configuration showed an influence on the thermal stabilization, in case of **L^2^** no influence of the stereo‐configuration was observed.

**Table 1:** Melting temperatures *T*_m_ (and Δ*T*_m_) of unimolecular and tetramolecular G‐quadruplexes, respectively, in absence and presence of different transition metal cations. Conditions: 3.75 (tetramolecular) and 1.88 μm (unimolecular) ssDNA in 100 mm NaCl (tetramolecular) or KCl (unimolecular), unless stated differently; 10 mm LiCaCo pH 7.2 and, if present, 1 equiv transition‐metal cations (with respect to the folded G‐quadruplex). “No metal” refers to the absence of transition‐metal cations.

	No metal	Cu^II^	Ni^II^	Zn^II^	Co^II^
G_4_ **L^1*S*^**	31	79 (+48)	77 (+46)	54 (+23)	64 (+33)
G_4_ **L^2*S*^**	36	76 (+40)	73 (+37)	52 (+16)	63 (+27)
htel**L^1*S*^**_**4**_	37	36 (−1)	36 (−1)	36 (−1)	37 (+0)
htel**L^2*S*^**_**2**_B^[a]^	53	55 (+2)	53 (+0)	54 (+1)	53 (+0)
htel**L^2*S*^**_**3**_B^[a]^	50	56 (+6)	51 (+1)	52 (+2)	50 (+0)
htel**L^2*S*^**_**4**_	33	56 (+23)	45 (+12)	36 (+3)	35 (+2)
htel**L^2*S*^**_**5**_	33	54 (+21)	55 (+22)	37 (+4)	37 (+4)
htel**L^2*S*^**_**6**_	36	54 (+18)	59 (+23)	44 (+8)	44 (+8)
htel**L^2*S*^**_**7**_	28	43 (+15)	46 (+18)	36 (+8)	36 (+8)

[a] Measured in 100 mm NaCl.

To further support the formation of the proposed G‐quadruplex‐metal complexes, native ESI‐MS experiments coupled to trapped ion mobility spectrometry (TIMS) were performed. To differentiate between folded and unfolded G‐quadruplexes in the gas phase, two phenomena are most instructive. First, if tetramolecular G‐quadruplexes are investigated and the structure is denatured, single‐stranded DNA instead of a tetramer would be observed and second, valid for tetra‐ and unimolecular G‐quadruplexes, ESI mass spectrometry from electrolyte‐containing solutions always gives rise to series of unspecific adducts with sodium or potassium cations. For fully denatured species, a statistical distribution of adducts starting with zero cations would be observed and for a native, folded species a distribution is observed starting with *n*−1 explicitly bound cations, where *n* is the number of G‐tetrads.^15^

For G_4_ **L^2*R*^**, the analysis gave the following picture: although the quadruplex was found to be unstable in absence of Cu^II^ cations, hence denatured to single strands in the gas phase, the addition of one equiv of Cu^II^ led to the observation of [G_4_ **L^2*R*^**K_3_Cu]^4−^ as main species, besides a series of additional unspecific potassium adducts. Corresponding values for the TIMS‐derived collisional cross‐sections (CCS) (G_4_ **L^2*R*^**=814 Å^2^, G_4_ **L^2*S*^**=814 Å^2^) were comparable to reported values for unmodified G‐quadruplexes.^16^ Compared to the reported CCS for G_4_ **L^1*R*^** (793 Å^2^), this corresponded to an increase of 21 Å^2^, which was ascribed to the longer linker of **L^2^**.^13^

**Figure 3:**
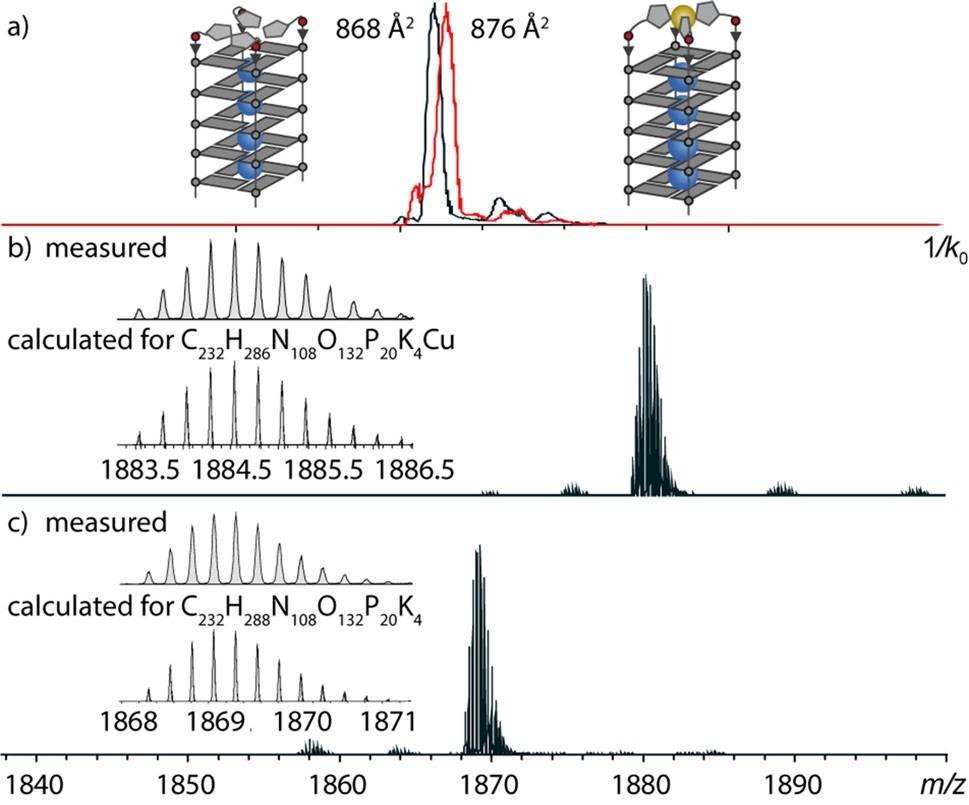
a) Native ESI‐MS and trapped ion‐mobility time‐of‐flight (timsTOF) experiments of G_5_ **L^2*R*^** in absence and in complex with Cu^II^. (a) Ion mobilities and corresponding collisional cross‐sections. Ion‐mobility‐extracted mass spectra of G_5_ **L^2*R*^** b) in complex with Cu^II^ and c) in absence of Cu^II^.

Considering that we were interested in comparing CCS values of folded G‐quadruplexes in the presence and absence of Cu^II^, the more stable sequence G_5_ **L^2*R*^** consisting of one additional G‐tetrad was investigated. Indeed, G_5_ **L^2*R*^** was stable enough in absence of Cu^II^ and the intact G‐quadruplex was observed with a CCS of 868 Å^2^ in the ESI TIMS experiment. Addition of Cu^II^ resulted in a slightly larger CCS of 876 Å^2^ (Figure 3), which was contradicting the assumption that Cu^II^ coordination would “catch“ freely dangling imidazole ligands resulting in a smaller CCS. However, gas‐phase molecular dynamics (MD) simulations suggest that in absence of Cu^II^, the imidazole ligands lay flat on top of the G‐quadruplex or in the groove regions and in presence of Cu^II^ they rearrange in a more spacious propeller shape resulting in a slight increase in size (Supporting Information, Figures S85 and S86).

With promising results obtained for tetramolecular G‐quadruplexes, we turned towards unimolecular G‐quadruplexes. Therefore, in the human telomeric G‐quadruplex sequence htel, one G‐tetrad was replaced with four imidazole ligands, now called htel**L**_4_. Investigation by CD spectroscopy, by using KCl as the electrolyte, indicated an antiparallel topology for the modification with both ligand derivatives (**L^1*R/S*^** and **L^2*R/S*^**) with a positive Cotton effect at around 295 nm (Figure 2). Thermal denaturation studies of the modified G‐quadruplexes showed significantly lower melting temperatures [*T*_m_ (htel**L^1*S*^**_4_)=37 °C, *T*_m_ (htel**L^2*S*^**_4_)=33 °C] compared to unmodified htel (*T*_m_=64 °C) due to the lack of one G‐tetrad.^11^ Addition of different transition metals to htel**L^1*R/S*^**_*4*_ showed no signs of metal complexation, neither by CD spectroscopy nor by thermal denaturation studies. An explanation was found in the very short linker of **L^1^**, which prevents the ligand from reaching the metal center from all four sides simultaneously and, thus, disabling metal complexation in the unimolecular system. This hypothesis was confirmed after investigating htel**L^2*R/S*^**_**4**_, which features an ethoxy‐elongated linker, which was now able to complex Cu^II^, Ni^II^, Zn^II^ and Co^II^. Although the addition of 1 equiv Cu^II^ (Δ*T*_m_=+23 °C) and Ni^II^ (Δ*T*_m_=+12 °C) to htel**L^2*S*^**_4_ was accompanied by a strong thermal stabilization, addition of Co^II^ (Δ*T*_m_=+2 °C) and Zn^II^ (Δ*T*_m_=+3 °C) resulted only in weak stabilization effects. Addition of further amounts of the respective transition metals showed no significant further increase but rather a decrease of the thermal stability, which is consistent with the specific binding of one metal cation to the imidazole modifications. Interestingly, the extent of metal‐mediated thermal stabilization was highly dependent on the stereo configuration of **L^2^**. Thus, for htel**L^2*S*^**_4_ higher thermal stabilizations were generally observed compared to those of its diastereomer htel**L^2*R*^**_4_.

To further analyze the G‐quadruplex metal complexes, mass spectrometric investigations of htel**L^2*R*^**_4_ in the absence and presence of Cu^II^, Ni^II^, Zn^II^ and Co^II^ were performed. In the absence of transition metals, one main signal corresponding to the single strand was found, followed by unspecific potassium adducts, which is consistent with a denatured G‐quadruplex. However, after addition of Cu^II^ and Ni^II^, new main species corresponding to [htel**L^2*R*^**_4_KCu]^4−^ and [htel**L^2*R*^**_4_KNi]^4−^, respectively, were observed, consistent with natively folded G‐quadruplexes. In the case of Zn^II^ and Co^II^, the results were less clear showing a mixture of signals corresponding to denatured single strand and folded [htel**L^2*R*^**_4_KZn]^4−^ or [htel**L^2*R*^**_4_KCo]^4−^, respectively. This was attributed to the lower thermal stability of the Zn^II^ and Co^II^ complexes, as compared to Cu^II^ and Ni^II^.

Knowing that only **L^2^** was able to complex transition‐metal cations in unimolecular systems, the next question was whether an increase or decrease in the number of incorporated ligands would affect G‐quadruplex topology and metal coordination. Therefore, sequences htel**L^2*R/S*^**_2‐7_ with two‐to‐seven ligands were synthesized and investigated with respect to their folding topology and thermal stability. In htel**L^2*R/S*^**_2_ and htel**L^2*R/S*^**_3_, two or one ligands present in the parental sequence were replaced with thymine bases. TDS spectra revealed that both sequences htel**L^2*R/S*^**_2_ and htel**L^2*R/S*^**_3_ formed G‐quadruplexes, but CD spectroscopy indicated a loss of the clear antiparallel conformation, presumably leading to mixtures of different topologies (Supporting Information, Figure S47). This prompted the design of two new sequences htel**L^2*R/S*^**_2_B and htel**L^2*R/S*^**_3_B. In contrast to the previous sequences where the ligands were incorporated by replacing one of the G‐tetrads, now all three G‐tetrads were retained and instead thymine and adenine in the loop regions were replaced with **L^2^**. Now, in NaCl a mainly antiparallel topology was observed for both sequences. In KCl, however, a signature was observed that either represents a hybrid 3+1 or a mix of topologies. Addition of Cu^II^ cations induced transformation into a pronounced antiparallel topology (Supporting Information, Figures S55 and S57). This sodium/potassium‐dependent topology change was similarly reported for the unmodified htel sequence.^17^ Considering that only in the antiparallel topology all ligands are arranged correctly (in opposite loops on one face of the G‐quadruplex stack), subsequent studies with htel**L^2*R/S*^**_2_B and htel**L^2*R/S*^**_3_B were performed in sodium‐containing buffers. Due to the additional G‐tetrad for both sequences, high thermal stabilities with *T*_m_=53 °C (htel**L^2*S*^**_2_B) were observed. The addition of Cu^II^ cations to htel**L^2*S*^**_2_B gave a weak thermal stabilization of Δ*T*_m_=+2 °C while for htel**L^2*S*^**_3_B addition of Cu^II^ led to Δ*T*_m_=+6 °C and Zn^II^ Δ*T*_m_=+2 °C. Furthermore, Cu^II^ addition induced a change of the CD signature to a more pronounced antiparallel topology, thus indicating the existence of a mixture of different topologies prior to copper complexation.^11^

Next, we increased the number of contained imidazole moieties to five, six and even seven by replacing adenines in the loop regions with **L^2^** in case of htel**L^2*R/S*^**_5_ and htel**L^2*R/S*^**_6_. For htel**L^2*R/S*^**_7_, an additional ligand was introduced into the loop, thus extending the overall sequence to 23 bases. TDS and CD spectroscopy supported the formation of G‐quadruplexes in an antiparallel topology for all sequences, likewise to htel**L^2*R/S*^**_4_. Addition of Zn^II^ and Co^II^ to htel**L^2*S*^**_5_ showed weak thermal stabilizations [Δ*T*_m_ (Zn^II^)=+4 °C, Δ*T*_m_ (Co^II^)=+4 °C] comparable to those observed for htel**L^2*S*^**_4_. More exciting were the observations made after addition of Ni^II^ and Cu^II^. Although in the case of Cu^II^ (Δ*T*_m_=+21 °C) the stabilization of htel**L^2*S*^**_5_ was lower compared to that of htel**L^2*S*^**_4_ (Δ*T*_m_=+23 °C), the addition of Ni^II^ resulted in the exact opposite effect, showing a higher stabilization for htel**L^2*S*^**_5_ (Δ*T*_m_=+22 °C) compared to htel**L^2*S*^**_4_ (Δ*T*_m_=+12 °C).

**Figure 4:**
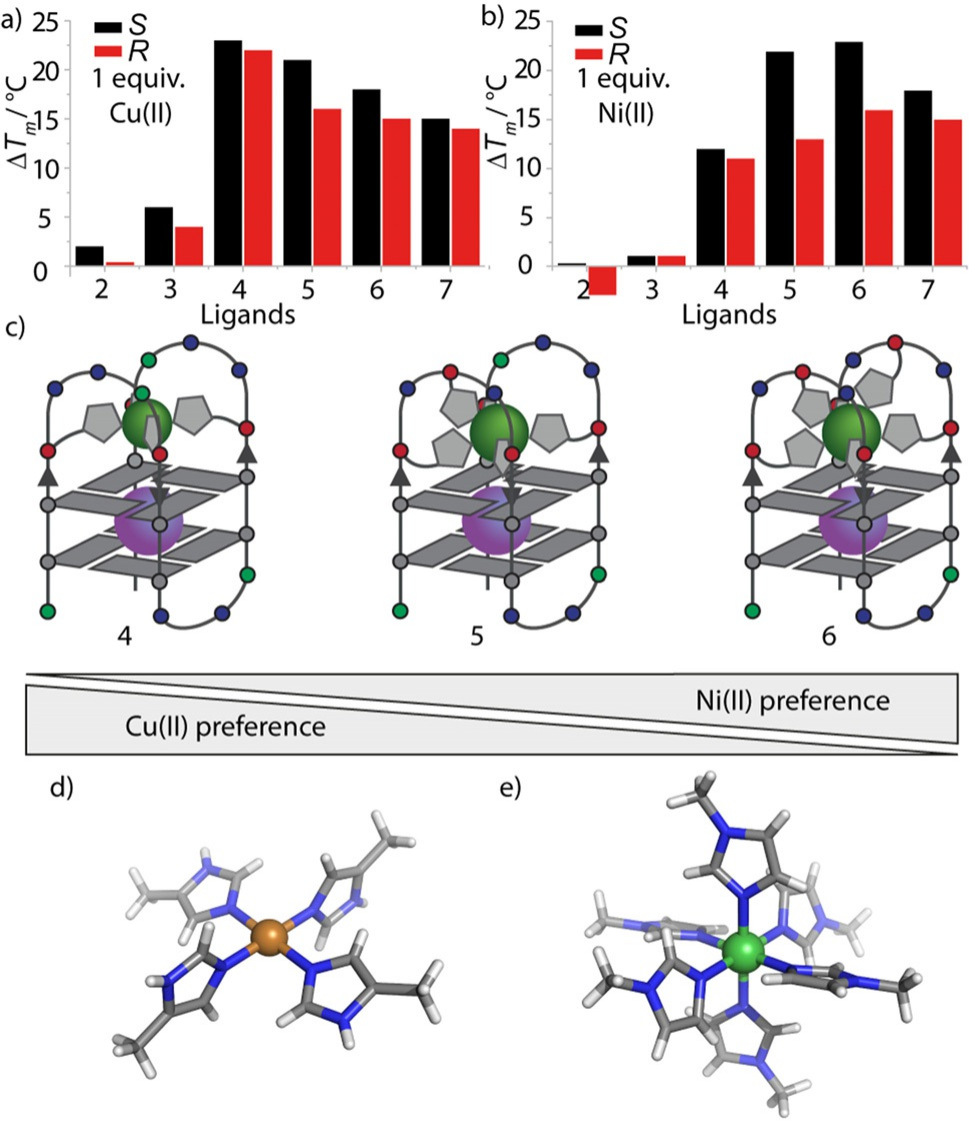
Fine‐tuning of metal affinities. The bar diagrams in a) and b) display the changing G‐quadruplex stabilizations after addition of Cu^II^ and Ni^II^ to htel**L^2R/*S*^**_2‐3_B and htel**L^2R/*S*^**_4‐7_. In a), decreasing Cu^II^‐mediated stabilization with an increase in ligand number from four to six is observed, whereas in b), the opposite effect is observed, that is, an increased stabilization from four to six ligands. c) Schematic illustration of the fine‐tuning of Cu^II^ and Ni^II^ affinities. In d) and e), reported crystal structures of [Cu^II^(4‐methyl‐imidazole)_4_] and [Ni^II^(*N*‐methyl‐imidazole)_6_] complexes are shown.^18^

This trend further continued with htel**L^2*S*^**_6_ showing an even lower thermal stabilization (Δ*T*_m_=+18 °C) after Cu^II^ addition compared to Ni^II^ (Δ*T*_m_=+23 °C). To explain these findings, a look at the preferred coordination environments of Cu^II^ and Ni^II^ is instructive. Cu^II^ is typically square‐planar coordinated (with or without loosely bound water molecules in axial positions) as can be observed in reported crystal structures of Cu^II^ imidazole complexes (Figure 4 d).^18^ Ni^II^ exhibits a more versatile coordination chemistry with coordination numbers usually ranging from four to six. In the case of Ni^II^‐imidazole complexes, crystal structures are known in which six imidazole ligands are coordinated to the Ni^II^ cation in an octahedral fashion (Figure 4 e).^18^ The herein presented results nicely reflect the preferred coordination numbers of Cu^II^, which is satisfied with four ligands, and Ni^II^, which tends to be coordinated by six imidazole ligands. Hence, our system seems to allow for a selective fine‐tuning of metal affinities by varying the number of introduced ligands (although it has to be mentioned that metal‐mediated thermal stabilizations might not necessarily directly correspond to the underlying complexation constants). As expected, this trend abruptly stopped after incorporation of ligand number seven: Whereas Ni^II^ addition to htel**L^2*S*^**_7_ (Δ*T*_m_=+18 °C) still caused a higher thermal stabilization compared to Cu^II^ (Δ*T*_m_=+15 °C), a drop of the thermal stabilization compared to htel**L^2*S*^**_6_ was observed (see Figure 4). This could be a result of an overcrowded system in which more ligands are offered to Ni^II^ than it can coordinate.

**Figure 5:**
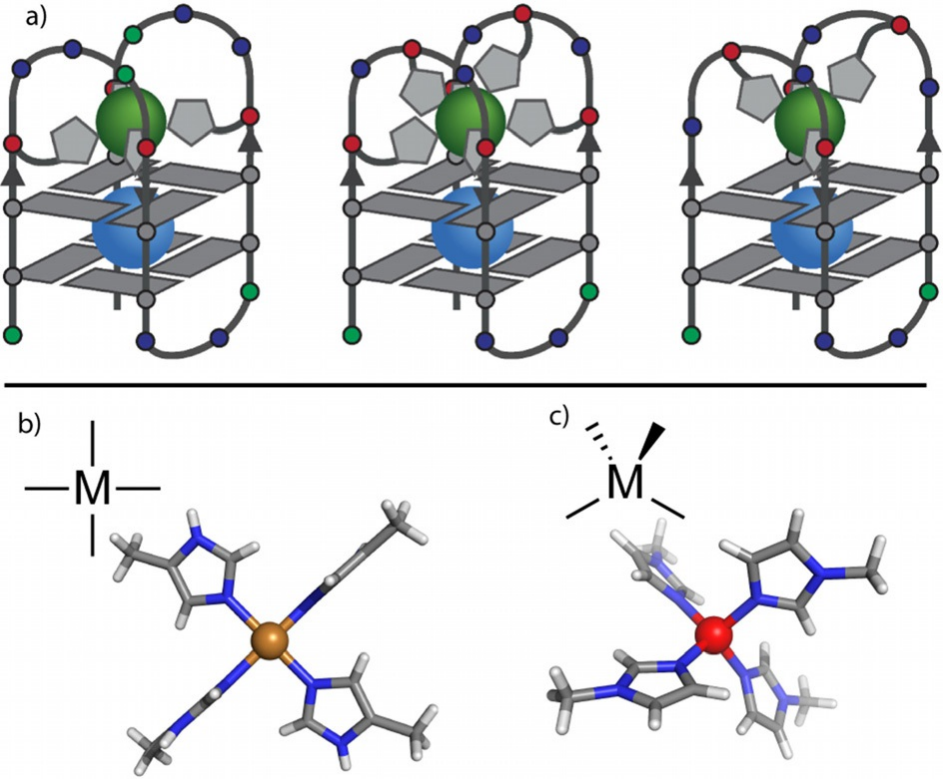
a) Schematic representation of the folded structures of htel**L**_4_, htel**L**_6_ and htel**L**_4_C. Reported X‐ray structures of b) a square‐planar [Cu^II^(4‐methyl‐imidazole)_4_] complex and c) a tetrahedral [Zn^II^(*N*‐methyl‐imidazole)_4_] complex.^18^

In contrast to Cu^II^ and Ni^II^, the results with Zn^II^ and Co^II^ were more complex. Although for htel**L^2*S*^**_4_ only weak stabilizations were observed, addition of Zn^II^ and Co^II^ to htel**L^2*S*^**_6_ resulted in a significant increase of the melting temperature [Δ*T*_m_ (Zn) = +8 °C, Δ*T*_m_ (Co) = +8 °C]. To explain the increased thermal stabilization for Zn^II^ two possible explanations were considered. Either Zn^II^ prefers the coordination of six imidazole ligands or four of the contained ligands in htel**L^2*S*^**_6_ are better positioned to serve a tetrahedral coordination geometry. To clarify this, two new sequences called htel**L^2^**_4_B and htel**L^2^**_4_C, containing four ligands each in the differing loop positions, were synthesized (see Table 2 for sequences). In contrast to htel**L^2^**_4_, two of the four ligands were placed in the loops and two close to the terminal G‐tetrad, prearranging them in a tetrahedral geometry (Figure 5). Surprisingly, this change already led to significant higher thermal stabilities of htel**L^2*S*^**_4_B (*T*_m_=37 °C) and htel**L^2*S*^**_4_C (*T*_m_=42 °C) compared to htel**L^2*S*^**_4_ (*T*_m_=33 °C) in the absence of transition metals, highlighting the crucial rule of the loop composition on G‐quadruplex stabilities. After addition of 1 equiv Zn^II^ to htel**L^2*S*^**_4_B (Δ*T*_m_=+19 °C) and htel**L^2*S*^**_4_C (*T*_m_=+21 °C), indeed quite high thermal stabilizations were observed, unprecedented in the context of metal‐mediated base pairs with Zn^II^.^19^ Interestingly, for Cu^II^ the opposite effect was observed, showing a lower thermal stabilization when added to htel**L^2*S*^**_4_C (*T*_m_=+21 °C) compared to htel**L^2*S*^**_4_ (Δ*T*_m_=+23 °C; Supporting Information, Table S3). At first glance, this effect looks small, but it is remarkable considering the Irving–Williams series, according to which Cu^II^ usually shows higher complexation constants compared to those shown by Zn^II^.^20^ Considering that the number of imidazole ligands is constant in all three sequences, the changes in thermal stability can be assigned to the spatial arrangement of the ligands. This finding opens up a second layer of control besides varying the number of ligands to predesign coordination environments within folded G‐quadruplexes to selectively suit a choice of metal cations and purposes.

**Table 2:** Sequences investigated in this study. Marked in red are the incorporated ligands (**L**).

	**Sequence 5′**→**3′**
G_4_ **L**	**L**GG GG
G_5_ **L**	**L**GG GGG
htel	AGG GTT AGG GTT AGG GTT AGG G
htel**L**_2_	AGG TTT A**L**G GTT AGG **L**TT ATG G
htel**L**_2_B	AGG GT**L** TGG GTT AGG GT**L** TGG G
htel**L**_3_	AGG **L**TT A**L**G GTT AGG **L**TT ATG G
htel**L**_3_B	AGG GT**L** TGG GTT AGG G**L**T **L**GG G
htel**L**_4_	AGG **L**TT A**L**G GTT AGG **L**TT A**L**G G
htel**L**_4_B	AGG T**L**T **L**GG TTA GG**L** T**L**A GG
htel**L**_4_C	AGG T**L**T **L**GG TTA GGT **L**T**L** GG
htel**L**_5_	AGG **L**T**L** T**L**G GTT AGG **L**TT A**L**G G
htel**L**_6_	AGG **L**T**L** T**L**G GTT AGG **L**T**L** T**L**G G
htel**L**_7_	AGG **L**T**L** **L**T**L** GGT TAG G**L**T **L**T**L** GG

**Figure 6:**
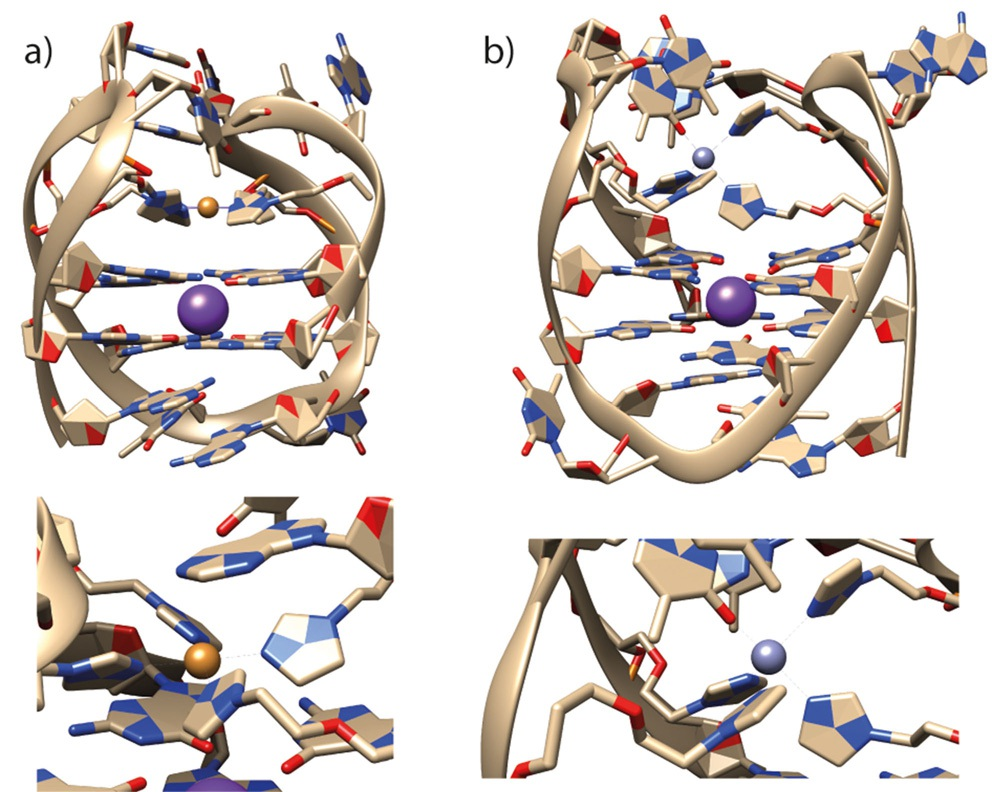
Representative pictures from MD simulations of htel**L^2*S*^**_4_ in complex with a) Cu^II^ and b) Zn^II^.

To gain more detailed structural insights, MD simulations of htel**L^2*S*^**_4_ with bound Cu^II^ and Zn^II^ cations were prepared (Figure 6). Interestingly, although for Cu^II^ the expected square‐planar coordination is observed, simulations with Zn^II^ show coordination of a fifth ligand, resulting in a trigonal‐bipyramidal arrangement. As additional ligand, either a thymine residue from the loop region coordinating with its C4 carbonyl oxygen or a water molecule was observed. For further MD simulation data, including sequences htel**L^2S^**_4_C and htel**L^2S^**_6_ with and without bound Cu^II^ and Zn^II^ cations, see the Supporting Information.

## Conclusions

The incorporation of two bioinspired imidazole ligandosides with chiral backbones into tetramolecular and unimolecular DNA G‐quadruplex structures was established. We showed that the imidazole‐modified systems fold in an antiparallel topology. The G‐quadruplexes were found to complex various transition‐metal cations, such as Cu^II^, Ni^II^, Co^II^ and Zn^II^, which was expressed by an increase in their thermal stability. The proposed metal complexes were investigated by native ESI‐MS and trapped ion mobility spectrometry methods that turned out to be in accordance with folded G‐quadruplex structures. In addition, MD simulations were performed to gain structural insights into the investigated systems, illustrate how metal coordination rigidifies the G‐quadruplex structures and how ligand arrangement influences metal complexation. To show the robustness of the presented system, different counts of ligand **L^2^** (two to seven) were incorporated in unimolecular G‐quadruplexes. Ligand number variation enabled the fine‐tuning of metal affinities with respect to the typical coordination number. Thus Cu^II^, usually coordinated by four ligands, showed the highest thermal stabilization after incorporation of four ligands in contrast to Ni^II^ or Co^II^, which were found to prefer six ligands. In addition to the number of incorporated ligands, the system also enables the arrangement of ligands in certain geometries as shown for Zn^II^, which prefers a tetrahedral arrangement, whereas Cu^II^ favors a square‐planar binding site.

The herein introduced sophisticated control over the design of specific coordination environments inside cavities formed from folded DNA structures shows potential to facilitate the engineering of highly complex coordination spheres with metallo‐enzyme‐like activities. The simple phosphoramidite‐based approach will allow for the expansion of the family of DNAzymes with members showing unprecedented functionality.^21^ Our concept even allows to introduce an additional level of complexity by mixing different types of ligands, such as carboxylate or thiol groups, with the herein presented imidazole moieties, to create nonstatistical heteroleptic environments, a challenge that we are currently pursuing.

## Conflict of interest

The authors declare no conflict of interest.

## Supporting information

As a service to our authors and readers, this journal provides supporting information supplied by the authors. Such materials are peer reviewed and may be re‐organized for online delivery, but are not copy‐edited or typeset. Technical support issues arising from supporting information (other than missing files) should be addressed to the authors.

Supplementary
