# Supplementary material for: Tailored Transition‐Metal Coordination Environments in Imidazole‐Modified DNA G‐Quadruplexes
Source: Chemistry. 2019 Oct 10;25(61):13987–93. doi: 10.1002/chem.201903445 (PMC6899475; doi:10.1002/chem.201903445)
Supplement: Supplementary file 1 — Supplementary [file CHEM-25-13987-s001.pdf]

CHEMISTRY
A European Journal

Supporting Information

Tailored Transition-Metal Coordination Environments in Imidazole-Modified DNA G-Quadruplexes

Philip M. Punt and Guido H. Clever*[a]

chem_201903445_sm_miscellaneous_information.pdf

Table of Contents

1 Synthesis 2

DMT-protected glycidol **2** 2

Glycol-based imidazole nucleoside **3** 3

Phosphoramidite **4** (*R/S*) 3

Glycol-based imidazole nucleoside **5** 4

2 Solid phase DNA synthesis 5

Reversed phase HPLC 6

3 UV/VIS spectroscopy 7

Thermal difference spectra (TDS) 7

4 Thermal denaturation experiments 17

5 Circular dichroism (CD) measurements 30

6 Native ESI-MS 43

7 MD simulations 52

Partial charges (RESP) 52

8 Oligonucleotide Analytics 59

9 Literature 71

1 Synthesis

All chemicals were obtained from commercial sources and used without further purification. Gel permeation chromatography (GPC) purification of ligands was performed on a *LC-9210 II NEXT* system with $CHCl_3$ (HPLC grade) as eluent. NMR measurements were conducted at 298 K on *Avance-600* and *Avance-700* instruments from *Bruker* and on 500 MHz *Bruker Avance neo* NMR. Chemical shifts for ^1H and ^{13}C are reported in ppm on the δ scale; ^1H and ^{13}C signals were referenced to the residual solvent peak. The following abbreviations are used to describe signal multiplicity for ^1H-NMR spectra: s: singlet, d: doublet, t: triplet, dd: doublet of doublets; dt: doublet of triplets; m: multiplet, br: broad. High resolution electrospray ionization mass spectrometry (ESI HRMS) was performed on *Bruker Apex IV FTICR*, *Bruker compact* and *Bruker timsTOF ESI* mass spectrometers.

DMT-protected glycidol **2**

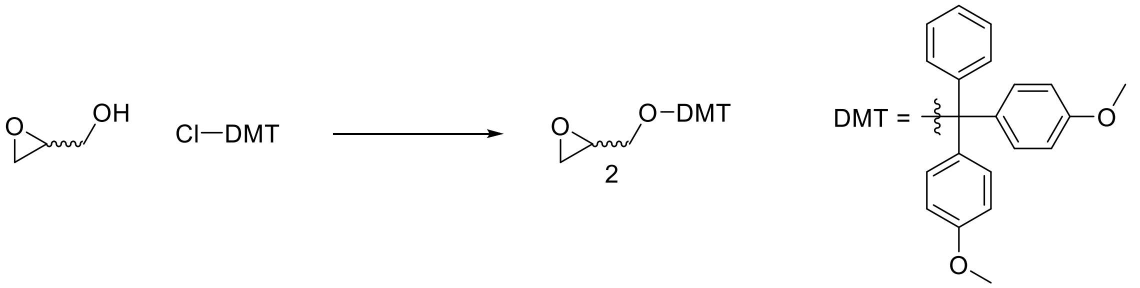

DMT-protected glycidol **2** was synthesized enantiomerically pure according to a modified literature procedure.[1] To a solution of (*R* or *S*)-glycidol (1.0 mL, 15.1 mmol, 1 equiv.) and Et_3N (5.4 mL, 40.7 mmol, 2.7 equiv.) in CH_2Cl_2 (34 mL) DMT-Cl (4,4'-Dimethoxytrityl chloride; 6.45 g, 19 mmol, 1.26 equiv.) was added and the mixture stirred at 20 °C for 16 h. The reaction mixture was washed with half saturated aq. $NaHCO_3$ (50 mL) and extracted with CH_2Cl_2 (2 × 30 mL). The combined organic layers were dried over $MgSO_4$ and the solvent was removed under reduced pressure to afford a dark red oil. The product was purified by column chromatography (*n*-pentane/EtOAc, 98/2 → *n*-pentane/EtOAc, 90/10) to afford the DMT-protected glycidol **2** as a viscous colourless oil.

^{1}H-NMR (600 MHz, $CDCl_3$): σ (/ppm) = 2.63 (dd, 1H), 2.78 (dd, 1H), 3.13 (dd, 2H), 3.32 (dd, 1H), 3.79 (s, 6H), 6.82 – 6.85 (m, 4H), 7.20 – 7.23 (m, 1H), 7.28 – 7. 31 (m, 2H), 7.34 – 7.37 (m, 4H), 7.46 – 7.48 (m, 2H).

^{13}C-NMR (150 MHz): σ (/ppm) = 45.0, 51.5, 55.6, 64.9, 86.5, 113.5, 127.1, 128.2, 128.5, 130., 136.4, 145.1, 158.8.

Chemical formula: $C_{24}H_{24}O4$

Molecular weight: 376.45 g mol^{-1}

Yield: 5.1 g, 13.55 mmol, 90 %

Glycol-based imidazole nucleoside **3**

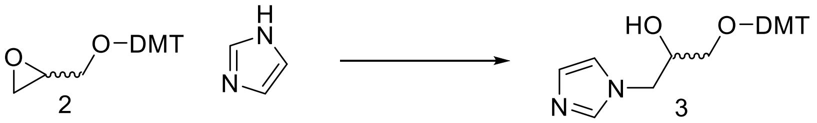

Glycol-based imidazole nucleoside **3** was prepared according to a previous report.[2] To a solution of imidazole (0.54 g, 8 mmol, 2 equiv.) in 1,4-dioxane (7.5 mL) a solution of **2** (*R* or *S*) in 1,4-dioxane (2.5 mL) was added. The reaction was stirred at 80 °C for 21 h until all starting material was consumed. The reaction mixture was washed with half saturated aq. $NaHCO_3$ (40 mL) and extracted with CH_2Cl_2 (2 × 30 mL). The combined organic layers were dried over $MgSO_4$ and the solvent was removed under reduced pressure to afford a yellow oil. The product was purified by flash chromatography (CH_2Cl_2/MeOH, 100/0 → CH_2Cl_2/MeOH, 90/10) followed by gel permeation chromatography ($CHCl_3$) to give the product (**3**) as a white foamy solid.

^{1}H-NMR (600 MHz, $CDCl_3$): σ (/ppm) = 3.14 (dd, 1H); 3.22 (dd, 1H); 3.6 (sbroad, 1H); 3.79 (s, 6H); 3.91 – 3.95 (m, 1H), 3.95 (dd, 1H); 4.08 (dd, 1H); 6.82 – 6.86 (m, 6H); 7.22-7.24 (m, 1H); 7.29-7.32 (m, 7H); 7.41-7.43 (m, 2H).

^{13}C-NMR (150 MHz): σ (/ppm) = 50.6, 55.6, 64.8, 70.5, 68.8, 113.6, 120.0, 127.3, 128.3, 128.4, 129.3, 130.3, 136.0, 138.0 144.8, 159.0.

**Chemical formula:**	$C_{27}H_{28}N_2O_4$
**Molecular weight:**	444.53 g mol^{-1}
**Yield:**	1.16 g, 2.61 mmol, 66 %
**HRMS** (ESI, pos.,	Calc. $[M+H]^+$: 445.2122
ACN/water, 1/1, 0.1% TFA)	Found: m/z = 303.1365 $[DMT]^+$, 445.2109 $[M+H]^+$

Phosphoramidite **4** (*R/S*)

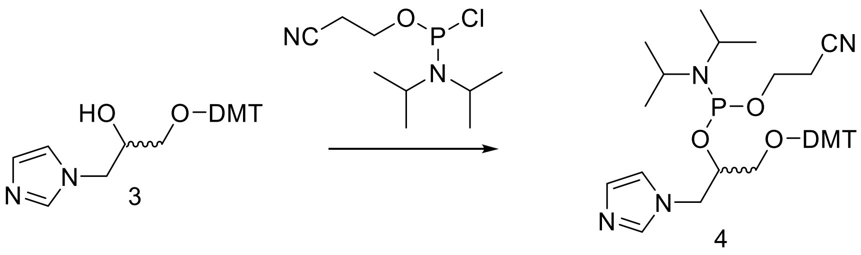

A solution of compound **3** (0.4 g, 0.82 mmol, 1 equiv.) and DIPEA (857 µL, 4.92 mmol, 6 equiv.) in dry CH_2Cl_2 was stirred under argon atmosphere. To this solution, 2-cyanoethyl *N,N*-diisopropyl chlorophosphoramidite (233 mg, 220 µL, 0.984 mmol, 1.2 equiv.) was added dropwise and the reaction mixture stirred for 90 minutes. After completion, the solvent was removed under reduced pressure to afford the crude phosphoramidite **4** as a slightly yellow oil with an estimated yield of 80 %. The compound was used for solid phase synthesis without further purification.

Chemical formula: $C_{36}H_{45}N_4O_5P$

Molecular weight: 644.75 g mol^{-1}

^{31}P-NMR (242 MHz, $CDCl_3$): σ /ppm =149.0, 149.8.

Glycol-based imidazole nucleoside **5**

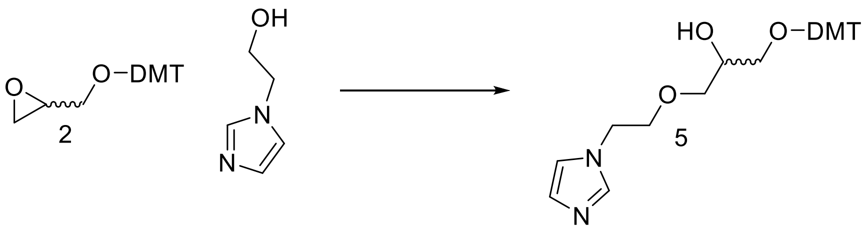

A suspension of NaH (60 % in mineral oil; 200 mg, 5 mmol, 4 equiv.) in DMF (2 mL) was degassed three times before a solution of *N*-hydroxyethyl imidazole (0.3 g, 2.66 mmol, 2 equiv.) in DMF (4 mL) was dropwise added under argon at 0 °C. The suspension was allowed to reach 20 °C and was stirred for 60 min until no H_2 formation was observed. Then a solution of compound **2** (*R* or *S* enantiomer) (0.5 g, 1.33 mmol, 1 equiv.) in DMF (4 mL) was dropwise added and the reaction was heated up to 40 °C for 16 h. After all starting material was consumed, the reaction mixture was quenched with an aq. solution of $NaHCO_3$ (50 mL) at 0°C. The reaction mixture was extracted with CH_2Cl_2 (3 × 50 mL) and the combined organic layers were dried over $MgSO_4$. The solvent was removed under reduced pressure to afford a yellow oil. The product was purified by flash chromatography (CH_2Cl_2/MeOH, 100/0 → CH_2Cl_2/MeOH, 90/10) followed by gel permeation chromatography ($CHCl_3$) to give the product (**5**) as a foamy solid.

^{1}H-NMR (700 MHz, $CDCl_3$): σ /ppm = 2.34 (s_{broad}, 1H, OH); 3.15 - 3.19 (m, 2H,); 3.47 (dd , 1H); 3.51 - 3.53 (dd, 1H); 3.66 - 3.71 (m, 2H); 3.79 (s, 6H); 3.90 - 3.93 (m, 1H); 4.03 - 4.07 (m, 2H); 6.82 – 6.84 (m, 4H); 6.89 (s_{broad}, 1H); 7.01 (s_{broad}, 1H); 7.2 – 7.23 (m, 1H); 7.28 – 7.30 (m, 2H); 7.29 - 7.31 (m, 4H); 7.41 – 7.42 (m, 2H), 7.46 (s_{broad}, 1H).

^{13}C-NMR (175 MHz, $CDCl_3$): σ /ppm = 47.3, 55.6, 64.7, 70.2, 71.0, 73.1, 86.5, 113.5, 119.7, 127.2, 128.2, 128.4, 129.6, 130.4, 136.22, 137.7, 145.0, 158.9.

Chemical formula: $C_{29}H_{32}N_2O_5$

Molecular weight: 488.58 g mol^{-1}

Yield: 0.48 g, 0.98 mmol, 74 %

MS (ESI, pos., ACN/water, 1/1, 0.1% TFA): Calc. $[M+H]^+$: 489.2384

Found: *m/z* = 303.136 $[DMT]^+$, 489.2381 $[M+H]^+$

Phosphoramidite **6** (*R/S*)

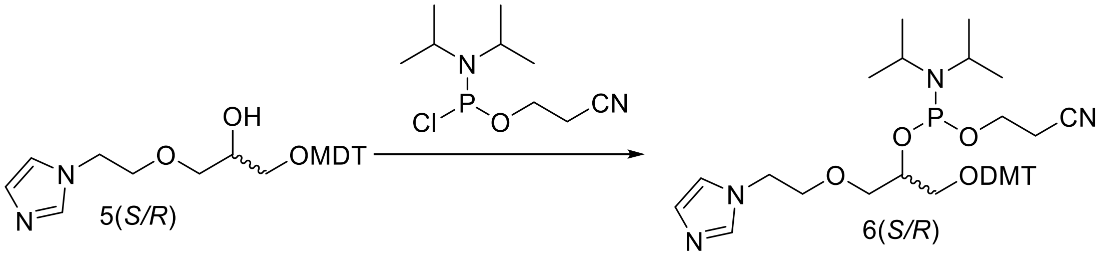

A solution of compound **5** (0.4 g, 0.82 mmol, 1 equiv.) and DIPEA (857 µL, 4.92 mmol, 6 equiv.) in dry CH_2Cl_2 was stirred under argon atmosphere. To the solution 2-cyanoethyl *N,N*-diisopropylchlorophosphoramidite (233 mg, 220 µL, 0.984 mmol, 1.2 equiv.) was added dropwise and the reaction mixture stirred for 90 minutes. After completion, the solvent was removed under reduced pressure to afford the crude phosphoramidite **6** as a slightly yellow oil with an estimated yield of 80 %. The compound was used for solid phase synthesis without further purification.

Chemical formula: $C_{38}H_{49}N_4O_6P$

Molecular weight: 688.80 g mol^{-1}

^{31}P-NMR (242 MHz, $CDCl_3$): σ /ppm =149.2, 149.4.

2 Solid phase DNA synthesis

Solid phase DNA synthesis was carried out on a *K&A Laborgeräte GbR H-8* synthesizer using the standard phosphoramidite method on a 1 µmol scale. Cartridges (*Biosearch Technologies* or *Link Technologies*) for solid phase synthesis were self-packed with CPG (1000 Å, 33 µmol/g) purchased from *Sigma Aldrich*. First, the cartridge was treated three times with 3 % DCA (dichloroacetic acid in CH_2Cl_2). Second, the coupling step was performed by mixing activator (5-(benzylthio)-1*H*-tetrazole, BTT) and phosphoramidite in a 1:1 ratio. The incubation time for the standard canonical nucleotides was 0.5 min whereas for artificial nucleotides the coupling time was extended to 3.5 min. Next, unreacted 5'-OH groups were acetylated with a 1:1 mixture of capping solutions Cap A and Cap B followed by a final oxidation step (see table 1). After each step, the cartridge was washed with acetonitrile followed by a drying step using argon gas. The described cycle was repeated for every incorporated base.

Apart from the oxidizer solution, all solvents were bought in anhydrous quality. Standard 4,4'-dimethoxytrityl (DMT) and cyanoethyl (CE) protected phosphoramidites (DMTdT-CEP, DMT-dG(iBu)-CEP, DMT-dA(Bz)-CEP) were purchased from *Sigma Aldrich* and were dissolved for DNA synthesis in dry acetonitrile (0.1 M).

Table **1**. Solutions used in DNA solid phase synthesis. All solutions were prepared under argon 16 h hours before solid phase synthesis and stored at 4°C. Activator and capping solutions were stored over molecular sieves (3 Å).

**Step**	**Composition**
Detritylation	3 % DCA in CH_2Cl_2
Activation	0.3 M BTT in ACN
Cap A	THF/lutidine/acetic anhydride, 8/1/1
Cap B	10 % (w/v) N-methyl imidazole in THF
Oxidation	50 mM I_2 in THF/pyridine/water, 78/20/2

After DNA solid phase synthesis, the oligonucleotide was cleaved from the solid support using concentrated NH_4OH at 55 °C for 16 h. The sample was filtered through *VWR Centrifugal filters*. Solvent volume was reduced in a *S-Concentrator BA-VC-300H from H. Saur Laborgeräte* to ~300 µL and purified by reversed phase HPLC. After purification, the DMT-protection group was removed and the sample desalted on SepPak C_{18} cartridges. Desalted oligonucleotides were lyophilized and stored at -20 °C until use.

Concentrations were individually determined at 260 nm using a *Thermo Scientific Nanodrop One* instrument. The absorption of the imidazole modifications at 260 nm could be neglected.

Reversed phase HPLC

Reversed-phase HPLC was performed on *Agilent Technologies 1260 Infinity I* or *II* HPLC systems equipped with *Agilent Zorbax 300 SB-C18 4.5/10 x 250 mm* or *Macherey-Nagel EC 250 x 4.6 NUCLEODUR 100-5 C18ec* columns. The flow rate was set to 0.75/1 (analytical) or 2.5 mL min^{-1} (semi preparative). Samples were filtered through *VWR Centrifugal filters* to ensure clear solutions and the column was equilibrated against buffer A (0.05 M TEAA pH 7 (triethylammonium acetate)). After equilibration, samples were analysed using a standard gradient starting with buffer A (100 %) going to buffer B (80 %) over 30 min. Typical sample volumes were 10 µL with DNA concentrations ranging from 100 to 200 µM. Buffer A: Aqueous solution of 0.05 M TEAA (triethylammonium acetate) adjusted to pH 7; buffer B: 30 % v/v 0.05 M TEAA pH 7 in 70% v/v acetonitrile.

Table **2**. G-quadruplex forming sequences

	**Sequence 5' → 3'**
G_4**L**	LGG GG
G_5**L**	LGG GGG
htel	AGG GTT AGG GTT AGG GTT AGG G
htelL_2	AGG TTT ALG GTT AGG LTT ATG G
htelL_3	AGG LTT ALG GTT AGG LTT ATG G
htelL_2B	AGG GTL TGG GTT AGG GTL TGG G
htelL_3B	AGG GTL TGG GTT AGG GLT LGG G
htelL_4	AGG LTT ALG GTT AGG LTT ALG G
htelL_4B	AGG TLT LGG TTA GGL TLA GG
htelL_4C	AGG TLT LGG TTA GGT LTL GG
htelL_5	AGG LTL TLG GTT AGG LTT ALG G
htelL_6	AGG LTL TLG GTT AGG LTL TLG G
htelL_7	AGG LTL LTL GGT TAG GLT LTL GG

3 UV/VIS spectroscopy

All UV/VIS based measurements were carried out on *Jasco V-650* and *Jasco V-750* absorption spectrometers in quartz glass cuvettes. Samples were prepared with 3.75 (tetramolecular) and 1.875 µM (unimolecular) ssDNA in 100 mM NaCl (tetramolecular) or KCl (unimolecular) unless stated differently, 10 mM LiCaCo pH 7.2 and, if present, 1 equiv. transition metal cations (refers to 0.94 µM for tetramolecular and 1.88 µM for unimolecular G-quadruplexes). As transition metal sources $CuSO_4$, $NiSO_4$, ZnI_2 and $Co(NO_3)_2$ were used. To form the G-quadruplexes, solutions were heated to 85 °C and cooled down to 4 °C at 0.5 °C min^{-1}. Tetramolecular samples were then frozen at –20 °C for one hour to ensure full G-quadruplex formation.[31]

Thermal difference spectra (TDS) were recorded from 220 to 350 nm by subtracting the low temperature spectrum (4 °C) from the high temperature spectrum (85 °C). The absorption at 350 nm was then set to 0.

Thermal denaturation experiments were carried out to determine the thermal stability expressed in the melting temperature T_m. Therefore, the change in absorbance, as the temperature was increased with 0.5 °C min^{-1}, was followed at 295 nm indicative for G-quadruplex denaturation and at 350 nm as control. Note that for unimolecular G-quadruplexes at every temperature an equilibrium between folded and unfolded species is given. This is not true for tetramolecular G-quadruplexes which is the reason why the melting temperature for tetramolecular G-quadruplexes depends on the heating rate. The spectrometer bandwidth was set to 2 nm. To control the temperature, a cuvette containing water was equipped with a temperature probe connected to the spectrometer. Water evaporation from the samples was avoided by addition of small amounts of silicon oil. To ensure equal heating rates between different experiments, always five samples were measured at a time. The measured absorbance was plotted against the temperature after the absorbance at 350 nm was subtracted to remove background absorption. Spectra were normalized to fraction folded values between 1 and 0 corresponding to fully folded or unfolded G-quadruplexes, respectively.

Thermal difference spectra (TDS)

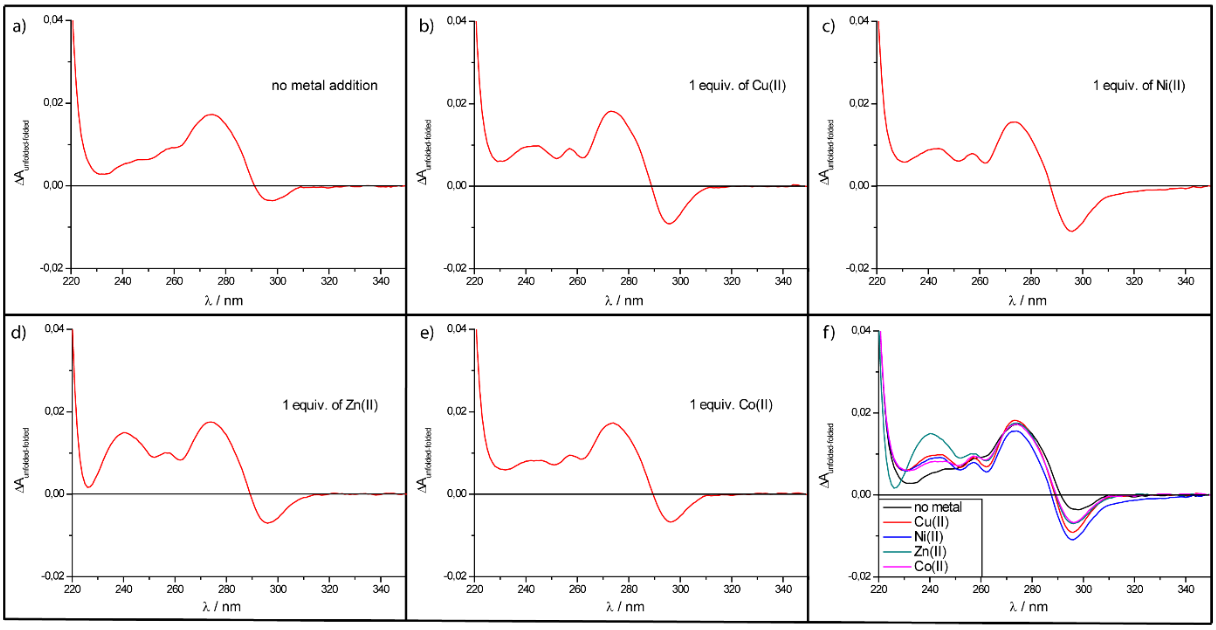

Figure 1. TDS of G_4**L**^{2S} recorded from 220 to 350 nm: a) without metal addition, after addition of 1 equiv. b) Cu(II), c) Ni(II), d) Zn(II) and e) Co(II); f) superposition of a)-e).

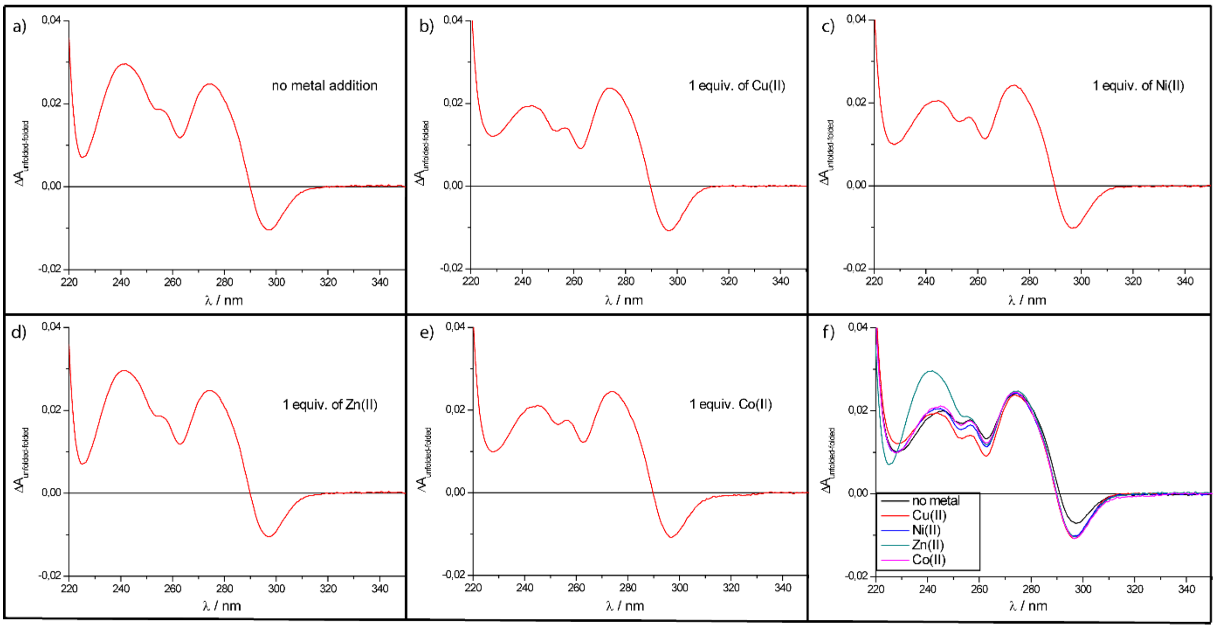

Figure 2. TDS of G_4**L**^{2R} recorded from 220 to 350 nm: a) without metal addition, after addition of 1 equiv. b) Cu(II), c) Ni(II), d) Zn(II) and e) Co(II); f) superposition of a)-e).

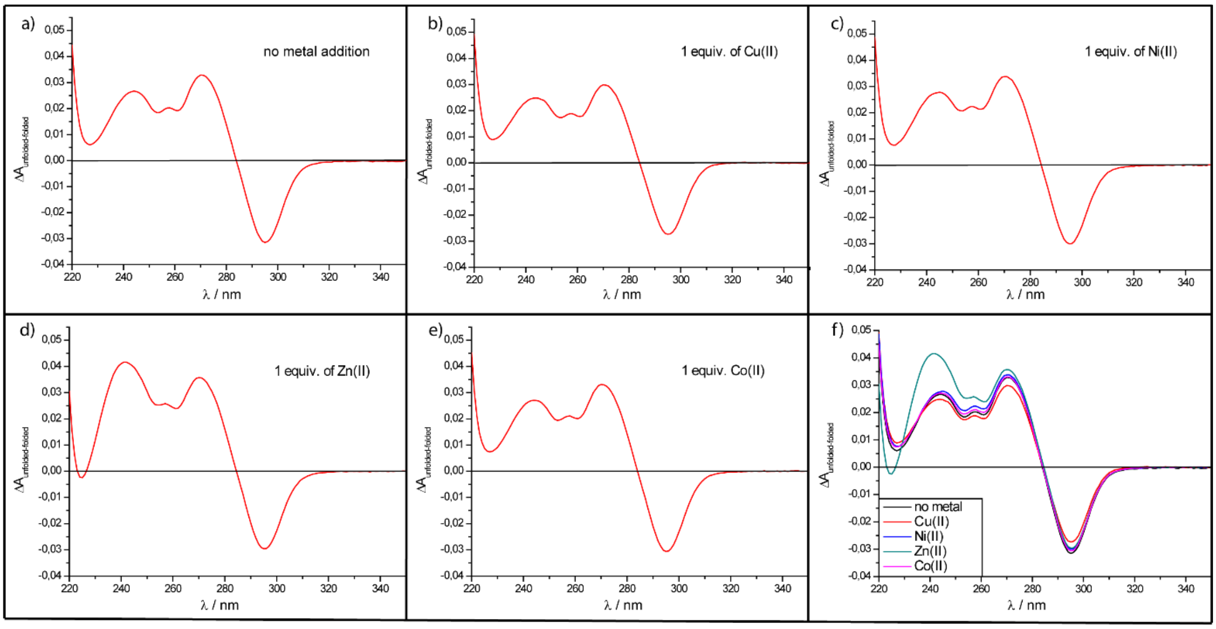

Figure 3. TDS of htel**L**$^{1R}{}_4$ recorded from 220 to 350 nm: a) without metal addition, after addition of 1 equiv. b) Cu(II), c) Ni(II), d) Zn(II) and e) Co(II); f) superposition of a)-e).

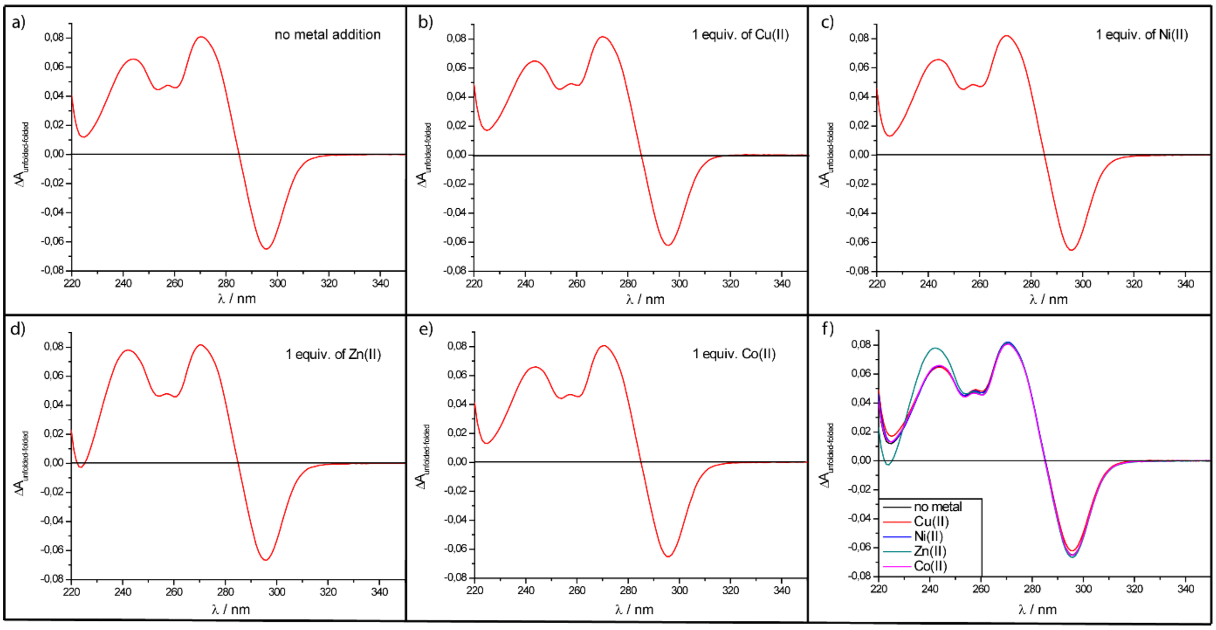

Figure 4. TDS of htel**L**$^{1S}_4$ recorded from 220 to 350 nm: a) without metal addition, after addition of 1 equiv. b) Cu(II), c) Ni(II), d) Zn(II) and e) Co(II); f) superposition of a)-e).

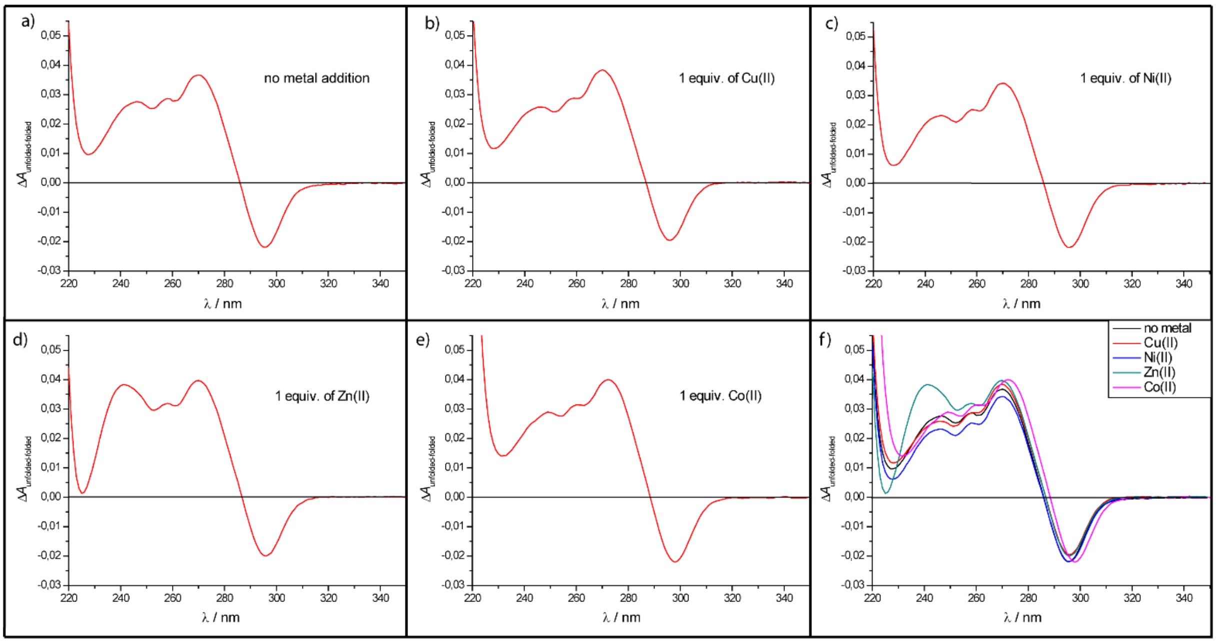

Figure 5. TDS of htel**L**$^{2S}_2$ recorded from 220 to 350 nm: a) without metal addition, after addition of 1 equiv. b) Cu(II), c) Ni(II), d) Zn(II) and e) Co(II); f) superposition of a)-e).

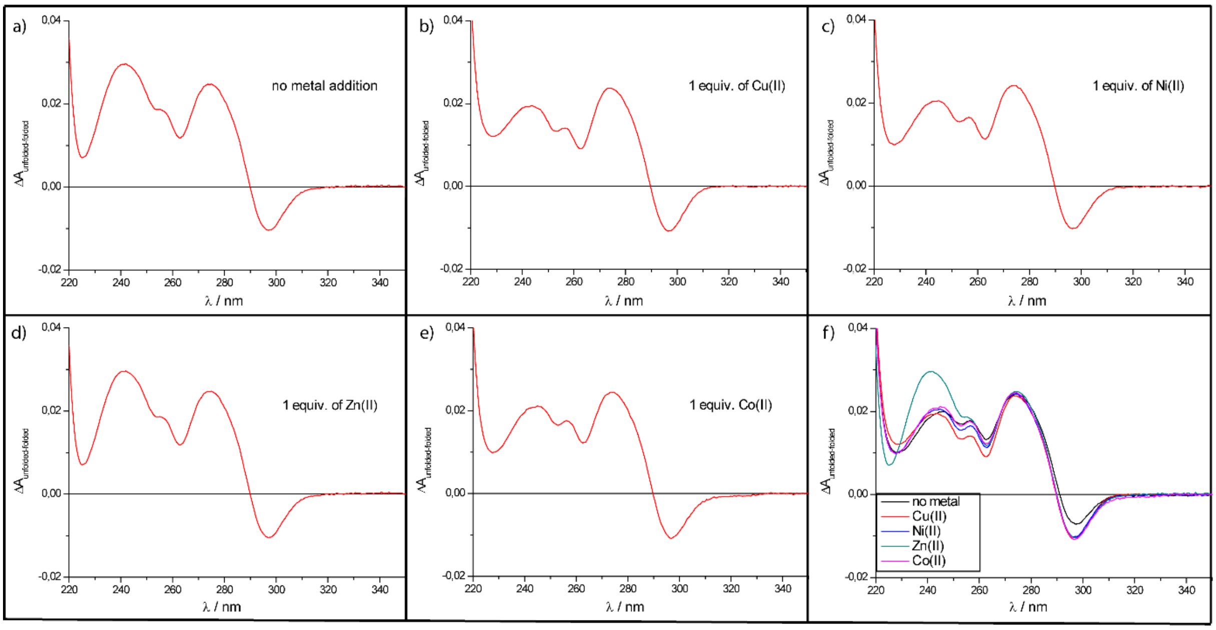

Figure 8. TDS of htel$\mathbf{L}^{2R}_2$ recorded from 220 to 350 nm: a) without metal addition, after addition of 1 equiv. b) Cu(II), c) Ni(II), d) Zn(II) and e) Co(II); f) superposition of a)-e).

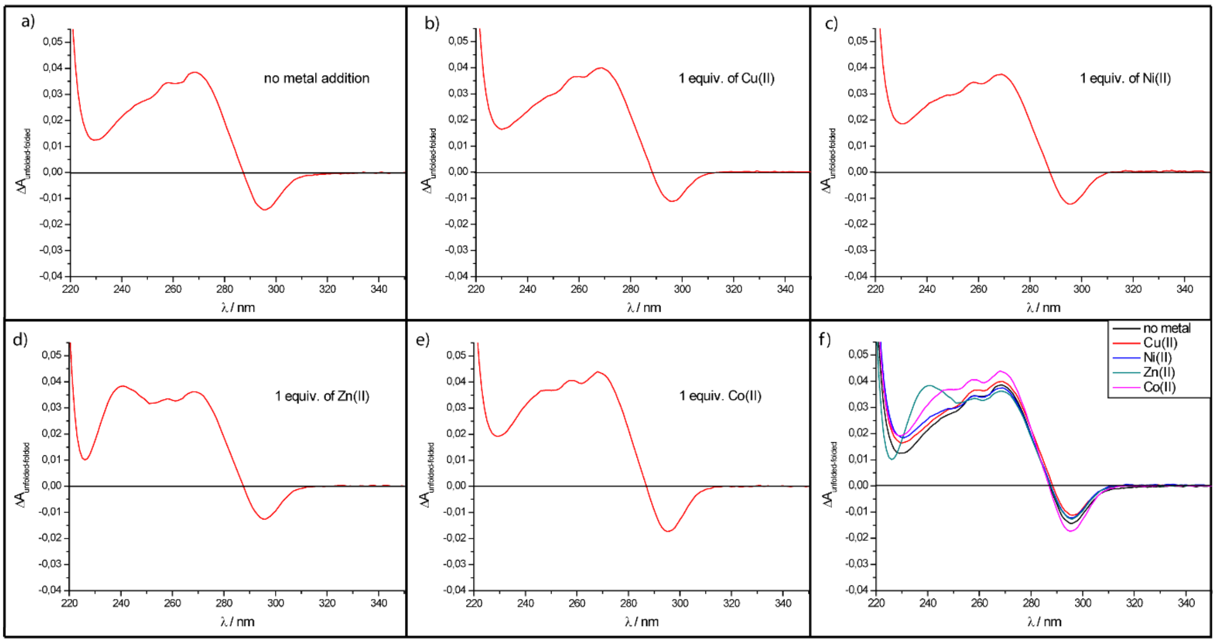

Figure 6. TDS of htel$\mathbf{L}^{2R}_3$ recorded from 220 to 350 nm: a) without metal addition, after addition of 1 equiv. b) Cu(II), c) Ni(II), d) Zn(II) and e) Co(II); f) superposition of a)-e).

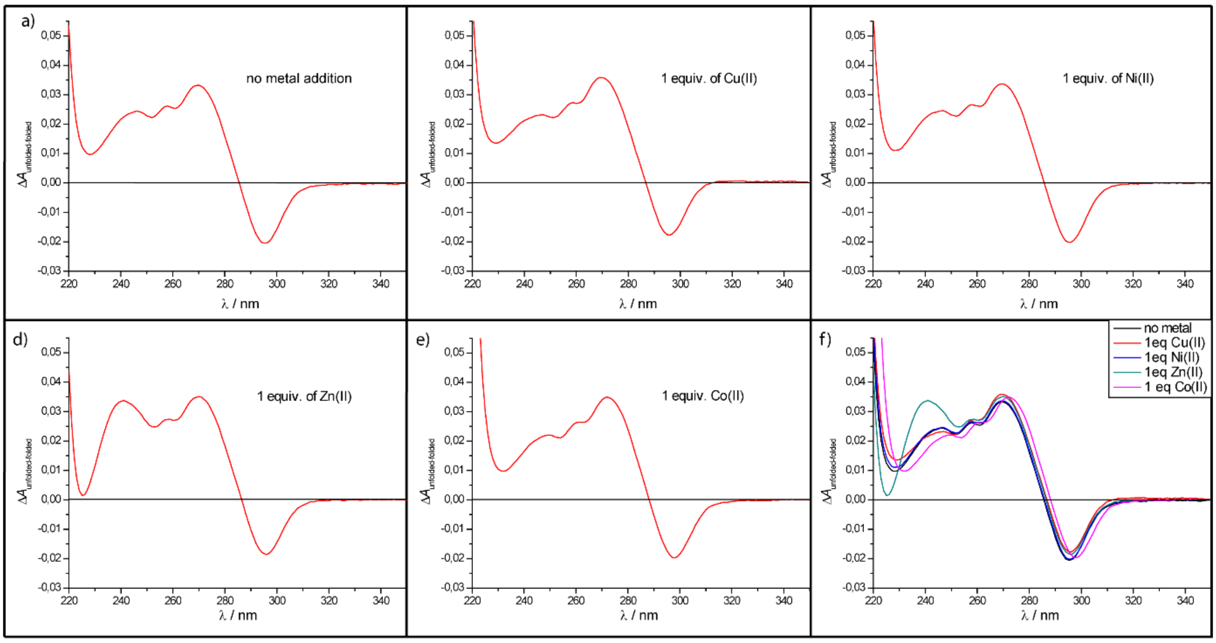

Figure 7. TDS of htel$\mathbf{L}^{2S}_3$ recorded from 220 to 350 nm: a) without metal addition, after addition of 1 equiv. b) Cu(II), c) Ni(II), d) Zn(II) and e) Co(II); f) superposition of a)-e).

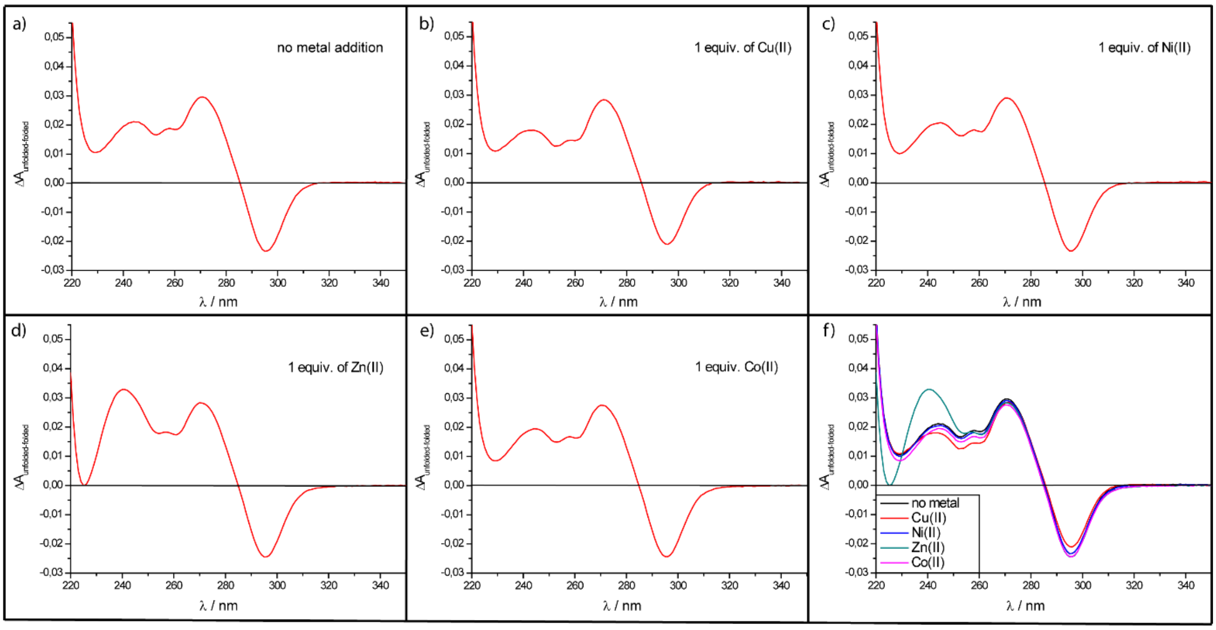

Figure 8. TDS of htel$\mathbf{L}^{2R}_4$ recorded from 220 to 350 nm: a) without metal addition, after addition of 1 equiv. b) Cu(II), c) Ni(II), d) Zn(II) and e) Co(II); f) superposition of a)-e).

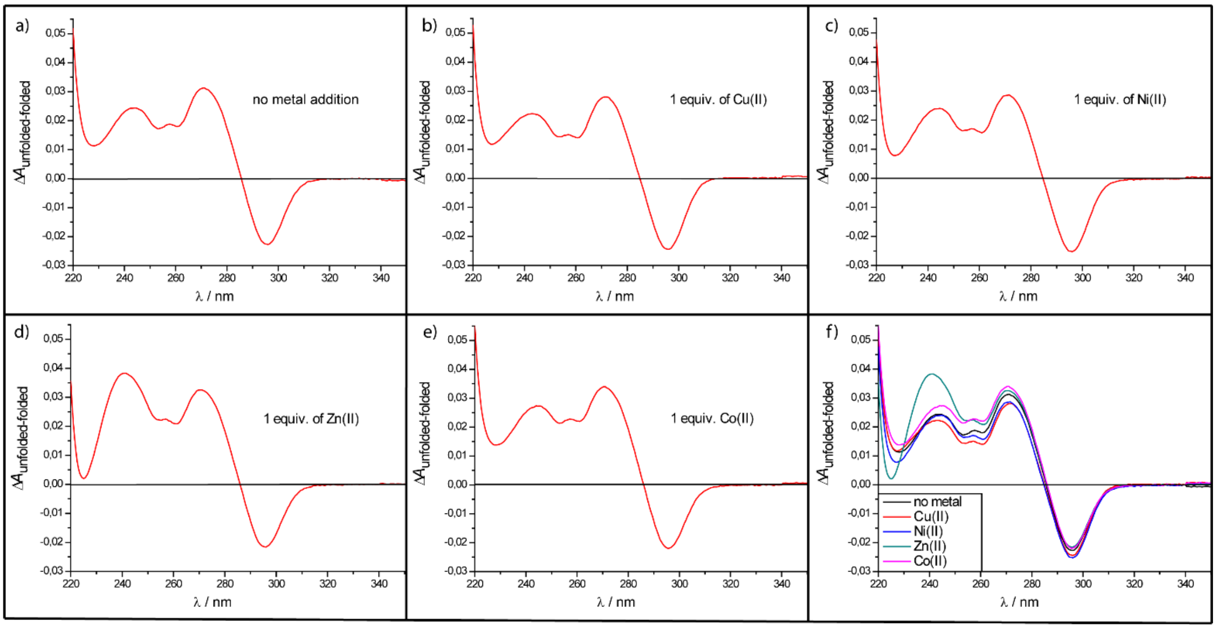

Figure 9. TDS of htel$\mathbf{L}^{2S}{}_4$ recorded from 220 to 350 nm: a) without metal addition, after addition of 1 equiv. b) Cu(II), c) Ni(II), d) Zn(II) and e) Co(II); f) superposition of a)-e).

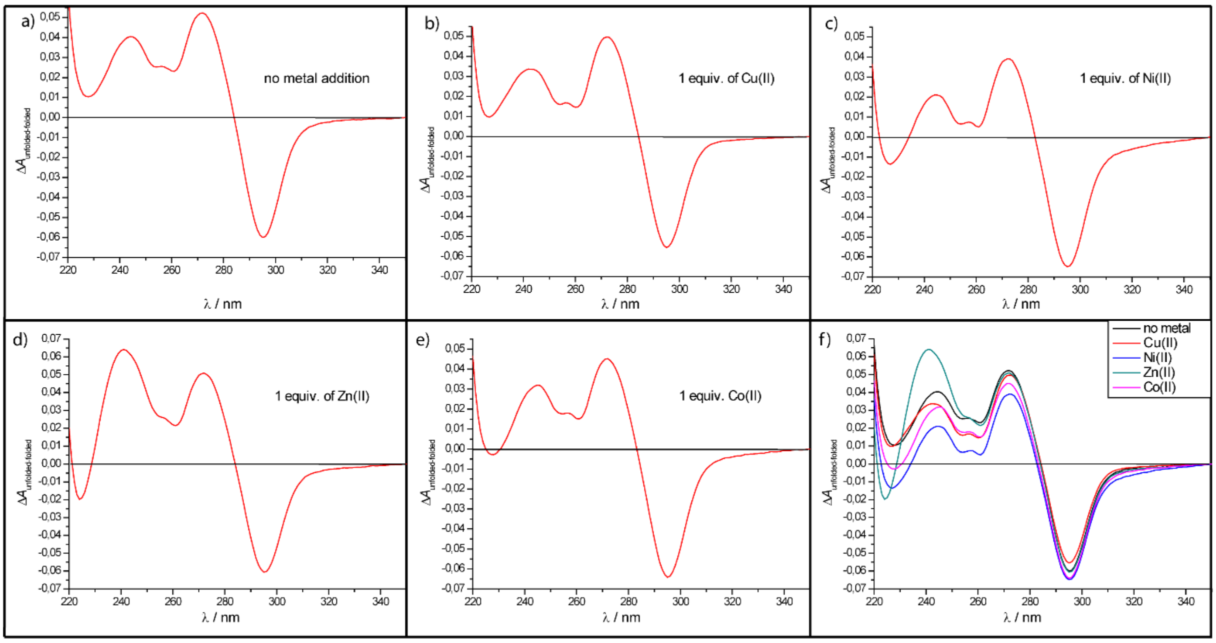

Figure 10. TDS of htel$\mathbf{L}^{2R}{}_5$ recorded from 220 to 350 nm: a) without metal addition, after addition of 1 equiv. b) Cu(II), c) Ni(II), d) Zn(II) and e) Co(II); f) superposition of a)-e).

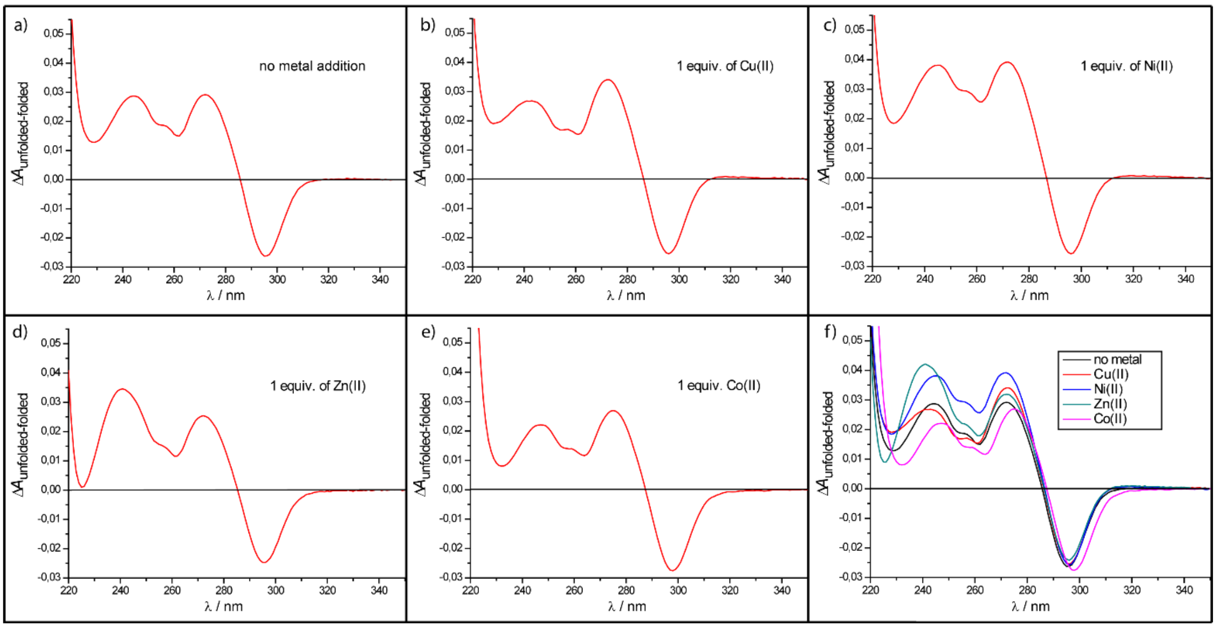

Figure 11. TDS of htel$\mathbf{L}^{2S}_{5}$ recorded from 220 to 350 nm: a) without metal addition, after addition of 1 equiv. b) Cu(II), c) Ni(II), d) Zn(II) and e) Co(II); f) superposition of a)-e).

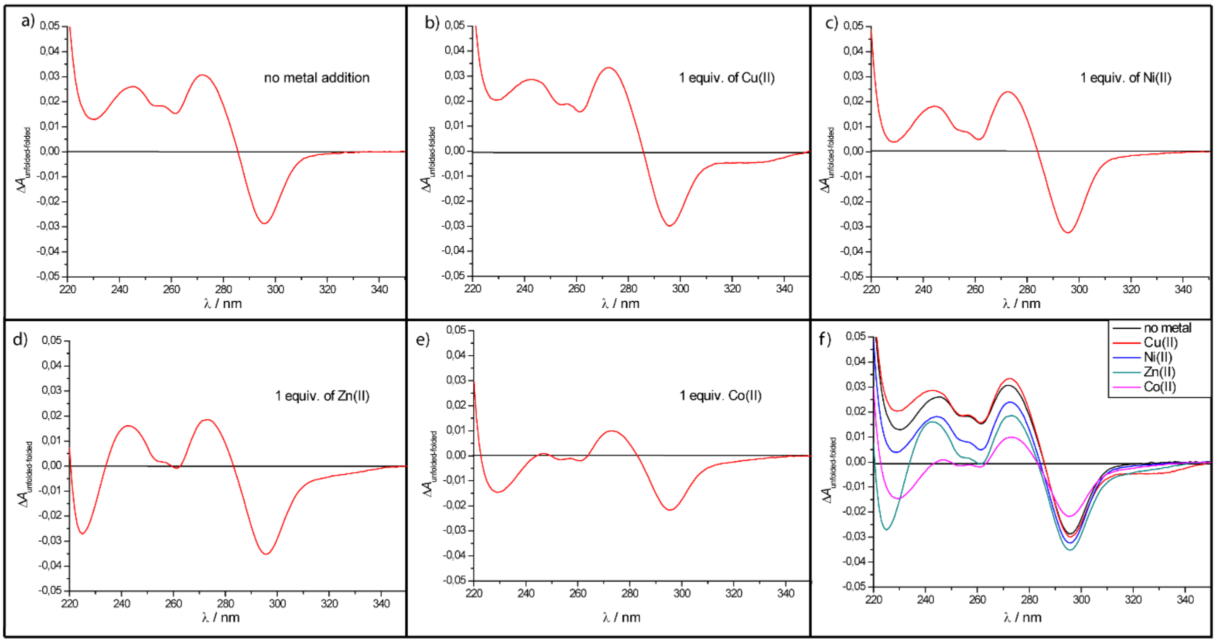

Figure 12. TDS of htel$\mathbf{L}^{2R}_{6}$ recorded from 220 to 350 nm: a) without metal addition, after addition of 1 equiv. b) Cu(II), c) Ni(II), d) Zn(II) and e) Co(II); f) superposition of a)-e).

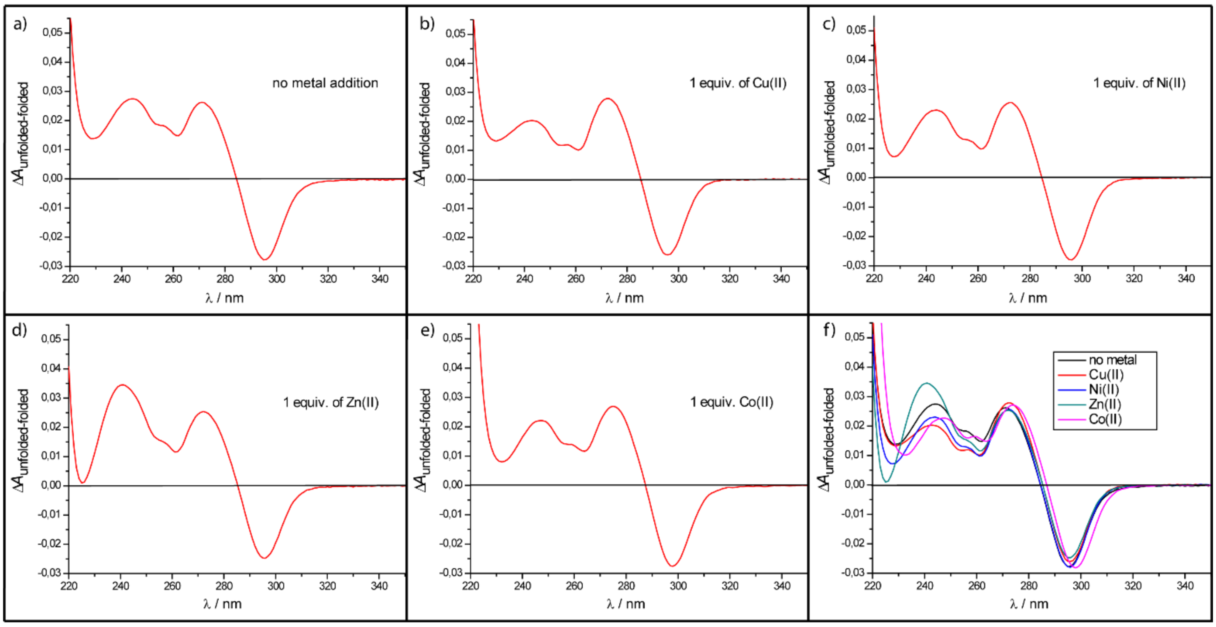

Figure 13. TDS of htel$\mathbf{L}^{2S}_{6}$ recorded from 220 to 350 nm: a) without metal addition, after addition of 1 equiv. b) Cu(II), c) Ni(II), d) Zn(II) and e) Co(II); f) superposition of a)-e).

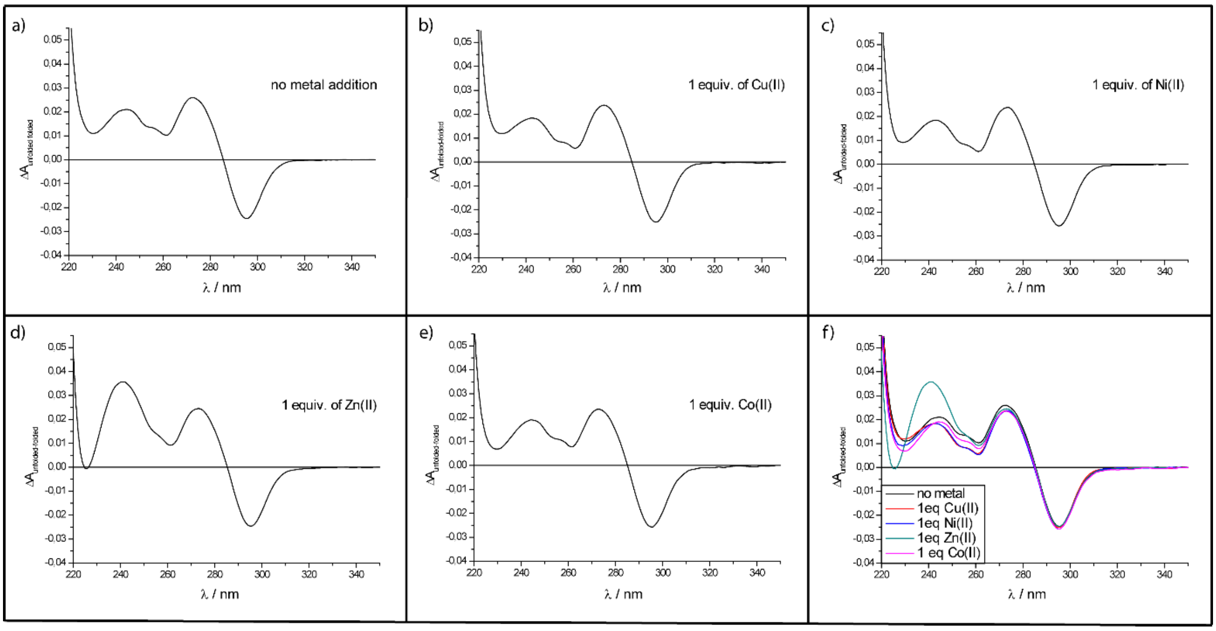

Figure 14. TDS of htel$\mathbf{L}^{2R}_{7}$ recorded from 220 to 350 nm: a) without metal addition, after addition of 1 equiv. b) Cu(II), c) Ni(II), d) Zn(II) and e) Co(II); f) superposition of a)-e).

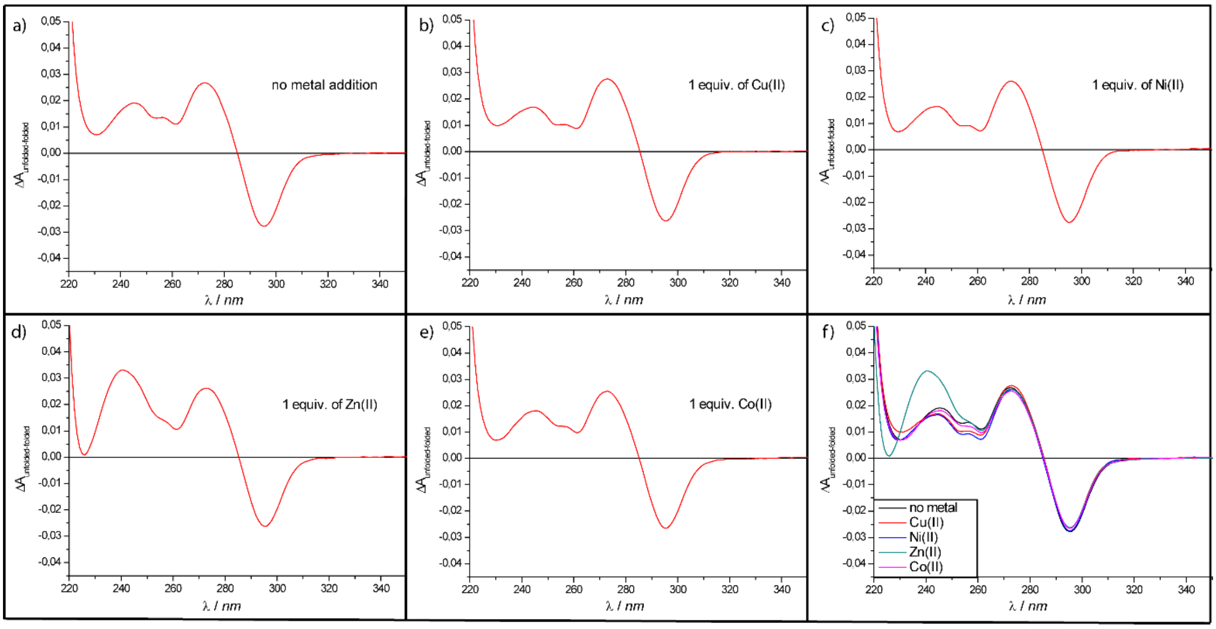

Figure 15. TDS of htel$\mathbf{L}^{2S}_7$ recorded from 220 to 350 nm: a) without metal addition, after addition of 1 equiv. b) Cu(II), c) Ni(II), d) Zn(II) and e) Co(II); f) superposition of a)-e).

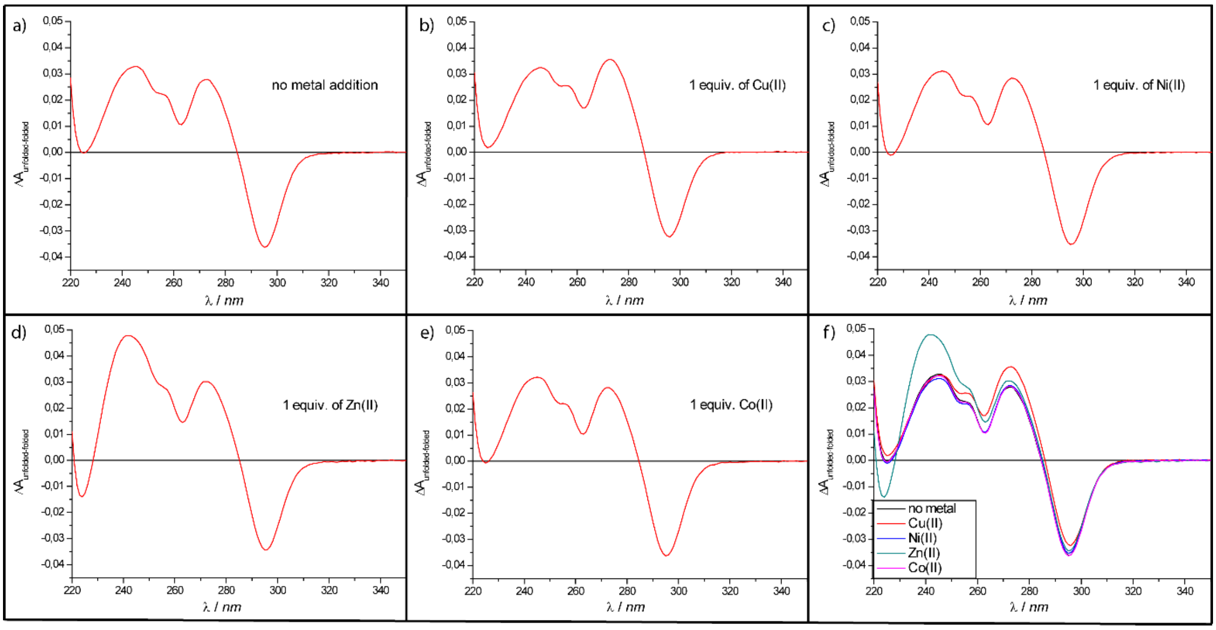

Figure 16. TDS of htel$\mathbf{L}^{2S}_2$B recorded from 220 to 350 nm: a) without metal addition, after addition of 1 equiv. b) Cu(II), c) Ni(II), d) Zn(II) and e) Co(II); f) superposition of a)-e).

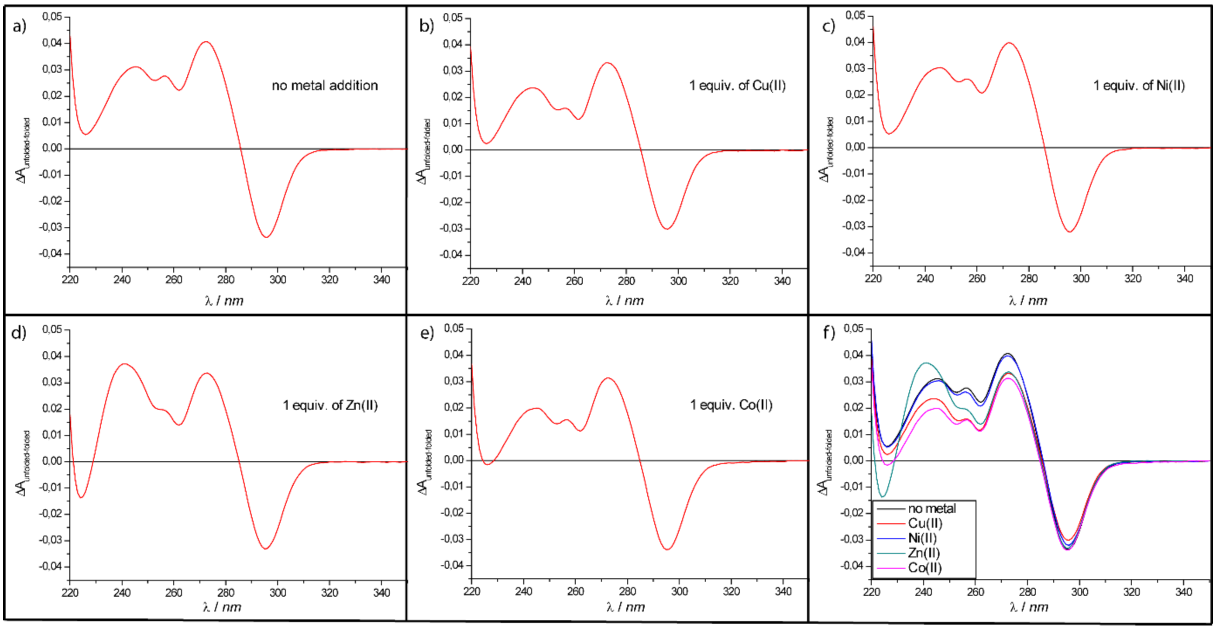

Figure 17. TDS of htel**L**$^{2S}{}_{3}$B recorded from 220 to 350 nm: a) without metal addition, after addition of 1 equiv. b) Cu(II), c) Ni(II), d) Zn(II) and e) Co(II); f) superposition of a)-e).

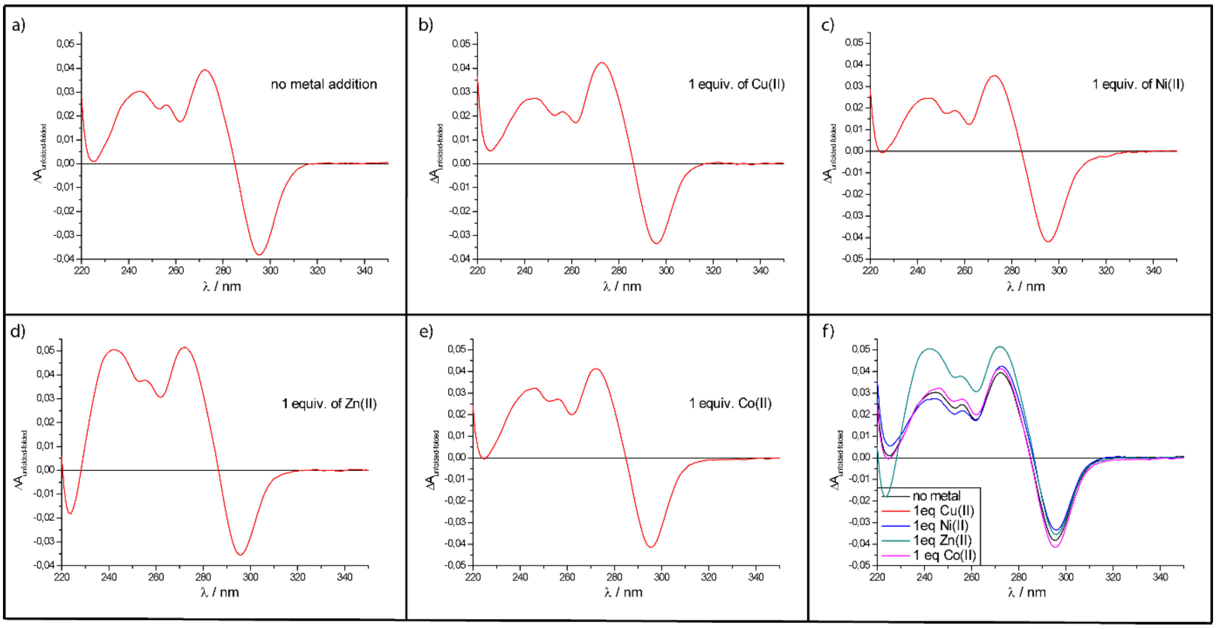

Figure 18. TDS of htel**L**$^{2R}{}_{2}$B recorded from 220 to 350 nm: a) without metal addition, after addition of 1 equiv. b) Cu(II), c) Ni(II), d) Zn(II) and e) Co(II); f) superposition of a)-e).

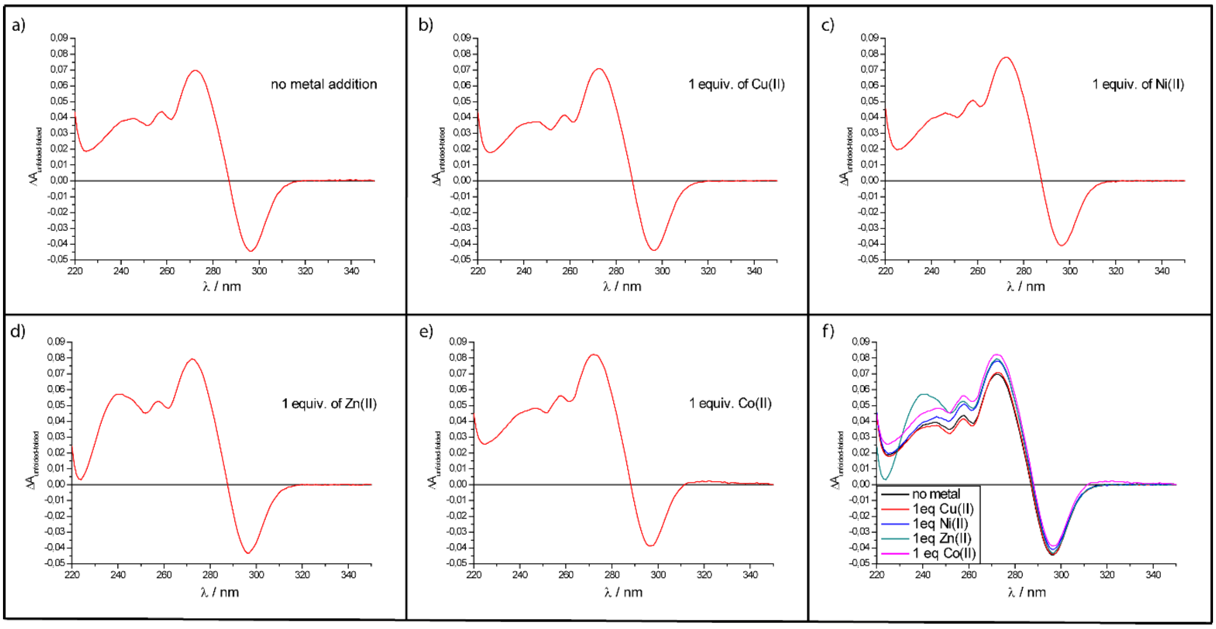

Figure 19. TDS of htel$\mathbf{L}^{2R}_3$B recorded from 220 to 350 nm: a) without metal addition, after addition of 1 equiv. b) Cu(II), c) Ni(II), d) Zn(II) and e) Co(II); f) superposition of a)-e).

4 Thermal denaturation experiments

Table **3**. Melting temperatures T_m and stabilizations (ΔT_m) in absence and after addition of Cu(II), Zn(II), Ni(II) and Co(II)

	No metal	Cu(II)	Ni(II)	Zn(II)	Co(II)
$G_4\mathbf{L}^{1S}$	31	79 (+48)	77 (+46)	54 (+23)	64 (+33)
$G_4\mathbf{L}^{1R}$	32	83 (+51)	81 (+49)	61 (+29)	73 (+41)
$G_4\mathbf{L}^{2S}$	36	76 (+40)	73 (+37)	52 (+16)	63 (+27)
$G_4\mathbf{L}^{2R}$	34	74 (+40)	68 (+34)	46 (+12)	54 (+20)
htel$\mathbf{L}^{1S}_4$	37	36 (-1)	36 (-1)	36 (-1)	37 (+0)
htel$\mathbf{L}^{1R}_4$	29	29 (+0)	28 (-1)	29 (+0)	29 (+0)
htel$\mathbf{L}^{2S}_2$	27	28 (+1)	27 (+0)	27 (+0)	26 (-1)
htel$\mathbf{L}^{2S}_2$B in Na	53	55 (+2)	53 (+0)	54 (+1)	53 (+0)
htel$\mathbf{L}^{2R}_2$	29	29 (+0)	29 (+0)	29 (+0)	28 (-1)
htel$\mathbf{L}^{2R}_2$B in Na	52	52 (+0)	49 (-3)	52 (+0)	52 (+0)
htel$\mathbf{L}^{2S}_3$	28	31 (+3)	28 (+0)	28 (+0)	27 (-1)
htel$\mathbf{L}^{2S}_3$B in Na	50	56 (+6)	51 (+1)	52 (+2)	50 (+0)
htel$\mathbf{L}^{2R}_3$	29	31 (+2)	28 (-1)	29 (+0)	30 (+1)
htel$\mathbf{L}^{2R}_3$B in Na	45	49 (+4)	46 (+1)	49 (+4)	46 (+1)
htel$\mathbf{L}^{2S}_4$	33	56 (+23)	45 (+12)	36 (+3)	35 (+2)
htel$\mathbf{L}^{2S}_4$ 2 equiv. Metal	33	53 (+20)	45 (+12)	35 (+2)	35 (+2)
htel$\mathbf{L}^{2S}_4$B	37	60 (+23)	n.d.	56 (+19)	n.d.
htel$\mathbf{L}^{2S}_4$C	42	63 (+21)	n.d.	63 (+21)	n.d.
htel$\mathbf{L}^{2R}_4$	34	56 (+22)	45 (+11)	36 (+2)	36 (+2)
htel$\mathbf{L}_4^{2R}$ 2 equiv. Metal	34	51 (+17)	45 (+11)	37 (+3)	32 (-2)
htel$\mathbf{L}^{2R}_4$B	36	62 (+26)	n.d.	52 (+16)	n.d.

htel**L**$^{2R}{}_{4}$C	43	66 (+23)	n.d.	55 (+12)	n.d.
htel**L**$^{2S}{}_{5}$	33	54 (+21)	55 (+22)	37 (+4)	37 (+4)
htel**L**$^{2S}{}_{5}$ 2 equiv. Metal	33	46 (+13)	55 (+22)	38 (+5)	36 (+3)
htel**L**$^{2R}{}_{5}$	34	50 (+16)	47 (+13)	36 (+2)	35 (+1)
htel**L**$^{2R}{}_{5}$ 2 equiv. Metal	34	47 (+13)	46 (+12)	37 (+3)	36 (+2)
htel**L**$^{2S}{}_{6}$	36	54 (+18)	59 (+23)	44 (+8)	44 (+8)
htel**L**$^{2S}{}_{6}$ 2 equiv. Metal	34	52 (+ 18)	52 (+ 18)	42 (+ 8)	36 (+ 2)
htel**L**$^{2R}{}_{6}$	35	50 (+15)	51 (+16)	42 (+7)	41 (+6)
htel**L**$^{2R}{}_{6}$ 2 equiv. Metal	35	51 (+16)	50 (+15)	44 (+9)	43 (+8)
htel**L**$^{2S}{}_{7}$	28	43 (+15)	46 (+18)	36 (+8)	36 (+8)
htel**L**$^{2S}{}_{7}$ 2 equiv. Metal	28	46 (+18)	46 (+18)	38 (+10)	39 (+11)
htel**L**$^{2R}{}_{7}$	29	43 (+14)	44 (+15)	36 (+7)	30 (+1)
htel**L**$^{2R}{}_{7}$ 2 equiv. Metal	29	46 (+17)	44 (+15)	39 (+10)	32 (+3)

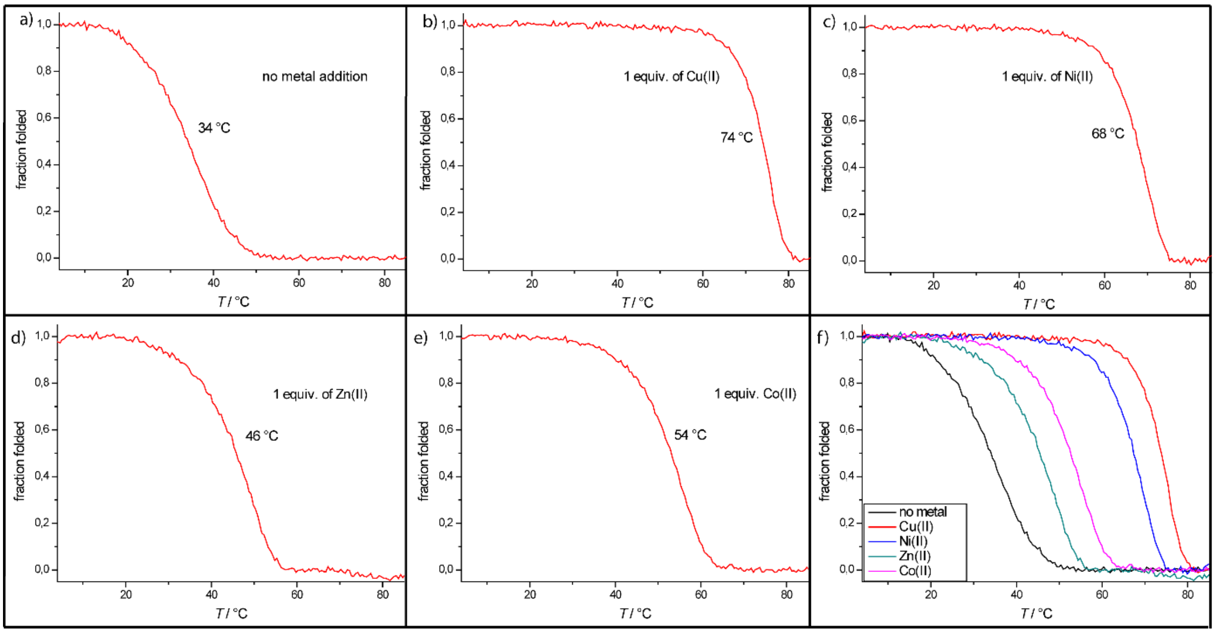

Figure 20. Thermal denaturation experiments of G_4**L**^{2R} monitored at λ = 295 nm and converted to fraction folded a) in absence of transition metals and after addition of 1 equiv. b) Cu(II), c) Ni(II), d) Zn(II) and e) Co(II); f) superposition of a)-e).

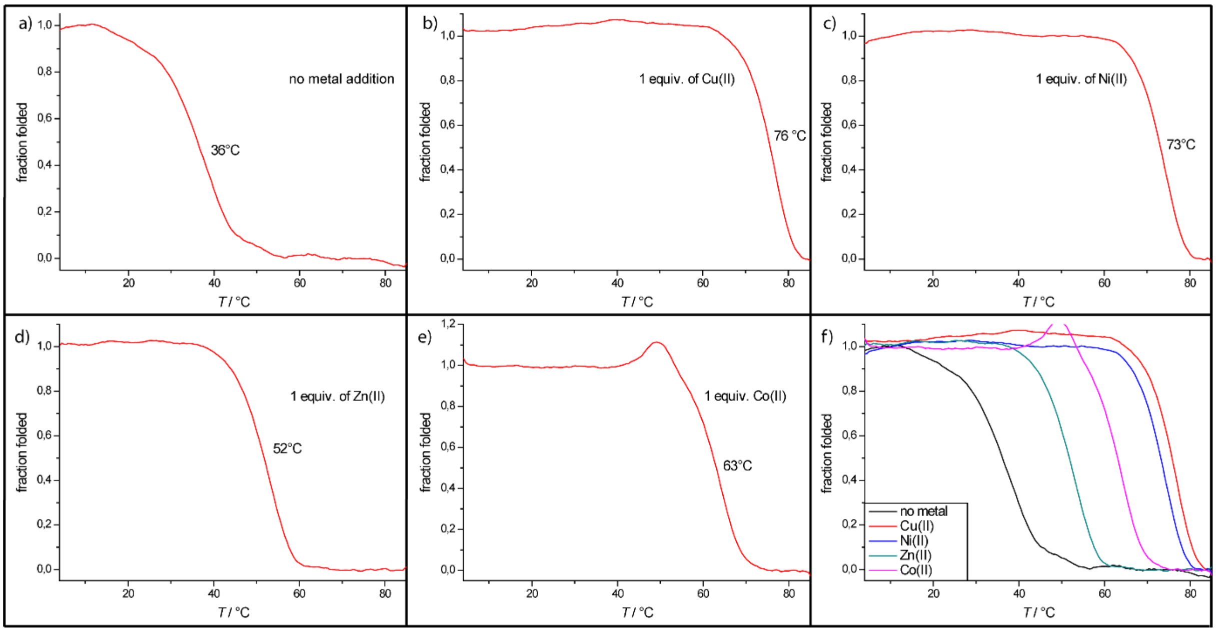

Figure 21. Thermal denaturation experiments of $G_4\mathbf{L}^{2S}$ monitored at λ = 295 nm and converted to fraction folded a) in absence of transition metals and after addition of 1 equiv. b) Cu(II), c) Ni(II), d) Zn(II) and e) Co(II); f) superposition of a)-e).

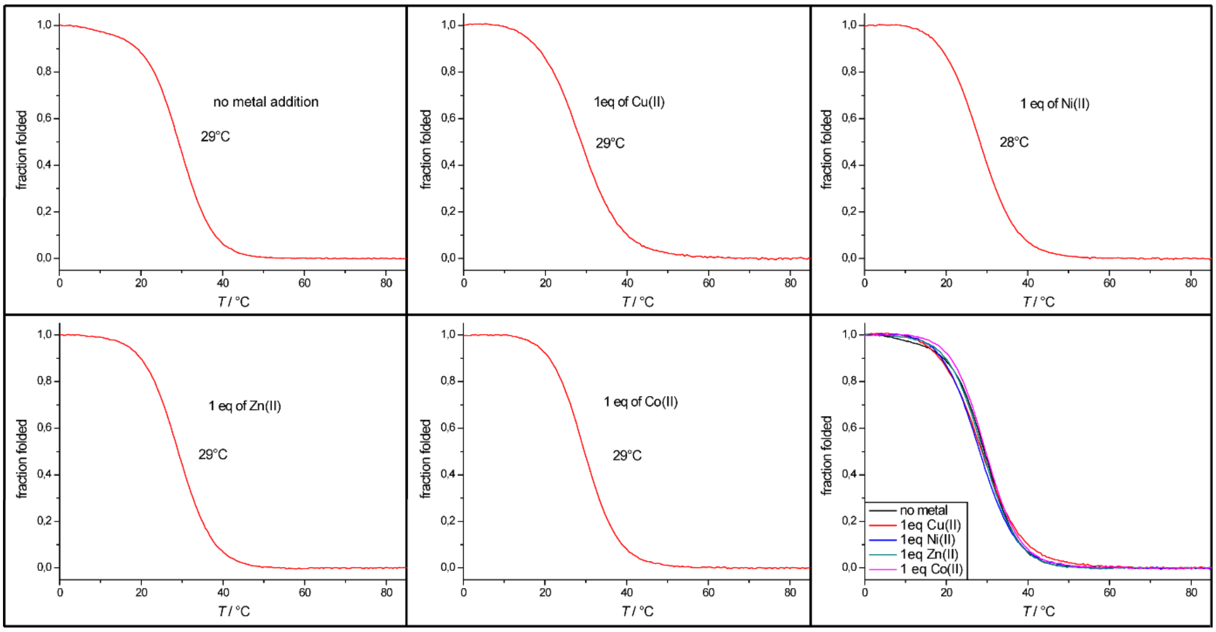

Figure 22. Thermal denaturation experiments of htel$\mathbf{L}^{1R}_4$ monitored at λ = 295 nm and converted to fraction folded a) in absence of transition metals and after addition of 1 equiv. b) Cu(II), c) Ni(II), d) Zn(II) and e) Co(II); f) superposition of a)-e).

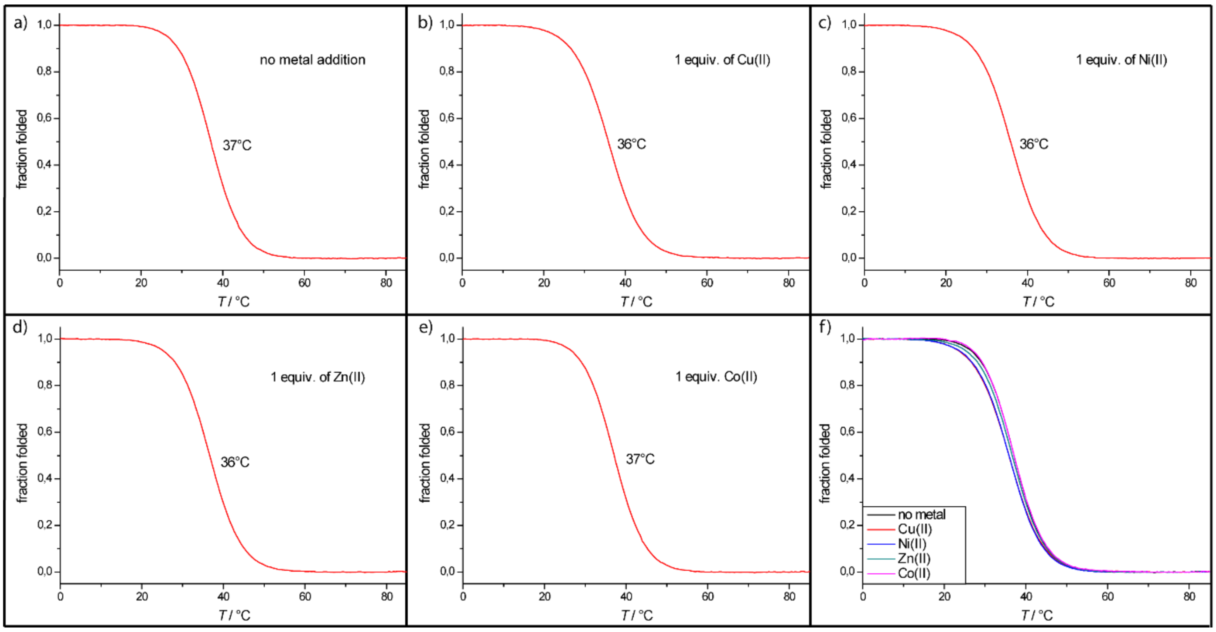

Figure 23. Thermal denaturation experiments of htel**L**$^{1S}_{4}$ monitored at λ = 295 nm and converted to fraction folded a) in absence of transition metals and after addition of 1 equiv. b) Cu(II), c) Ni(II), d) Zn(II) and e) Co(II); f) superposition of a)-e).

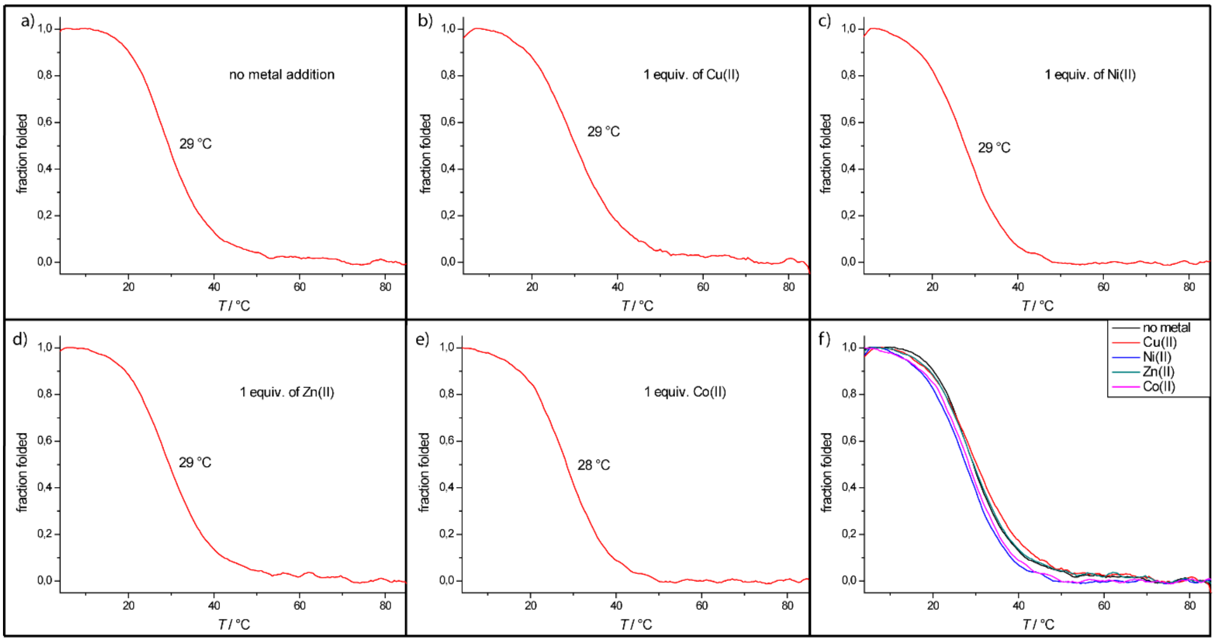

Figure 24. Thermal denaturation experiments of htel**L**$^{2R}_{2}$ monitored at λ = 295 nm and converted to fraction folded a) in absence of transition metals and after addition of 1 equiv. b) Cu(II), c) Ni(II), d) Zn(II) and e) Co(II); f) superposition of a)-e).

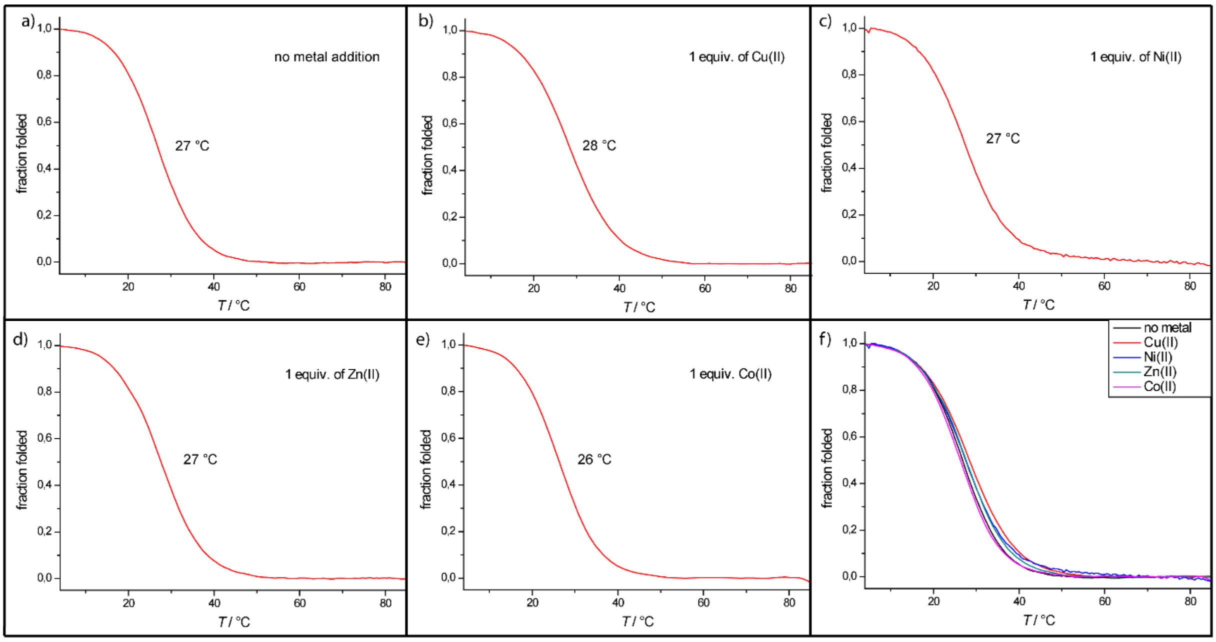

Figure 25. Thermal denaturation experiments of htel**L**$^{2S}{}_2$ monitored at λ = 295 nm and converted to fraction folded a) in absence of transition metals and after addition of 1 equiv. b) Cu(II), c) Ni(II), d) Zn(II) and e) Co(II); f) superposition of a)-e).

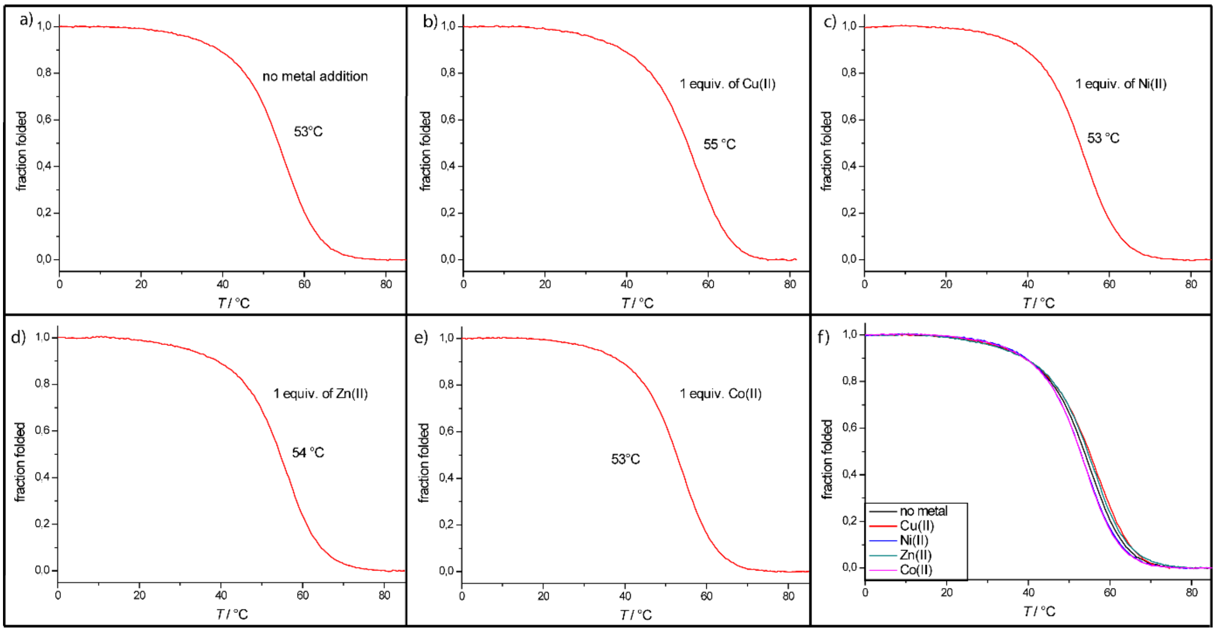

Figure 26. Thermal denaturation experiments of htel**L**$^{2S}{}_2$B in NaCl monitored at λ = 295 nm and converted to fraction folded a) in absence of transition metals and after addition of 1 equiv. b) Cu(II), c) Ni(II), d) Zn(II) and e) Co(II); f) superposition of a)-e).

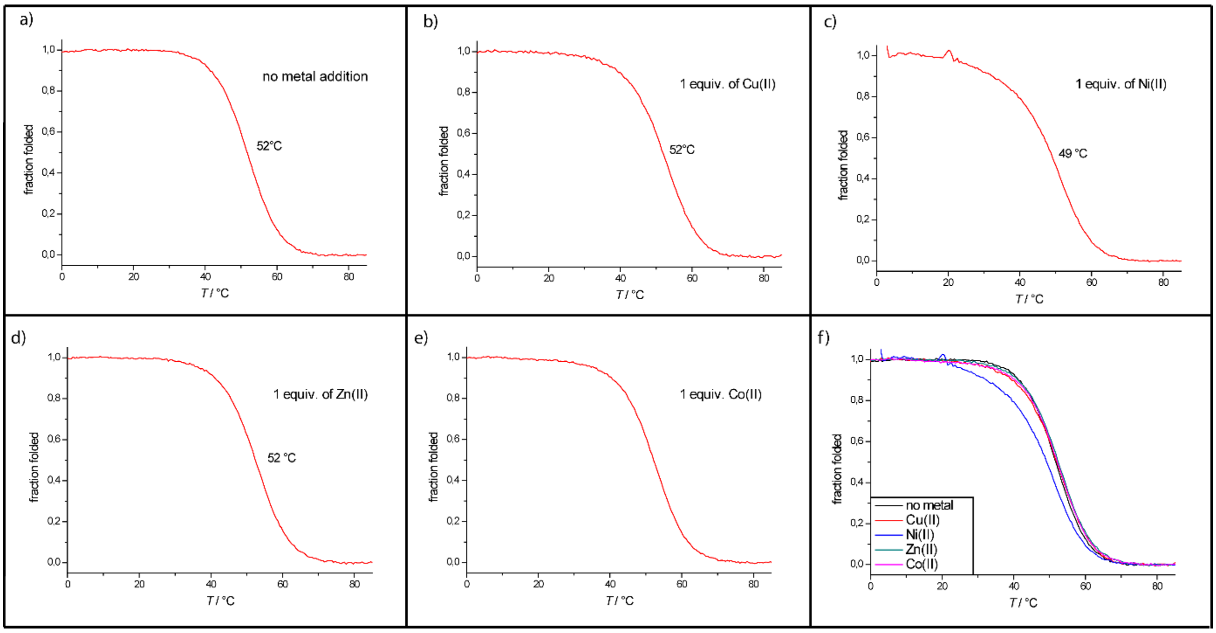

Figure 27. Thermal denaturation experiments of htel$\mathbf{L}^{2R}{}_{2}$B in NaCl monitored at λ = 295 nm and converted to fraction folded a) in absence of transition metals and after addition of 1 equiv. b) Cu(II), c) Ni(II), d) Zn(II) and e) Co(II); f) superposition of a)-e).

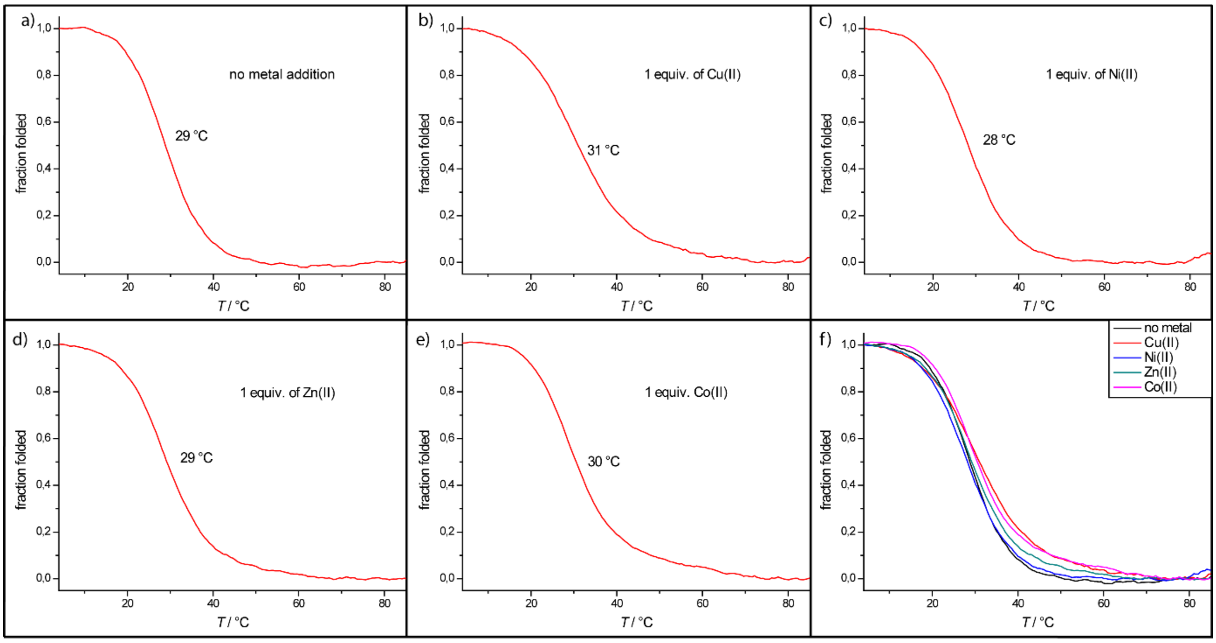

Figure 28. Thermal denaturation experiments of htel$\mathbf{L}^{2R}{}_{3}$ monitored at λ = 295 nm and converted to fraction folded a) in absence of transition metals and after addition of 1 equiv. b) Cu(II), c) Ni(II), d) Zn(II) and e) Co(II); f) superposition of a)-e).

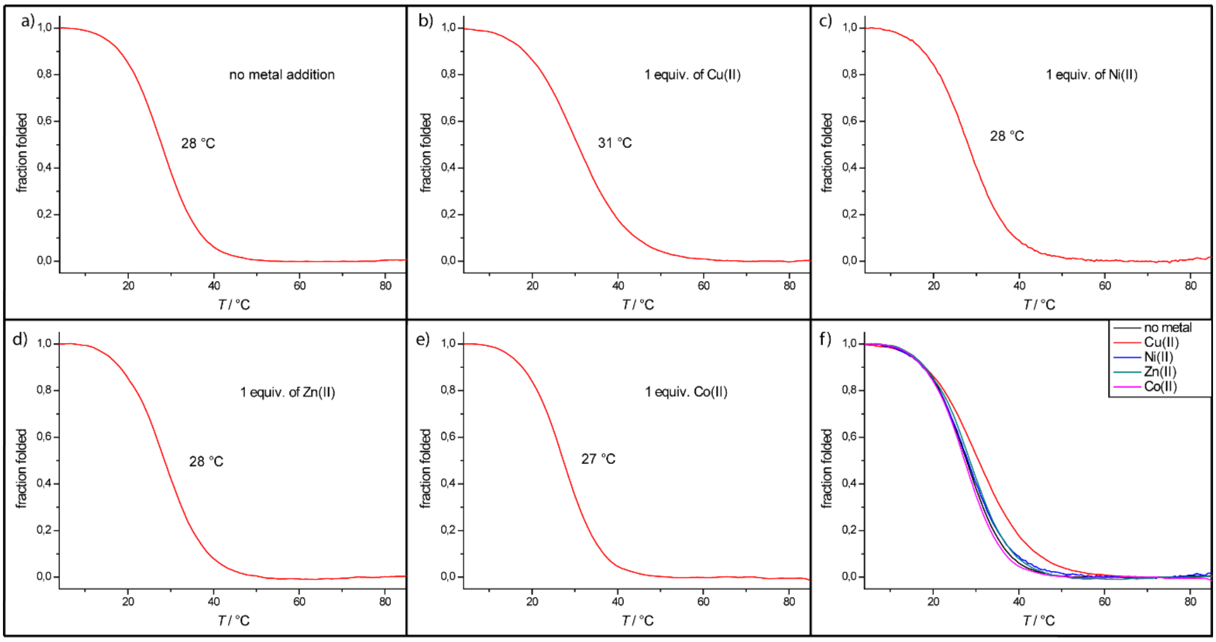

Figure 29. Thermal denaturation experiments of htel**L**$^{2S}{}_{3}$ monitored at λ = 295 nm and converted to fraction folded a) in absence of transition metals and after addition of 1 equiv. b) Cu(II), c) Ni(II), d) Zn(II) and e) Co(II); f) superposition of a)-e).

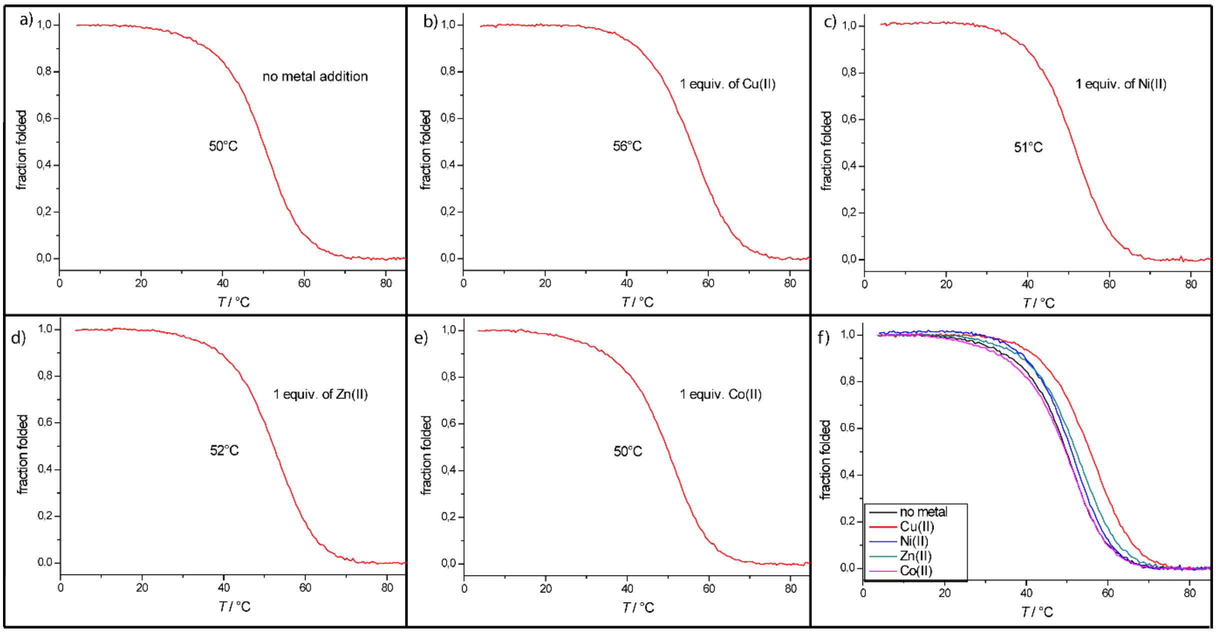

Figure 30. Thermal denaturation experiments of htel**L**$^{2S}{}_{3}$B in NaCl monitored at λ = 295 nm and converted to fraction folded a) in absence of transition metals and after addition of 1 equiv. b) Cu(II), c) Ni(II), d) Zn(II) and e) Co(II); f) superposition of a)-e).

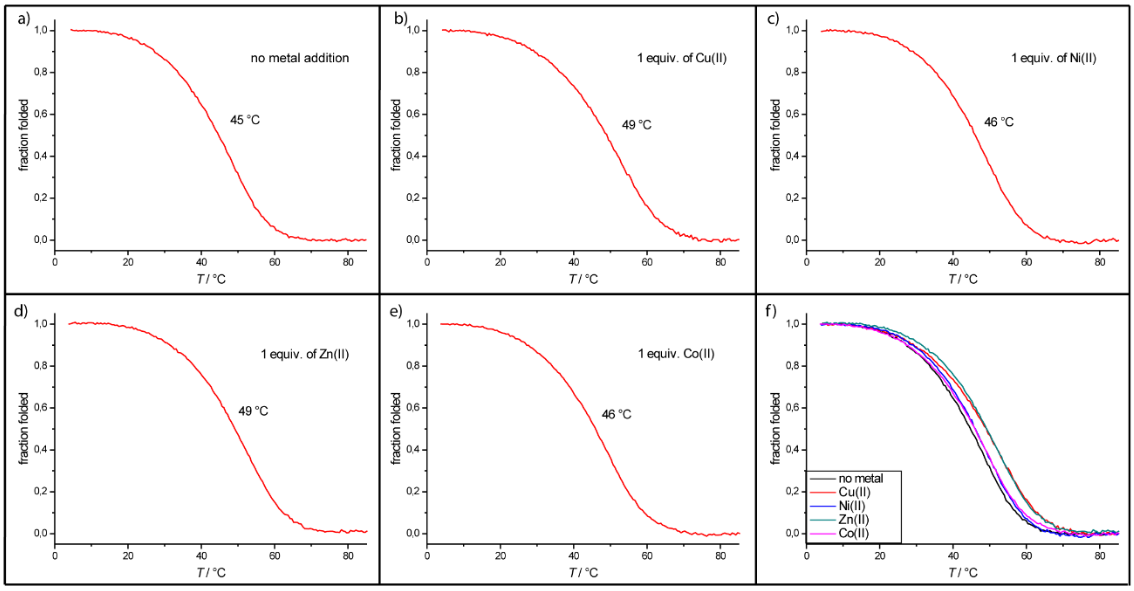

Figure 31. Thermal denaturation experiments of htel$\mathbf{L}^{2R}_3$B in NaCl monitored at λ = 295 nm and converted to fraction folded a) in absence of transition metals and after addition of 1 equiv. b) Cu(II), c) Ni(II), d) Zn(II) and e) Co(II); f) superposition of a)-e).

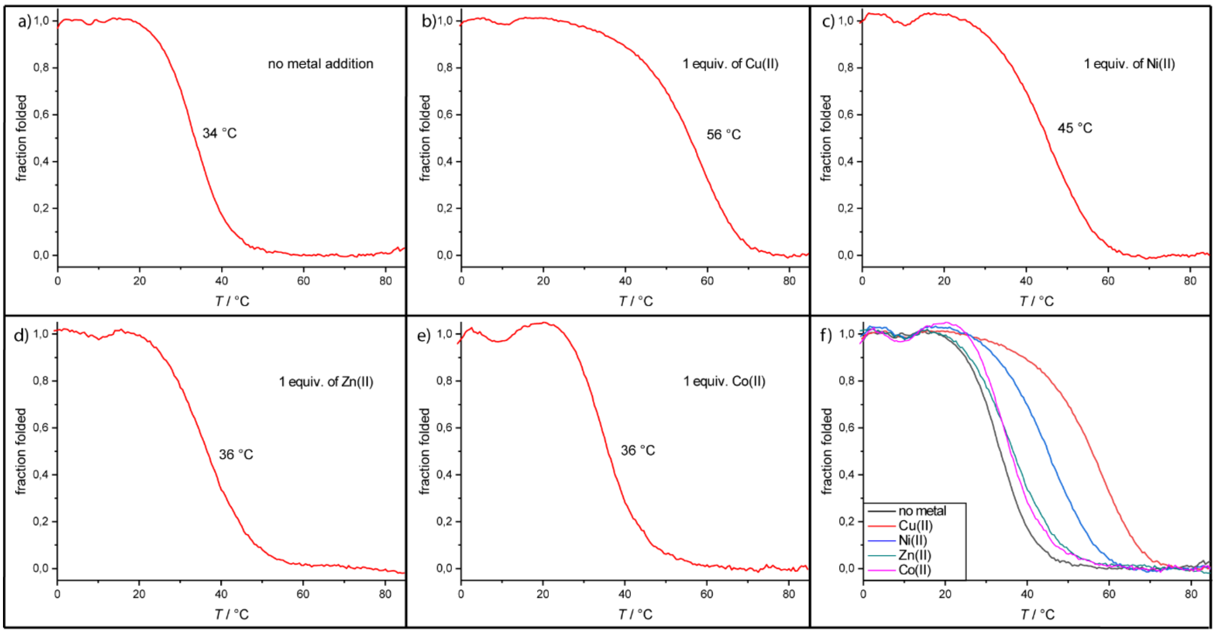

Figure 32. Thermal denaturation experiments of htel$\mathbf{L}^{2R}_4$ monitored at λ = 295 nm and converted to fraction folded a) in absence of transition metals and after addition of 1 equiv. b) Cu(II), c) Ni(II), d) Zn(II) and e) Co(II); f) superposition of a)-e).

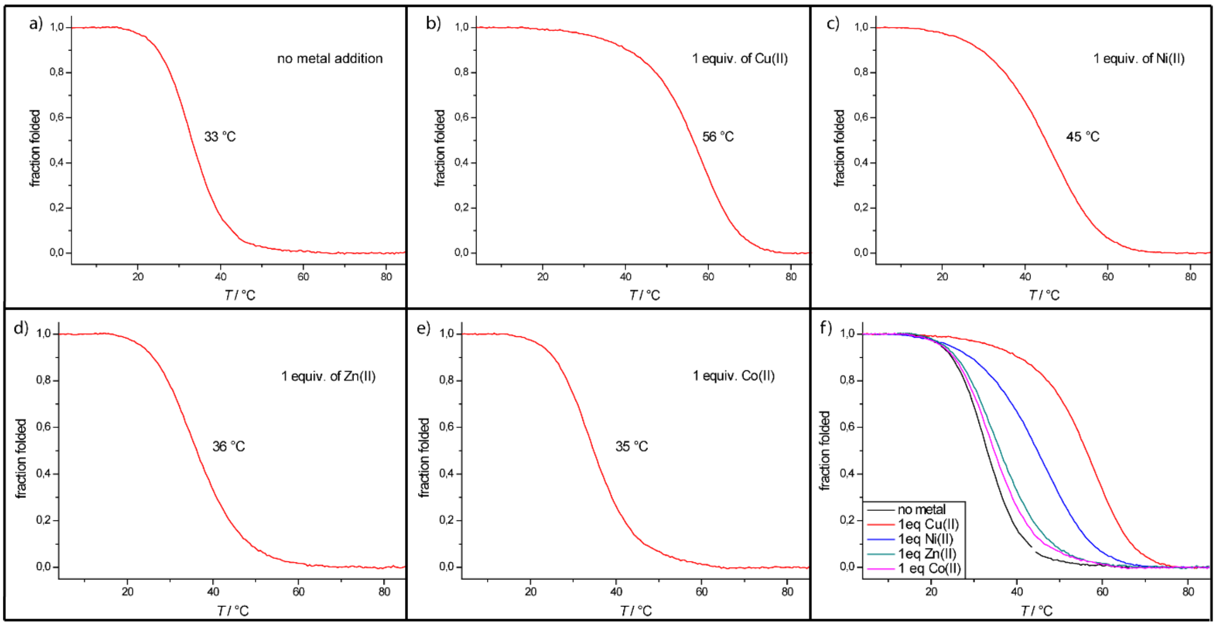

Figure 33. Thermal denaturation experiments of htel$\mathbf{L}^{2S}_4$ monitored at λ = 295 nm and converted to fraction folded a) in absence of transition metals and after addition of 1 equiv. b) Cu(II), c) Ni(II), d) Zn(II) and e) Co(II); f) superposition of a)-e).

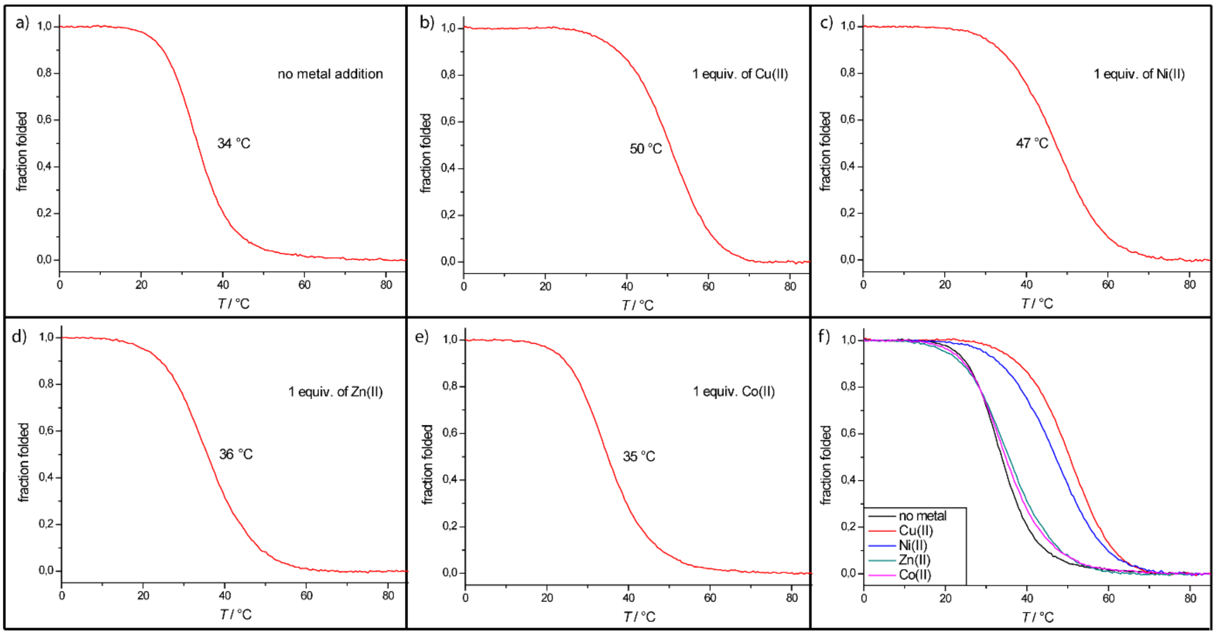

Figure 34. Thermal denaturation experiments of htel$\mathbf{L}^{2R}_5$ monitored at λ = 295 nm and converted to fraction folded a) in absence of transition metals and after addition of 1 equiv. b) Cu(II), c) Ni(II), d) Zn(II) and e) Co(II); f) superposition of a)-e).

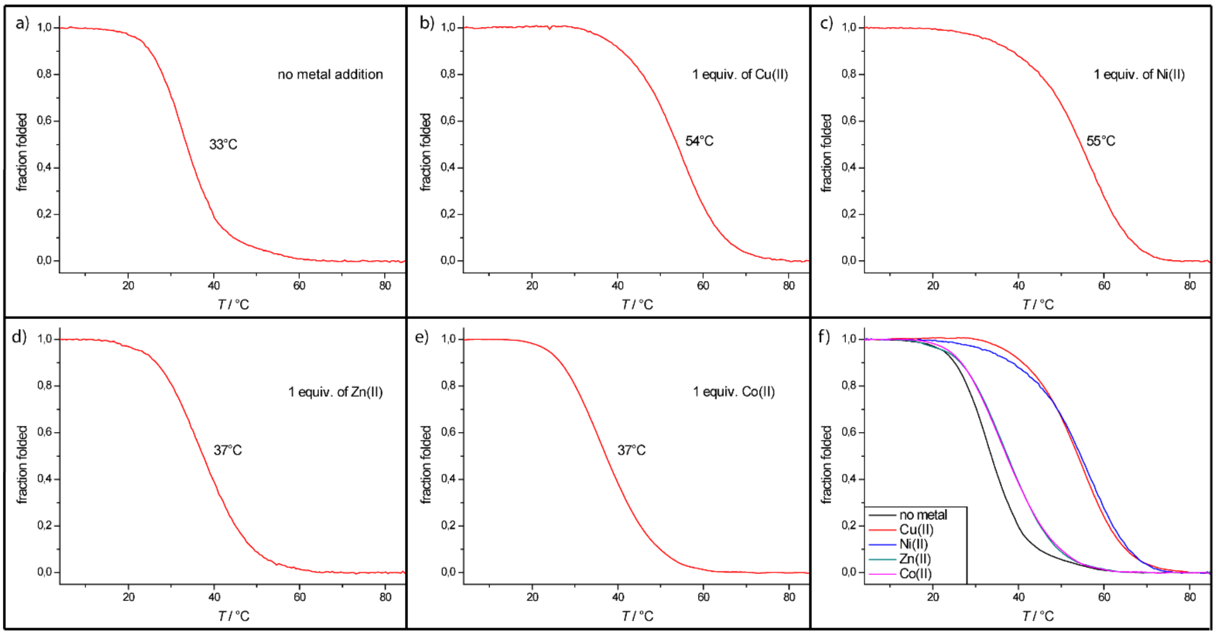

Figure 35. Thermal denaturation experiments of htel$\mathbf{L}^{2S}_5$ monitored at λ = 295 nm and converted to fraction folded a) in absence of transition metals and after addition of 1 equiv. b) Cu(II), c) Ni(II), d) Zn(II) and e) Co(II); f) superposition of a)-e).

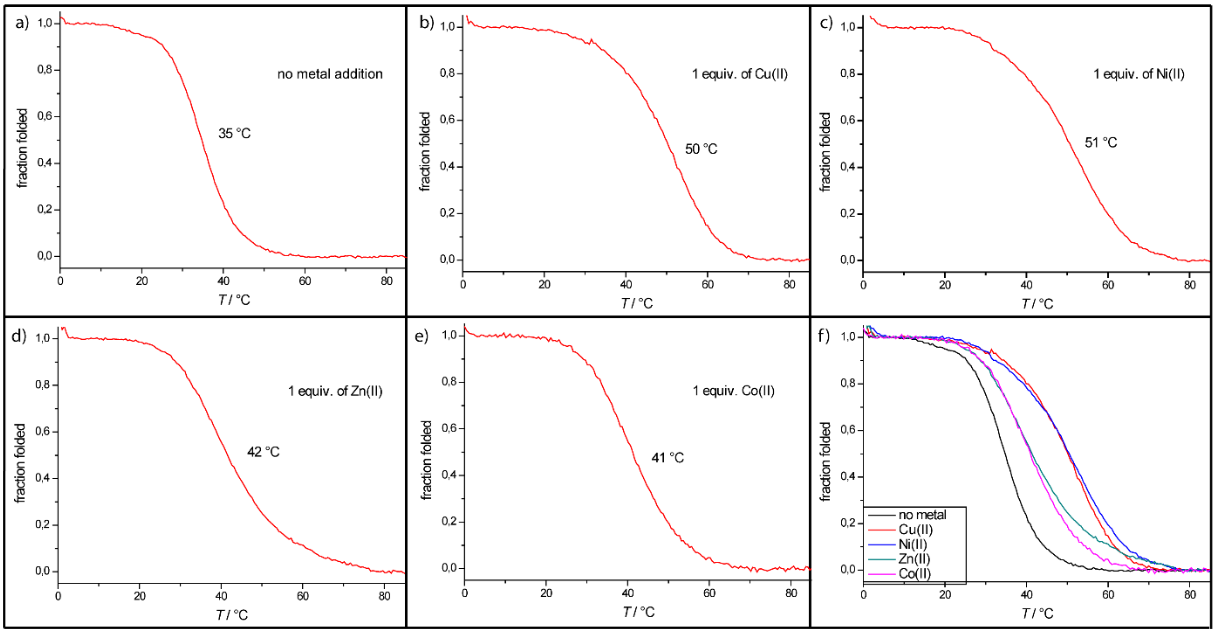

Figure 36. Thermal denaturation experiments of htel$\mathbf{L}^{2R}_{6}$ monitored at λ = 295 nm and converted to fraction folded a) in absence of transition metals and after addition of 1 equiv. b) Cu(II), c) Ni(II), d) Zn(II) and e) Co(II); f) superposition of a)-e).

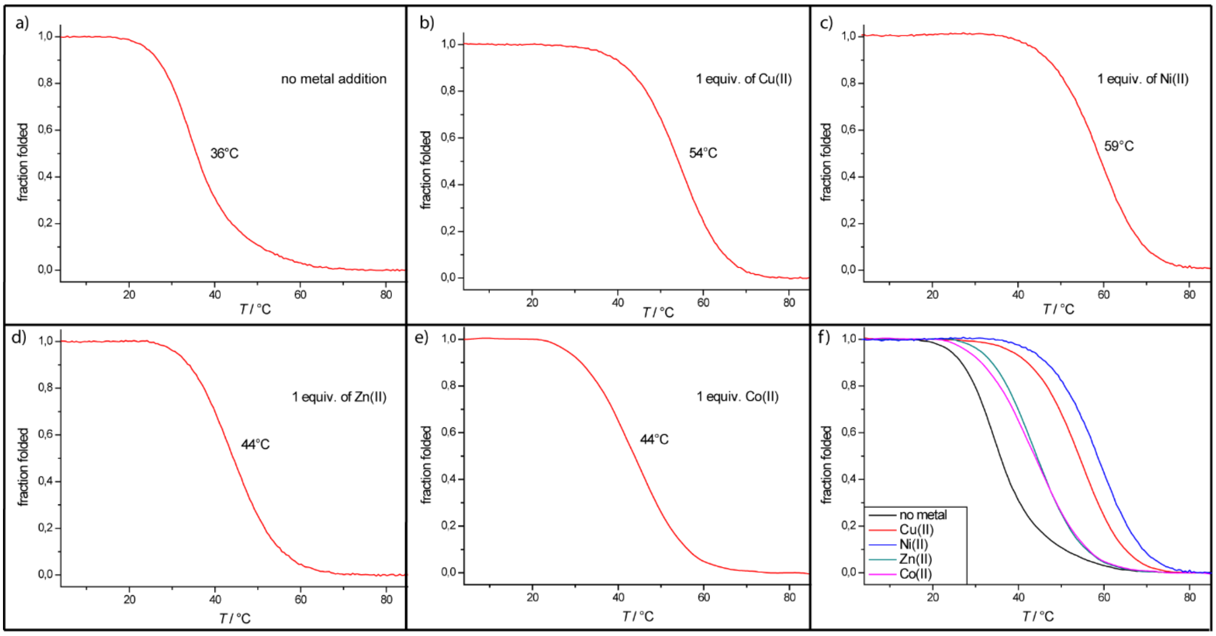

Figure 37. Thermal denaturation experiments of htel$\mathbf{L}^{2S}_{6}$ monitored at λ = 295 nm and converted to fraction folded a) in absence of transition metals and after addition of 1 equiv. b) Cu(II), c) Ni(II), d) Zn(II) and e) Co(II); f) superposition of a)-e).

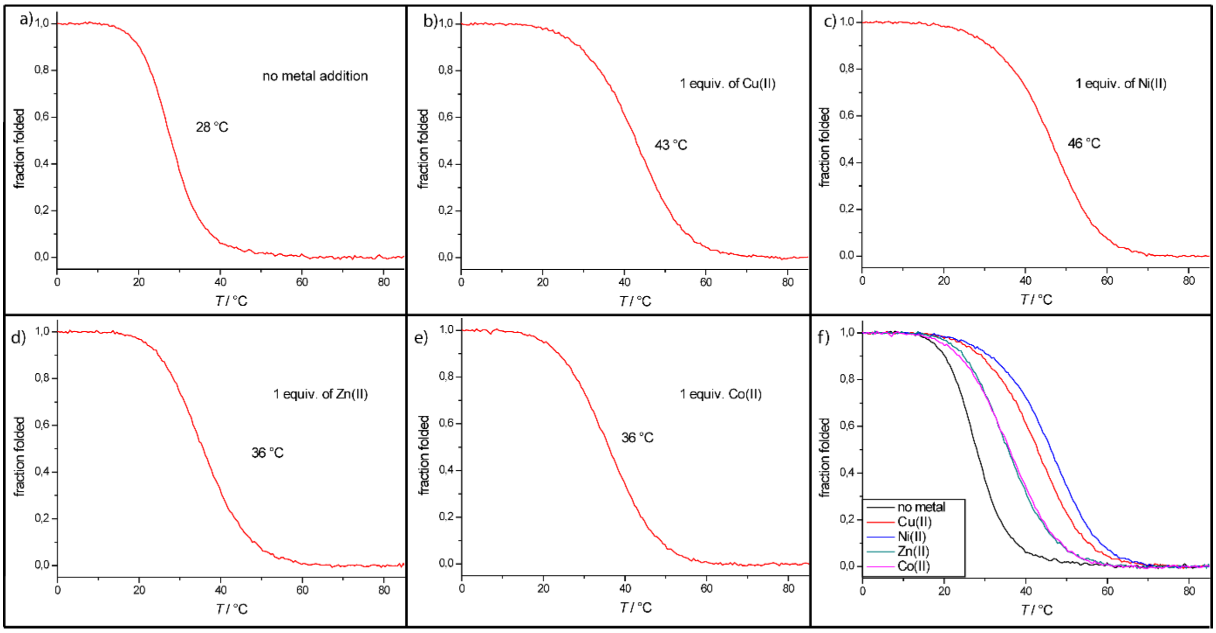

Figure 38. Thermal denaturation experiments of htel$\mathbf{L}^{2S}_{7}$ monitored at λ = 295 nm and converted to fraction folded a) in absence of transition metals and after addition of 1 equiv. b) Cu(II), c) Ni(II), d) Zn(II) and e) Co(II); f) superposition of a)-e).

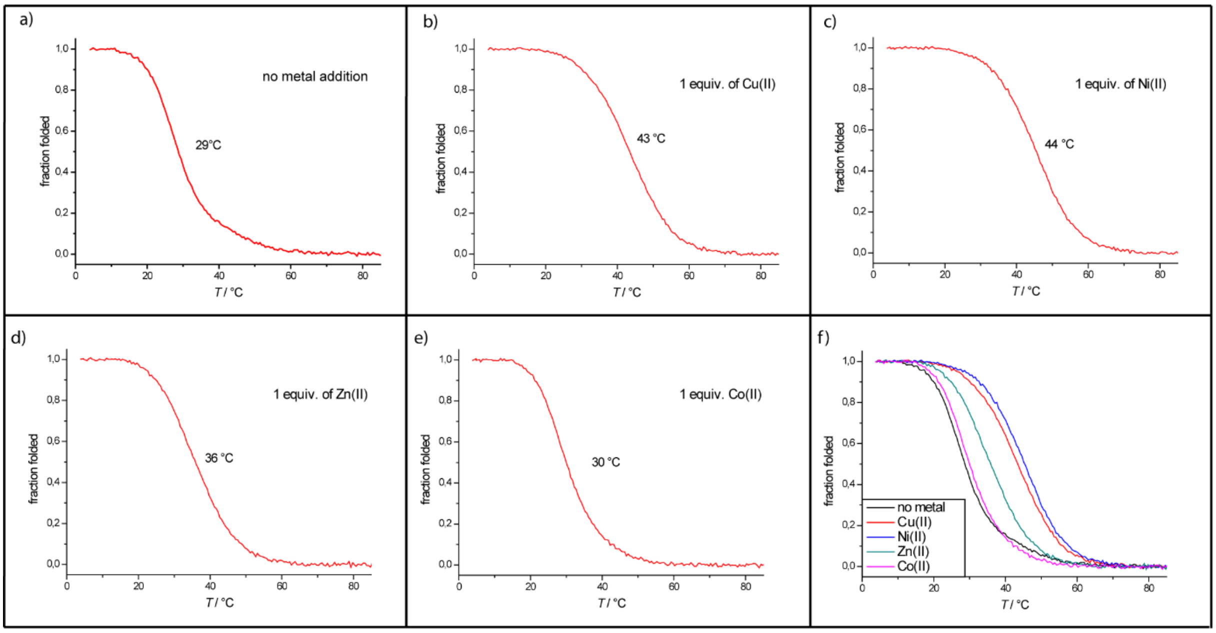

Figure 39. Thermal denaturation experiments of htel$\mathbf{L}^{2R}{}_{7}$ monitored at λ = 295 nm and converted to fraction folded a) in absence of transition metals and after addition of 1 equiv. b) Cu(II), c) Ni(II), d) Zn(II) and e) Co(II); f) superposition of a)-e).

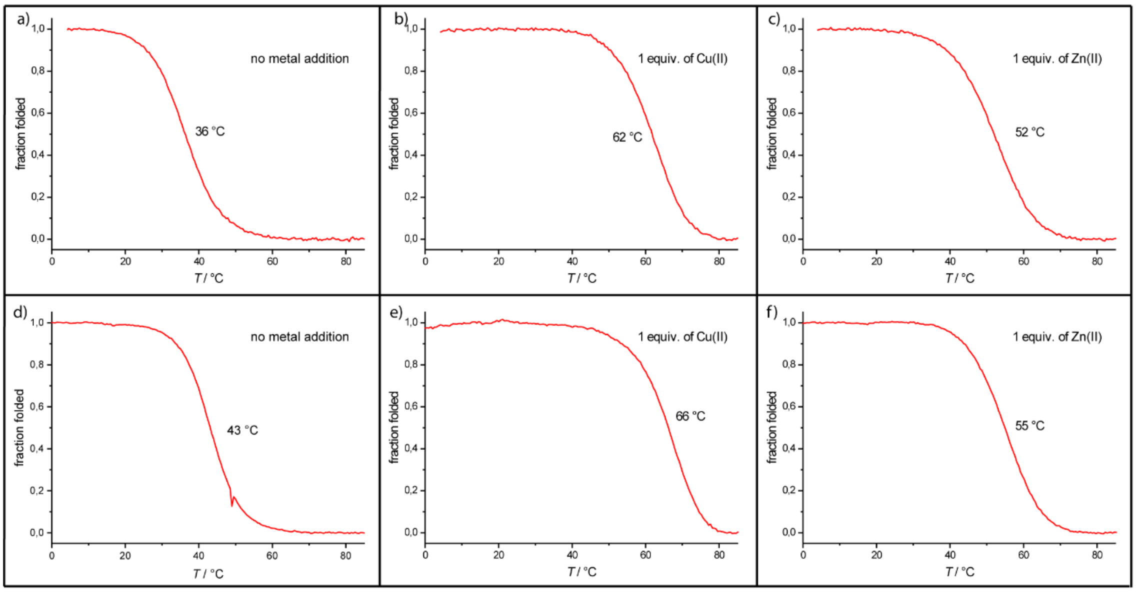

Figure 40. Thermal denaturation experiments of htel$\mathbf{L}^{2R}{}_{4}$B and htel$\mathbf{L}^{2R}{}_{4}$C monitored at λ = 295 nm and converted to fraction folded: a) htel$\mathbf{L}^{2R}{}_{4}$B without transition metal and with 1 equiv. b) Cu(II), c) Zn(II). d) htel$\mathbf{L}^{2R}{}_{4}$C without transition metal and with 1 equiv. e) Cu(II), f) Zn(II).

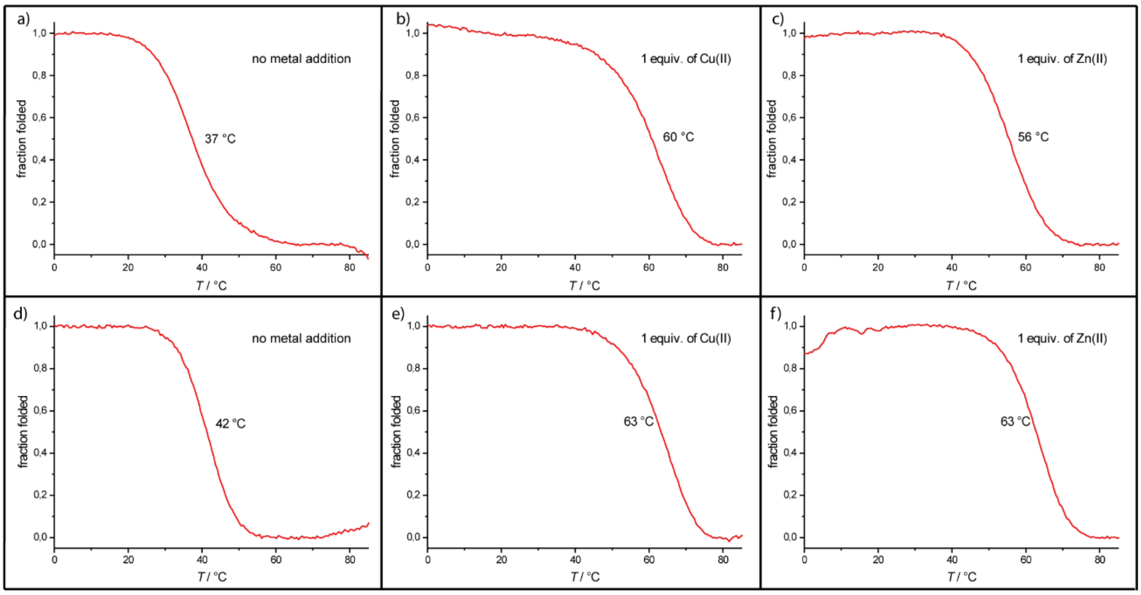

Figure 41. Thermal denaturation experiments of htel$\mathbf{L}^{2S}{}_4$B and htel$\mathbf{L}^{2S}{}_4$C monitored at λ = 295 nm and converted to fraction folded: a) htel$\mathbf{L}^{2S}{}_4$B without transition metal and with 1 equiv. b) Cu(II), c) Zn(II). d) htel$\mathbf{L}^{2S}{}_4$C without transition metal and with 1 equiv. e) Cu(II), f) Zn(II).

5 Circular dichroism (CD) measurements

CD measurements were carried out on a *Chirascan qCD spectrometer* in black quartz cuvettes. Samples were prepared as described under **UV/VIS spectroscopy**. Temperature was controlled using a *Quantum Northwest temperature control* attached to a sample probe. Spectra were recorded from 205 to 350 nm (120 nm min^{-1}) with a 1 nm interval and 0.5 nm bandwidth three times and averaged using the built-in software. The averaged spectra were smoothed (adjacent averaging) with a factor of 5 and the smoothed background (sample containing only buffer, smooth factor 10) was subtracted. The background was recorded once from 205 to 350 nm with the same settings as for the samples in the same cuvette.

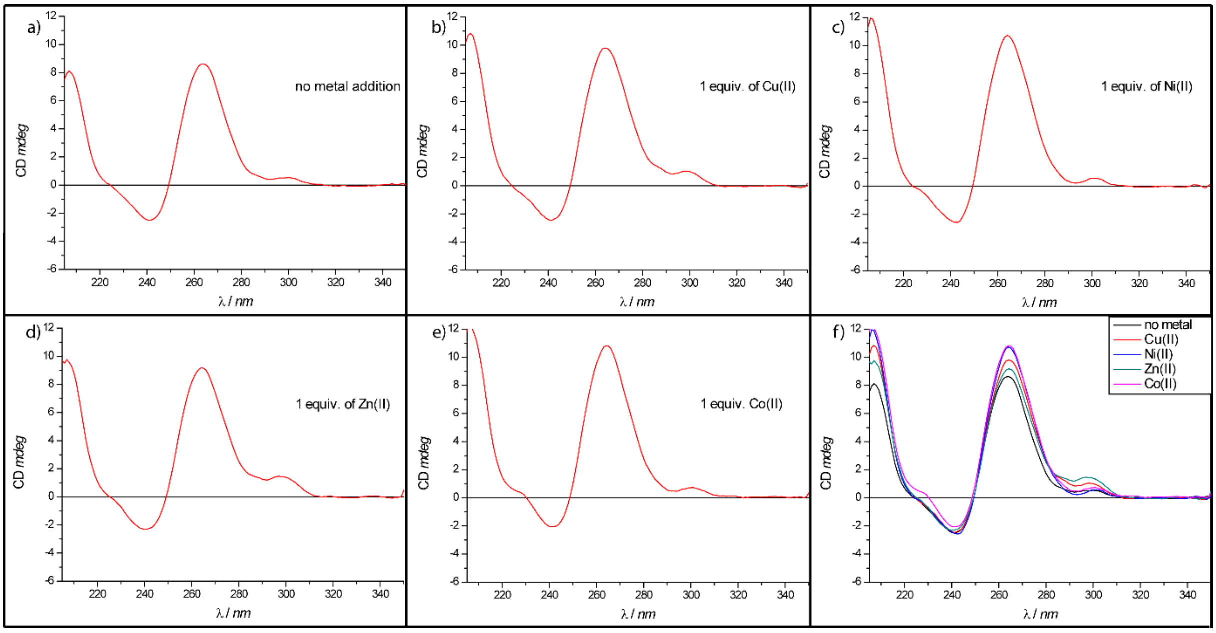

Figure 42. CD spectra of the folded G-quadruplex G_4**L**^{2R} at 4 °C with ellipticities given in milli-degree: a) without transition metals and after addition of 1 equiv. b) Cu(II), c) Ni(II), d) Zn(II) and e) Co(II); f) superposition of a)-e).

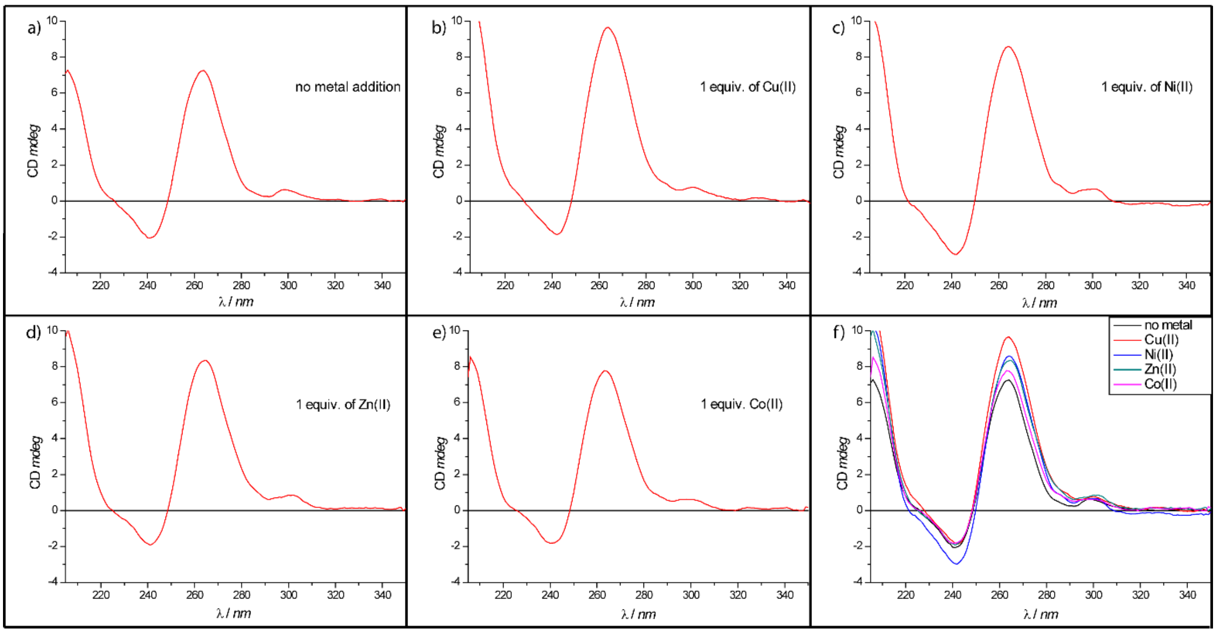

Figure 43. CD spectra of the folded G-quadruplex $G_4\mathbf{L}^{2S}$ at 4 °C with ellipticities given in milli-degree: a) without transition metals and after addition of 1 equiv. b) Cu(II), c) Ni(II), d) Zn(II) and e) Co(II); f) superposition of a)-e).

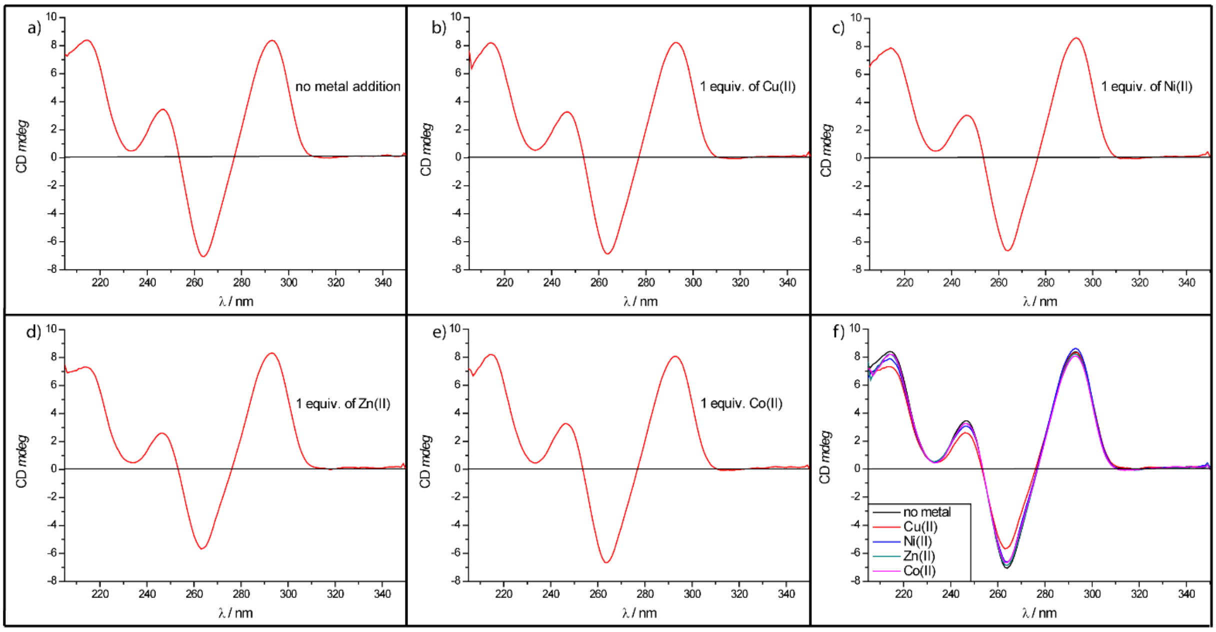

Figure 44. CD spectra of the folded G-quadruplex htel$\mathbf{L}^{1R}_4$ at 4 °C with ellipticities given in milli-degree: a) without transition metals and after addition of 1 equiv. b) Cu(II), c) Ni(II), d) Zn(II) and e) Co(II); f) superposition of a)-e).

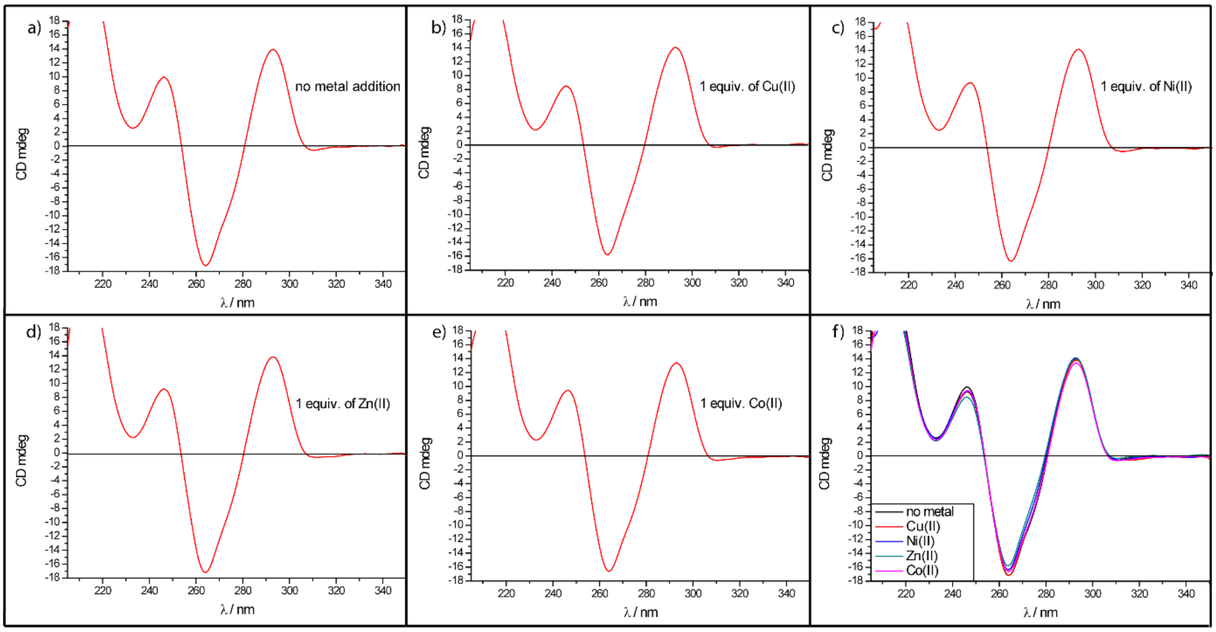

Figure 45. CD spectra of the folded G-quadruplex htel$\mathbf{L}^{1S}_4$ at 4 °C with ellipticities given in milli-degree: a) without transition metals and after addition of 1 equiv. b) Cu(II), c) Ni(II), d) Zn(II) and e) Co(II); f) superposition of a)-e).

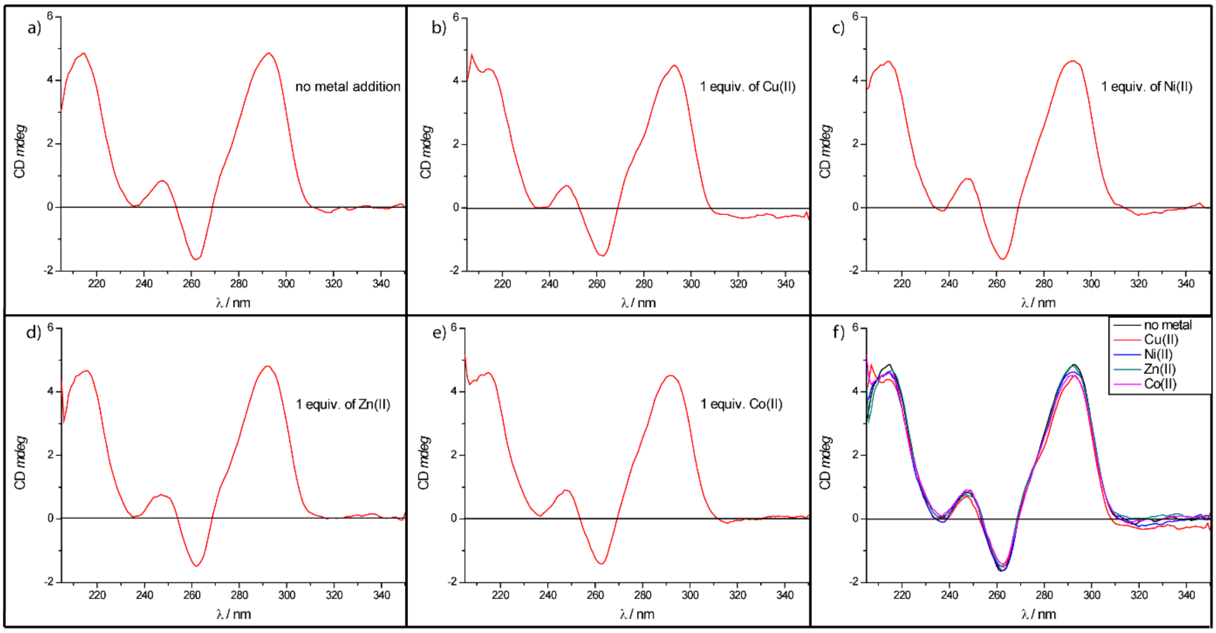

Figure 46. CD spectra of the folded G-quadruplex htel$\mathbf{L}^{2R}_2$ at 4 °C with ellipticities given in milli-degree: a) without transition metals and after addition of 1 equiv. b) Cu(II), c) Ni(II), d) Zn(II) and e) Co(II); f) superposition of a)-e).

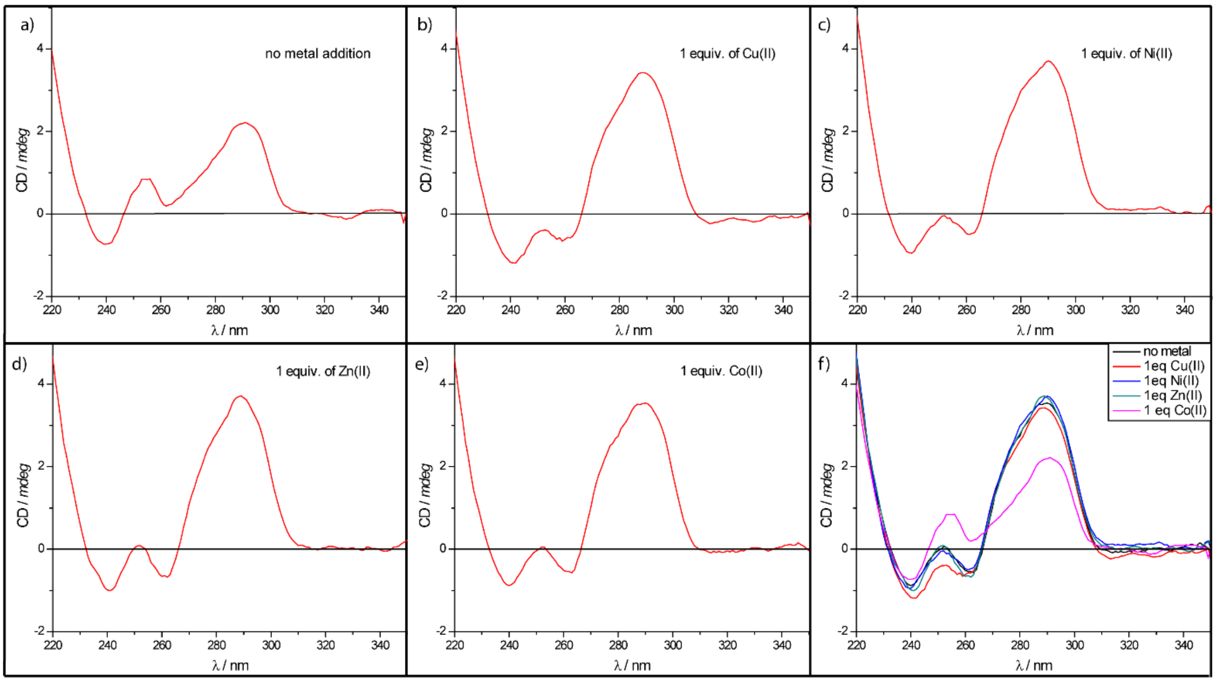

Figure 47. CD spectra of the folded G-quadruplex htel$\mathbf{L}^{2S}_2$ at 4 °C with ellipticities given in milli-degree: a) without transition metals and after addition of 1 equiv. b) Cu(II), c) Ni(II), d) Zn(II) and e) Co(II); f) superposition of a)-e).

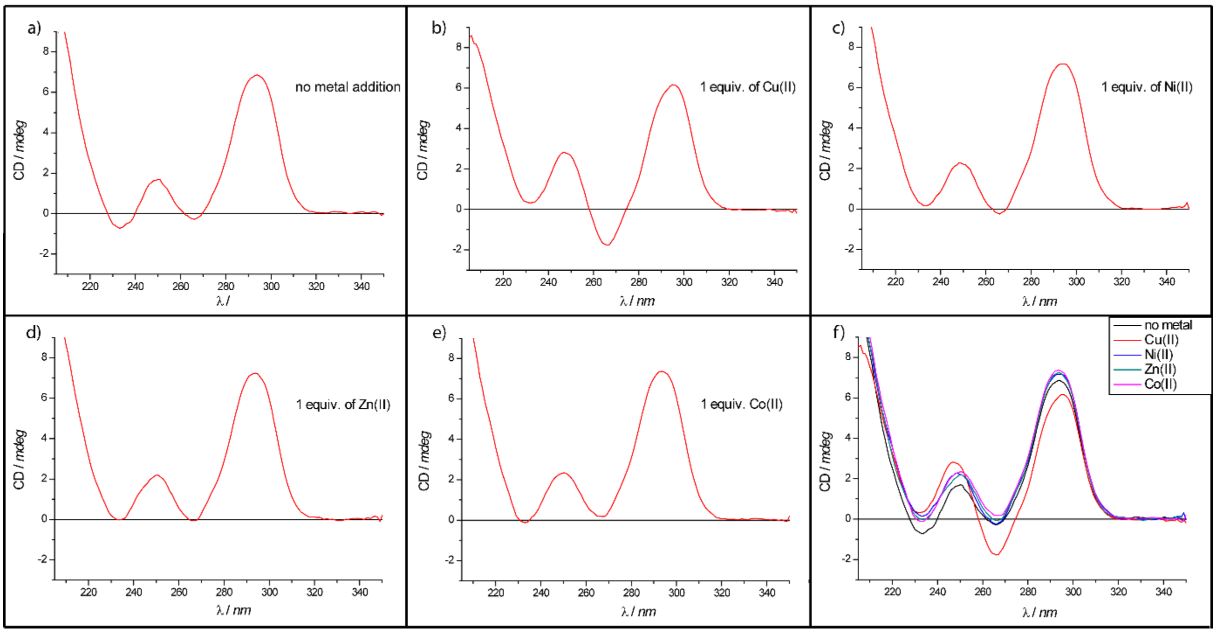

Figure 48. CD spectra of the folded G-quadruplex htel$\mathbf{L}^{2R}_2$B in NaCl at 4 °C with ellipticities given in milli-degree: a) without transition metals and after addition of 1 equiv. b) Cu(II), c) Ni(II), d) Zn(II) and e) Co(II); f) superposition of a)-e).

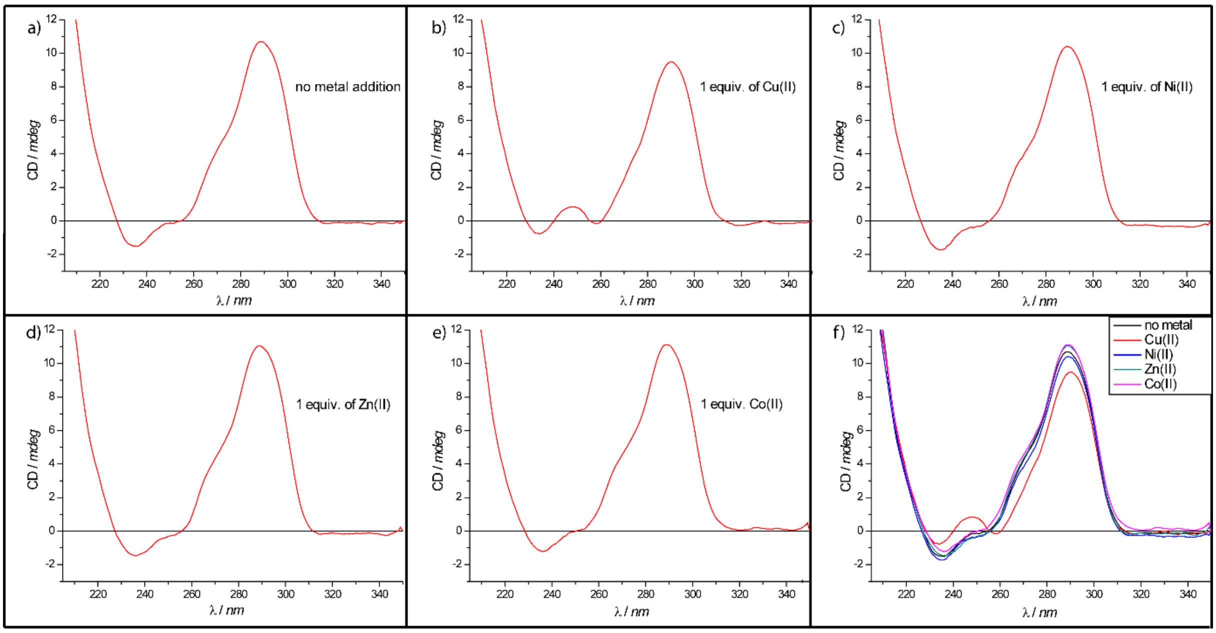

Figure 49. CD spectra of the folded G-quadruplex htel**L**$^{2R}_{2}$B in KCl at 4 °C with ellipticities given in milli-degree: a) without transition metals and after addition of 1 equiv. b) Cu(II), c) Ni(II), d) Zn(II) and e) Co(II); f) superposition of a)-e).

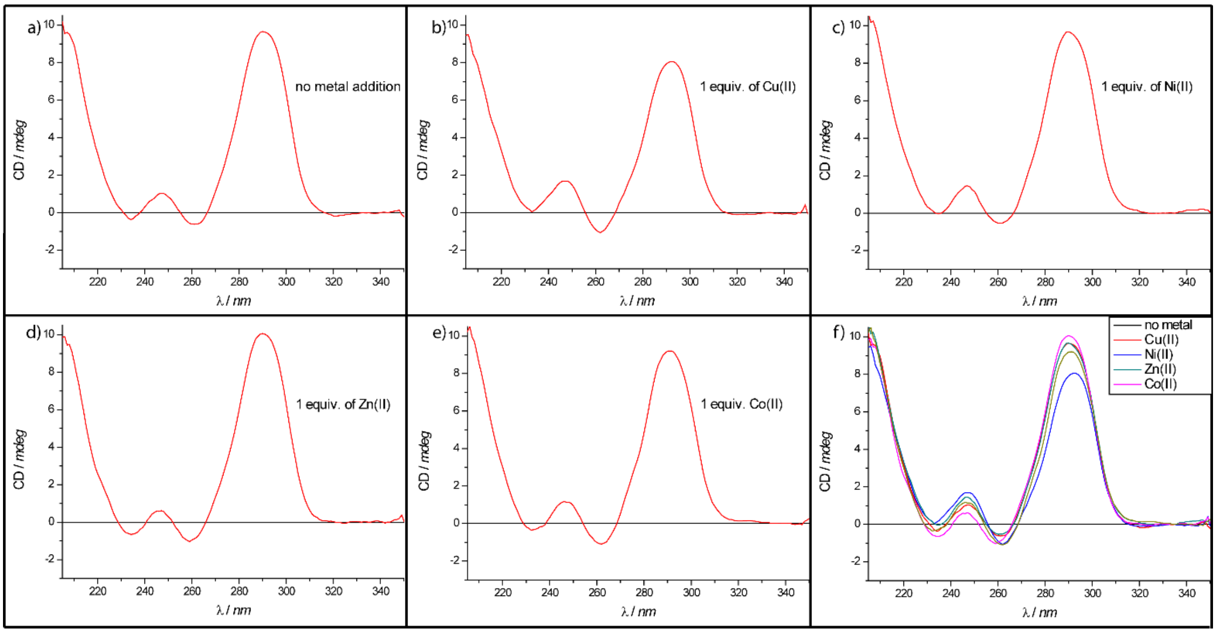

Figure 50. CD spectra of the folded G-quadruplex htel**L**$^{2S}_{2}$B in NaCl at 4 °C with ellipticities given in milli-degree: a) without transition metals and after addition of 1 equiv. b) Cu(II), c) Ni(II), d) Zn(II) and e) Co(II); f) superposition of a)-e).

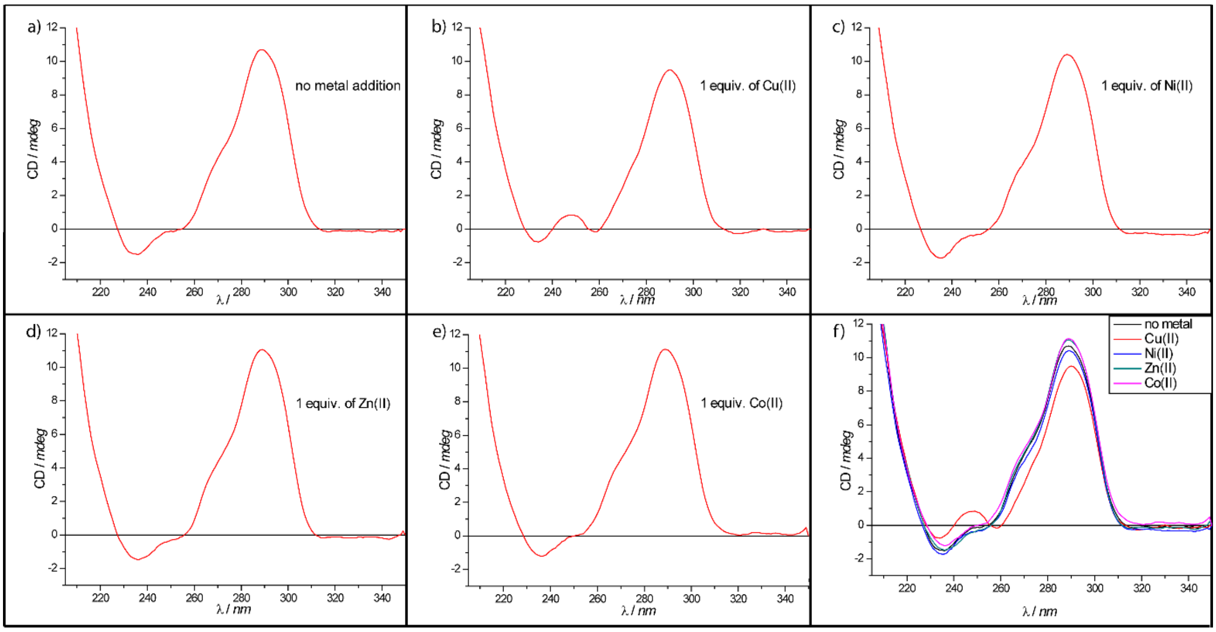

Figure 51. CD spectra of the folded G-quadruplex htel**L**$^{2S}_{2}$B in KCl at 4 °C with ellipticities given in milli-degree: a) without transition metals and after addition of 1 equiv. b) Cu(II), c) Ni(II), d) Zn(II) and e) Co(II); f) superposition of a)-e).

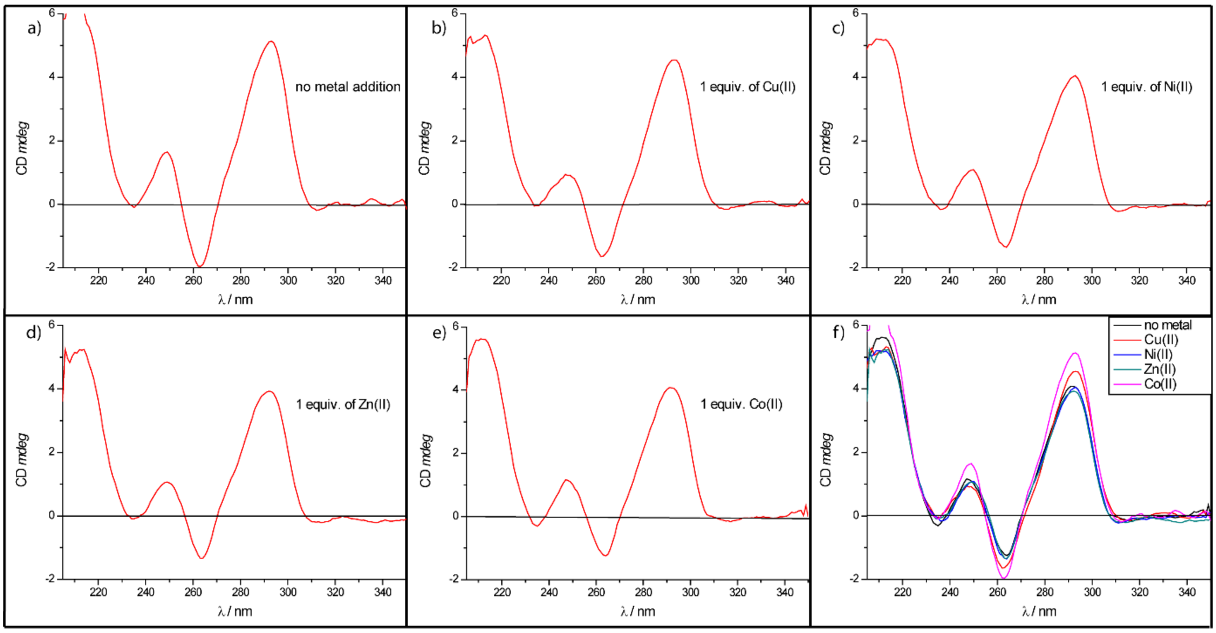

Figure 52. CD spectra of the folded G-quadruplex htel**L**$^{2R}_{3}$ at 4 °C with ellipticities given in milli-degree: a) without transition metals and after addition of 1 equiv. b) Cu(II), c) Ni(II), d) Zn(II) and e) Co(II); f) superposition of a)-e).

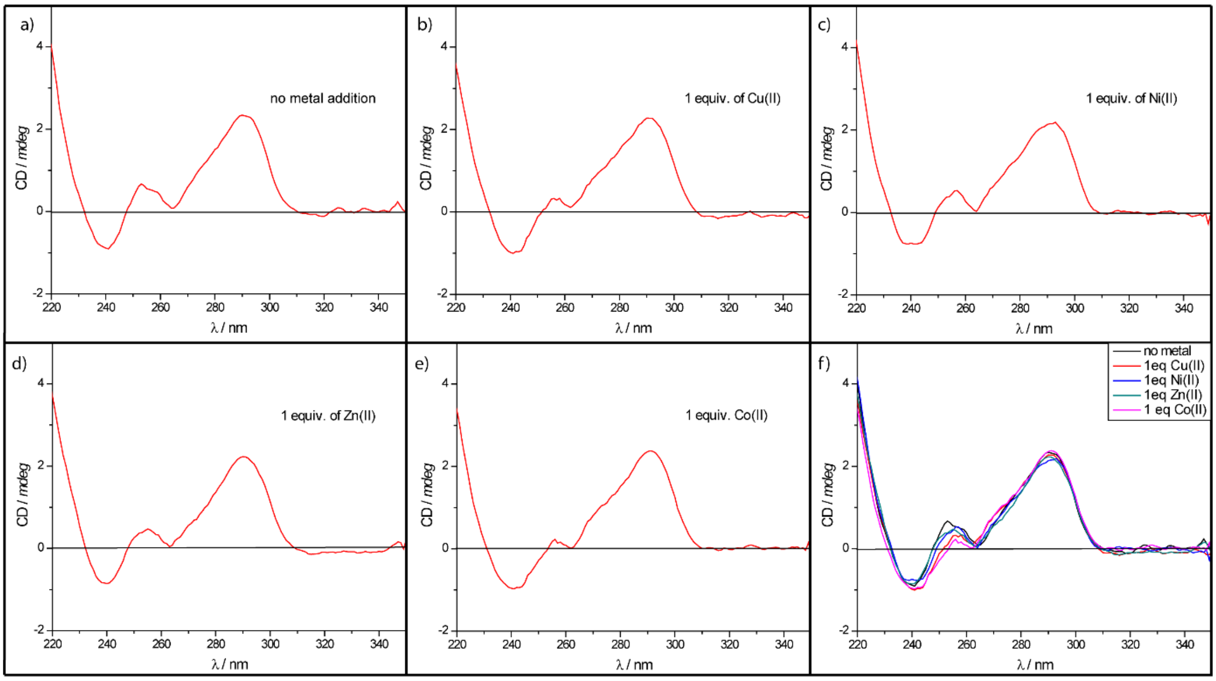

Figure 53. CD spectra of the folded G-quadruplex htel**L**$^{2S}{}_{3}$ at 4 °C with ellipticities given in milli-degree: a) without transition metals and after addition of 1 equiv. b) Cu(II), c) Ni(II), d) Zn(II) and e) Co(II); f) superposition of a)-e).

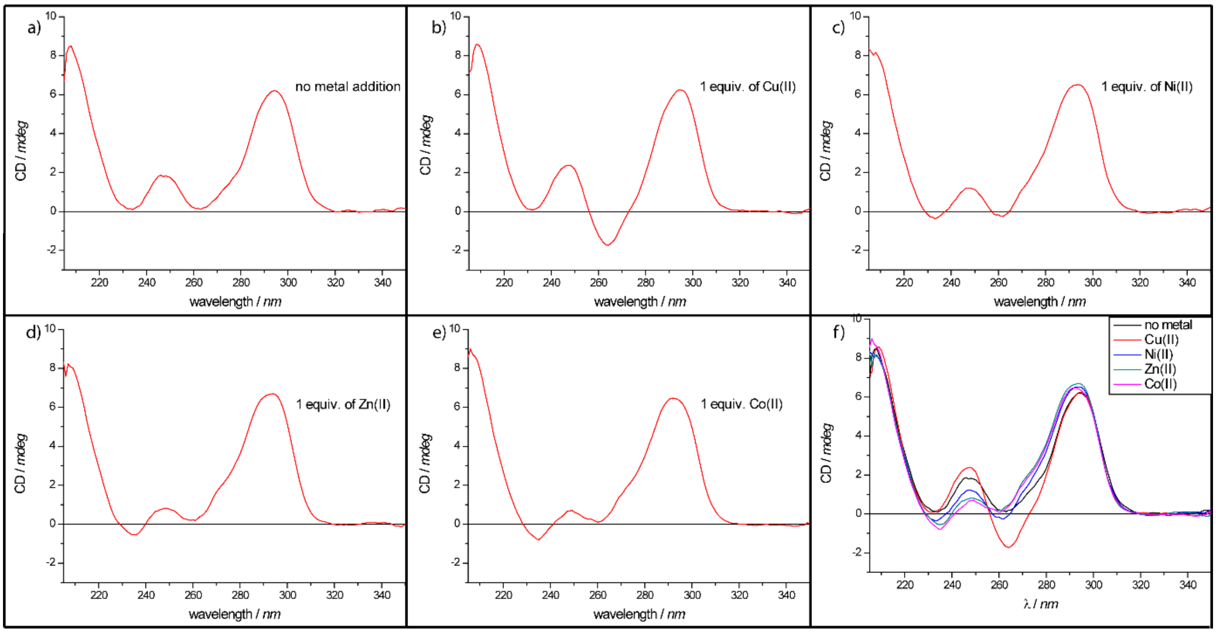

Figure 54. CD spectra of the folded G-quadruplex htel**L**$^{2S}{}_{3}$B in NaCl at 4 °C with ellipticities given in milli-degree: a) without transition metals and after addition of 1 equiv. b) Cu(II), c) Ni(II), d) Zn(II) and e) Co(II); f) superposition of a)-e).

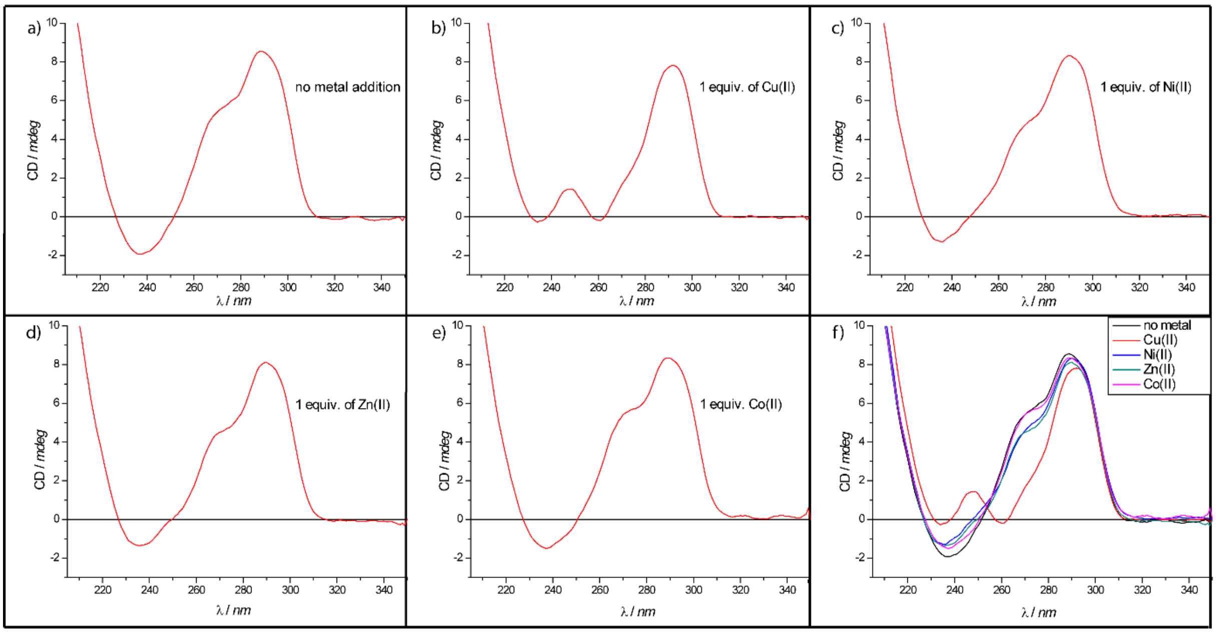

Figure 55. CD spectra of the folded G-quadruplex htel$\mathbf{L}^{2S}{}_{3}$B in KCl at 4 °C with ellipticities given in milli-degree: a) without transition metals and after addition of 1 equiv. b) Cu(II), c) Ni(II), d) Zn(II) and e) Co(II); f) superposition of a)-e).

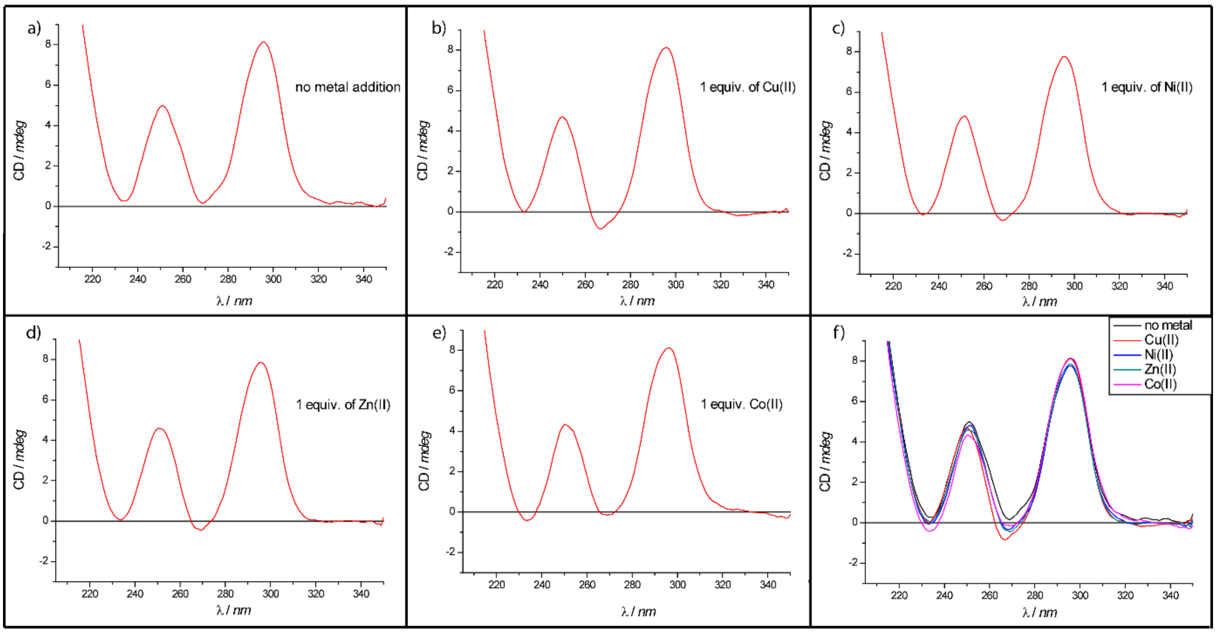

Figure 56. CD spectra of the folded G-quadruplex htel$\mathbf{L}^{2R}{}_{3}$B in NaCl at 4 °C with ellipticities given in milli-degree: a) without transition metals and after addition of 1 equiv. b) Cu(II), c) Ni(II), d) Zn(II) and e) Co(II); f) superposition of a)-e).

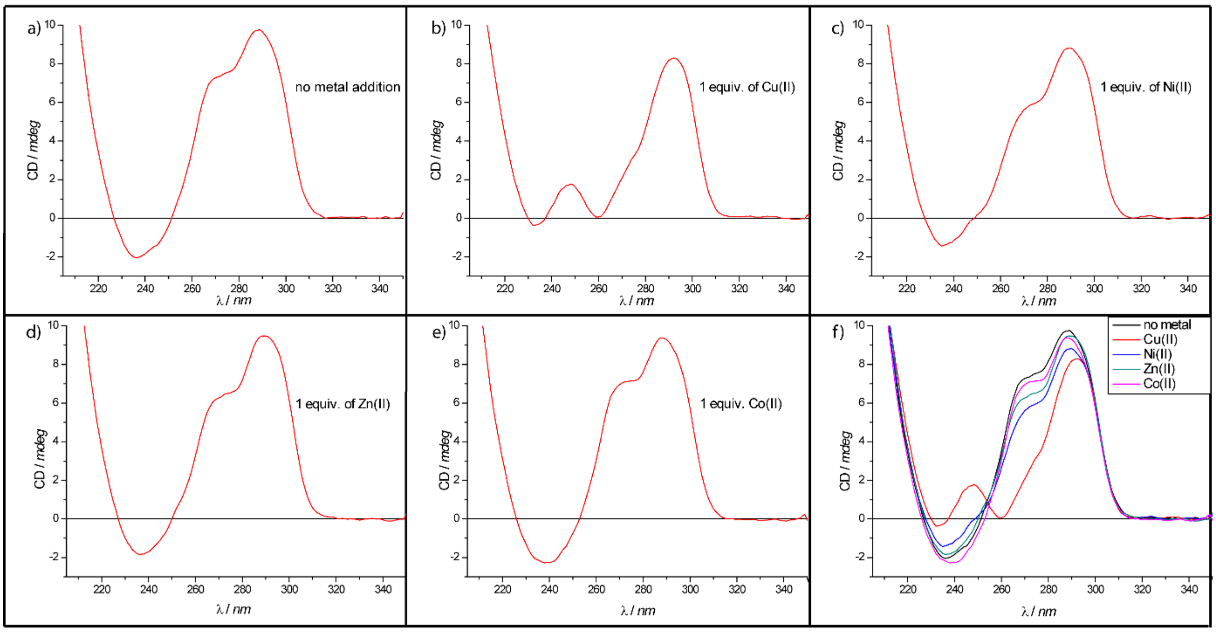

Figure 57. CD spectra of the folded G-quadruplex htel**L**$^{2R}{}_{3}$B in KCl at 4 °C with ellipticities given in milli-degree: a) without transition metals and after addition of 1 equiv. b) Cu(II), c) Ni(II), d) Zn(II) and e) Co(II); f) superposition of a)-e).

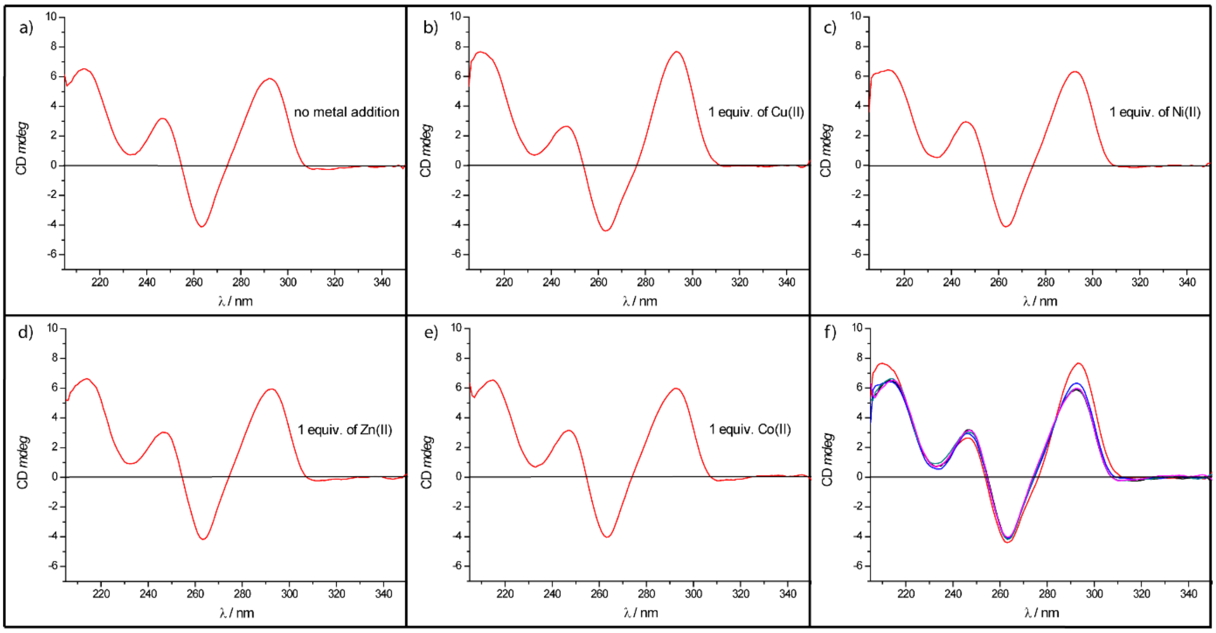

Figure 58. CD spectra of the folded G-quadruplex htel**L**$^{2R}{}_{4}$ at 4 °C with ellipticities given in milli-degree: a) without transition metals and after addition of 1 equiv. b) Cu(II), c) Ni(II), d) Zn(II) and e) Co(II); f) superposition of a)-e).

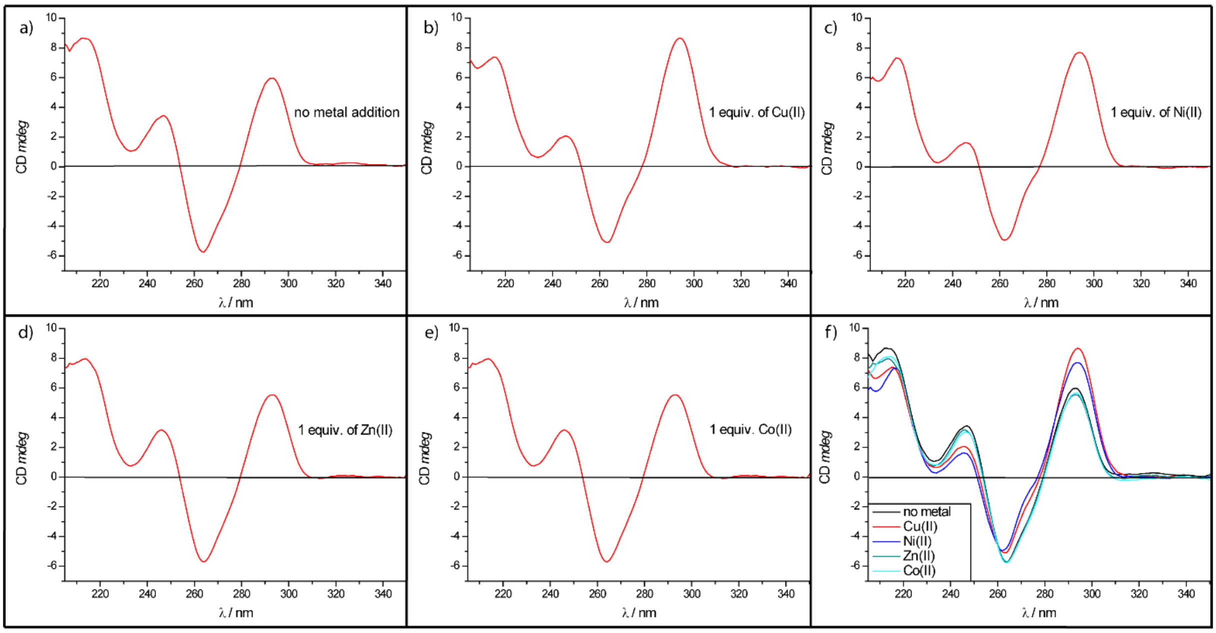

Figure 59. CD spectra of the folded G-quadruplex htel$\mathbf{L}^{2S}_4$ at 4 °C with ellipticities given in milli-degree: a) without transition metals and after addition of 1 equiv. b) Cu(II), c) Ni(II), d) Zn(II) and e) Co(II); f) superposition of a)-e).

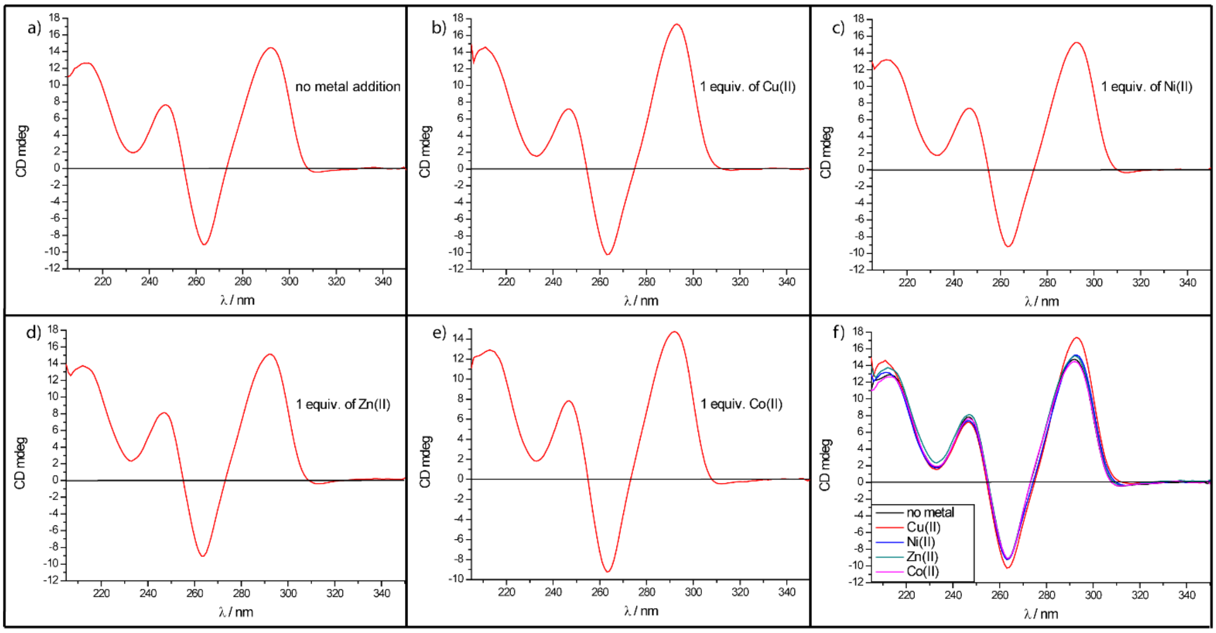

Figure 60. CD spectra of the folded G-quadruplex htel$\mathbf{L}^{2R}_5$ at 4 °C with ellipticities given in milli-degree: a) without transition metals and after addition of 1 equiv. b) Cu(II), c) Ni(II), d) Zn(II) and e) Co(II); f) superposition of a)-e).

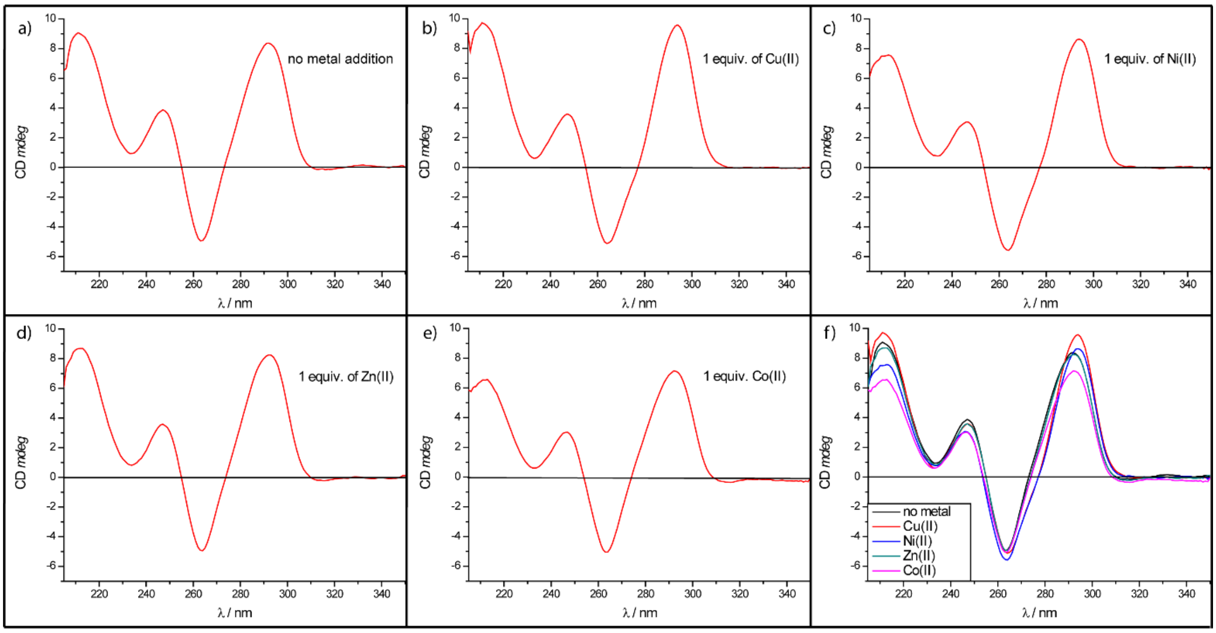

Figure 61. CD spectra of the folded G-quadruplex htel$\mathbf{L}^{2S}{}_{5}$ at 4 °C with ellipticities given in milli-degree: a) without transition metals and after addition of 1 equiv. b) Cu(II), c) Ni(II), d) Zn(II) and e) Co(II); f) superposition of a)-e).

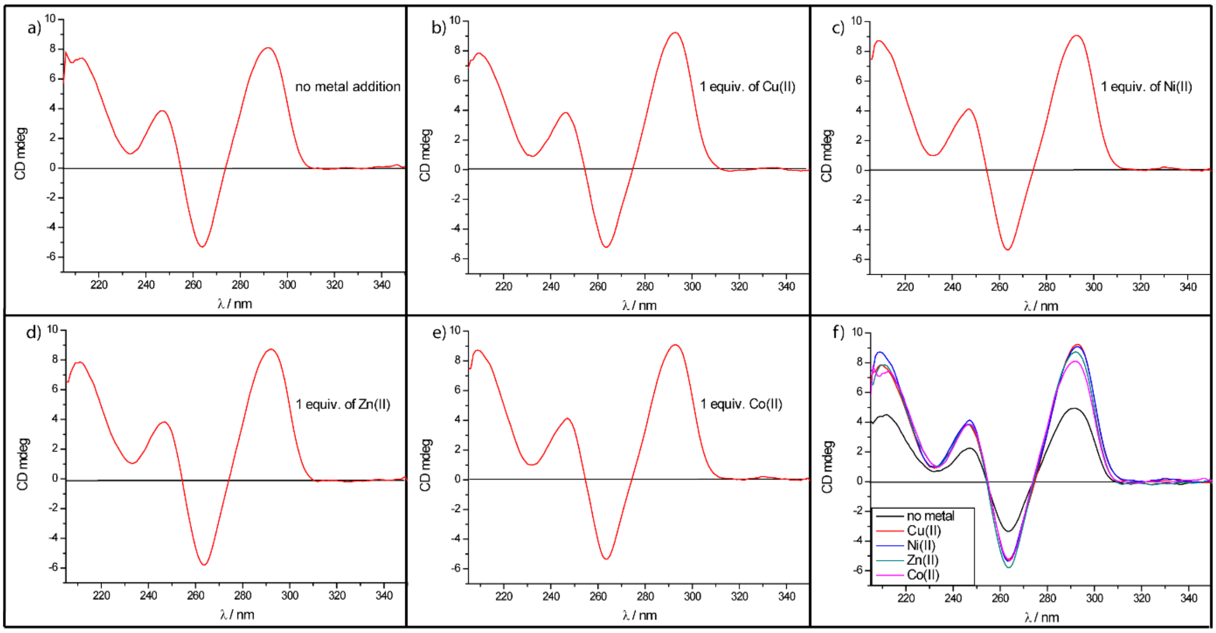

Figure 62. CD spectra of the folded G-quadruplex htel$\mathbf{L}^{2R}{}_{6}$ at 4 °C with ellipticities given in milli-degree: a) without transition metals and after addition of 1 equiv. b) Cu(II), c) Ni(II), d) Zn(II) and e) Co(II); f) superposition of a)-e).

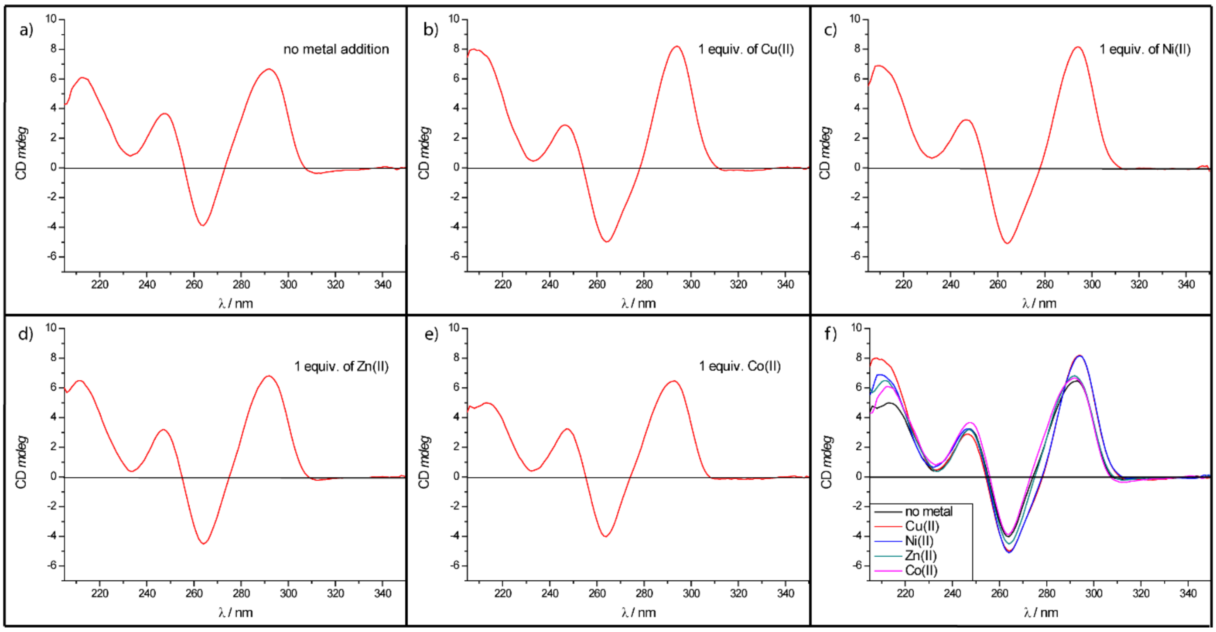

Figure 63. C CD spectra of the folded G-quadruplex htel$\mathbf{L}^{2S}_{6}$ at 4 °C with ellipticities given in milli-degree: a) without transition metals and after addition of 1 equiv. b) Cu(II), c) Ni(II), d) Zn(II) and e) Co(II); f) superposition of a)-e).

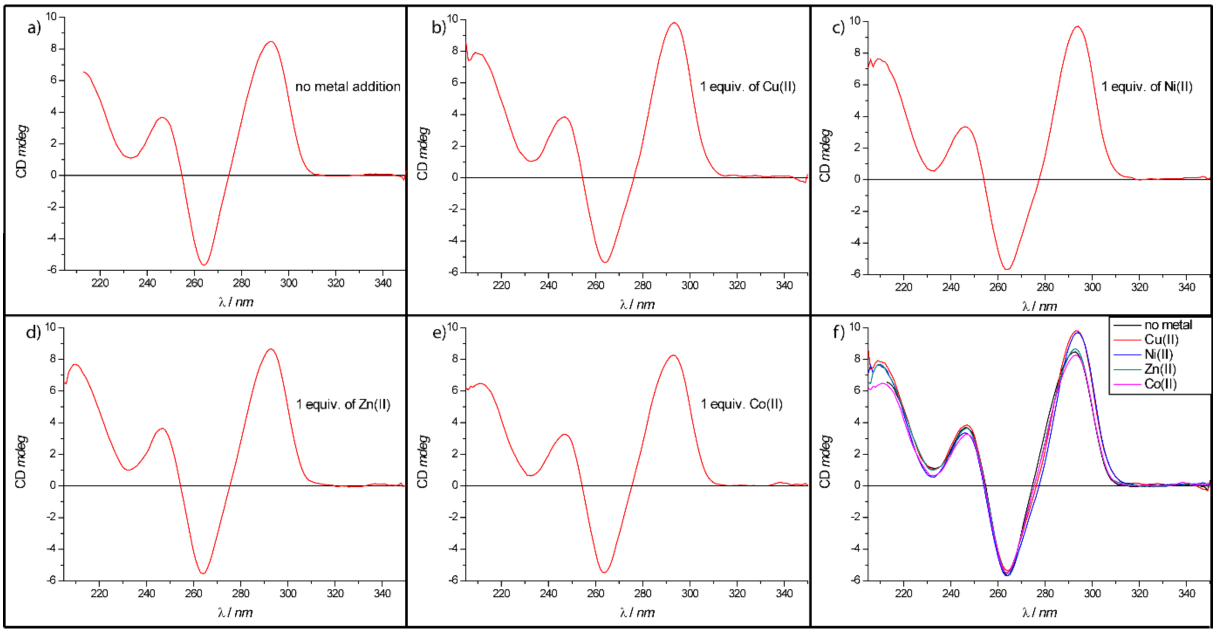

Figure 64. CD spectra of the folded G-quadruplex htel$\mathbf{L}^{2S}_{7}$ at 4 °C with ellipticities given in milli-degree: a) without transition metals and after addition of 1 equiv. b) Cu(II), c) Ni(II), d) Zn(II) and e) Co(II); f) superposition of a)-e).

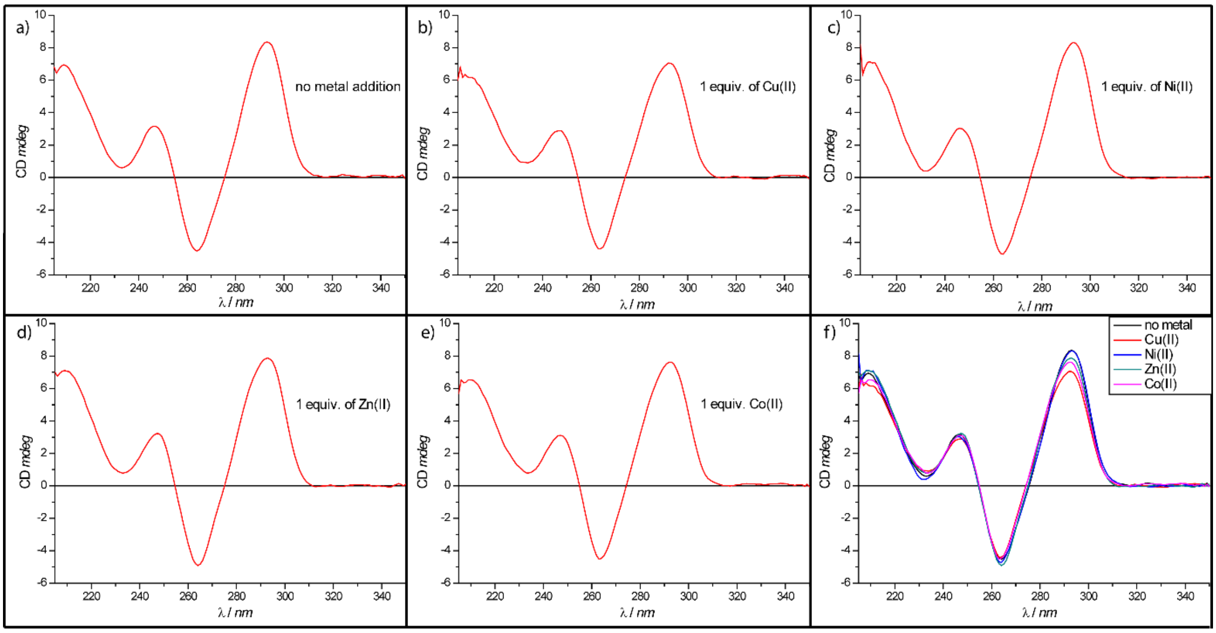

Figure 65. CD spectra of the folded G-quadruplex htel$\mathbf{L}^{2R}_{7}$ at 4 °C with ellipticities given in milli-degree: a) without transition metals and after addition of 1 equiv. b) Cu(II), c) Ni(II), d) Zn(II) and e) Co(II); f) superposition of a)-e).

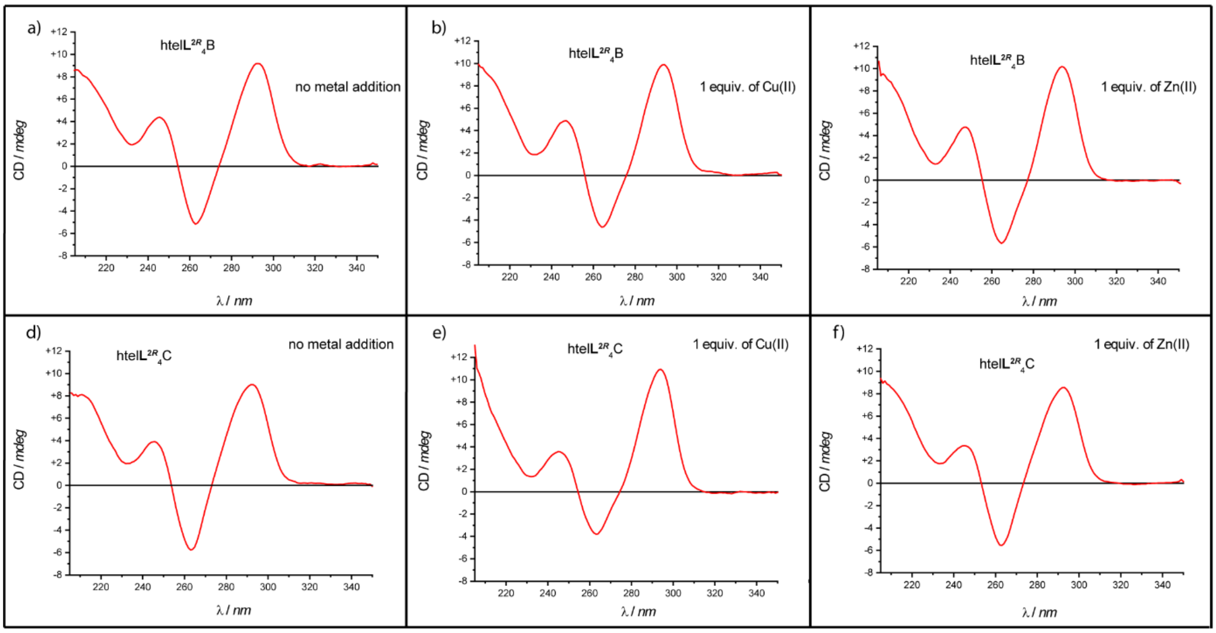

Figure 66. CD spectra of the folded G-quadruplex htel$\mathbf{L}^{2R}_{4}$B and htel$\mathbf{L}^{2R}_{4}$C at 4 °C with ellipticities given in milli-degree: a) htel$\mathbf{L}^{2R}_{4}$B without transition metal and with 1 equiv. b) Cu(II), c) Zn(II). d) htel$\mathbf{L}^{2R}_{4}$C without transition metal and with 1 equiv. e) Cu(II), f) Zn(II).

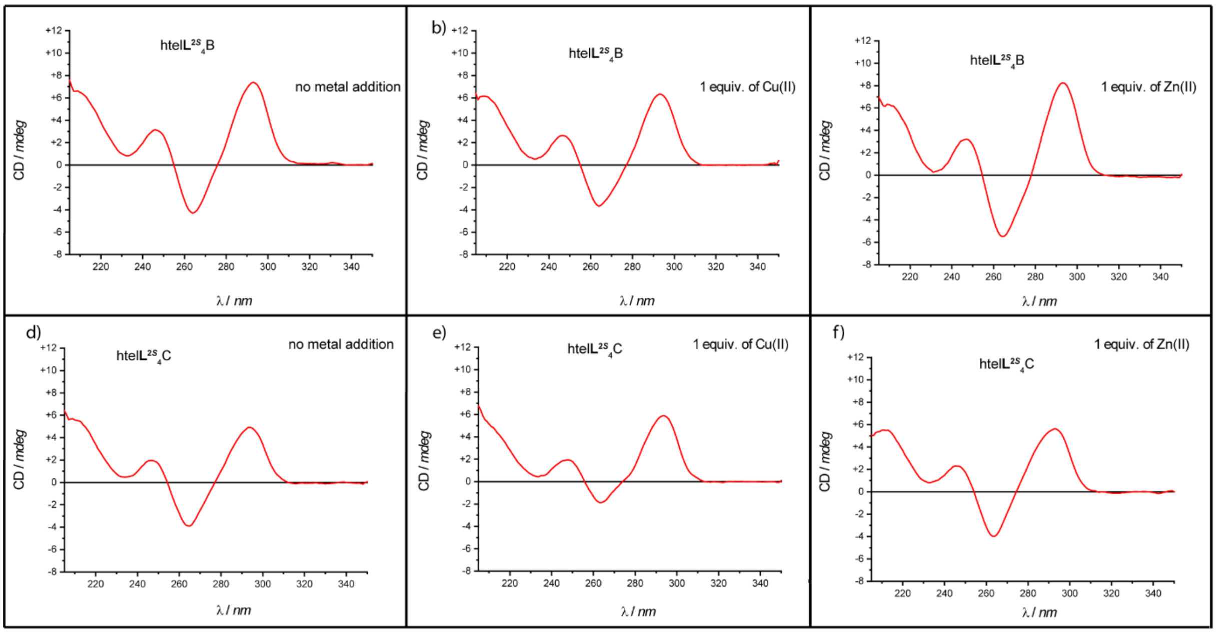

Figure 67. CD spectra of the folded G-quadruplex htel$\mathbf{L}^{2S}_4$B and htel$\mathbf{L}^{2S}_4$C at 4 °C with ellipticities given in milli-degree: a) htel$\mathbf{L}^{2S}_4$B without transition metal and with 1 equiv. b) Cu(II), c) Zn(II). d) htel$\mathbf{L}^{2S}_4$C without transition metal and with 1 equiv. e) Cu(II), f) Zn(II).

6 Native ESI-MS

In native ESI-MS, the secondary structure is kept intact. To differentiate between folded and unfolded G-quadruplexes in the gas phase, two phenomena are most instructive. First, if tetramolecular G-quadruplexes are investigated and the structure is denatured, single-stranded DNA instead of a tetramer would be observed and second, valid for tetra- and unimolecular G-quadruplexes, ESI mass spectrometry from electrolyte-containing solutions always gives rise to series of unspecific adducts with sodium or potassium cations. For fully denatured species, a statistical distribution of adducts starting with zero cations would be observed and for a native, folded species a distribution is observed starting with n-1 explicitly bound cations where n is the number of G-tetrads.

Ion mobility measurements were performed on a Bruker timsTOF instrument combining a trapped ion mobility spectrometer (TIMS) with a time-of-flight (TOF) mass spectrometer in one instrument.

In contrast to the conventional drift tube method to determine mobility data, where ions are carried by an electric field through a stationary drift gas, the TIMS method is based on an electric field ramp to hold ions in place against a carrier gas pushing them in the direction of the analyzer. Consequently, larger sized ions that experience more carrier gas impacts leave the TIMS units first and smaller ions elute later. This method offers a much higher mobility resolution despite a smaller device size.

Measurement: After the generation of ions by electrospray ionisation (ESI, analyte concentration: 12.5 µM G-quadruplex, 0.5 mM KCl and 50 mM TMAA (trimethyl ammonium acetate) pH 6.8, solvent: acetonitrile-water, 1:1, capillary voltage: 4500 V, end plate offset voltage: 500 nebulizer gas pressure: 0.4/0.6 bar, dry gas flow rate: 6/9 l min^{-1}, dry temperature: 303 K), the desired ions were orthogonally deflected into the TIMS cell consisting of an entrance funnel and the TIMS analyser (carrier gas: N_2, temperature: 305 K, entrance pressure: 2.55 mbar, exit pressure: 0.89 mbar, IMS imeX ramp end: 1.66 1/K0, IMS

imeX ramp start: 0.51 1/K0). As a result, the ions are stationary trapped. After accumulation (accumulation time: 70/200 ms), a stepwise reduction of the electric field strength leads to a release of ion packages separated by their mobility. After a subsequent focussing, the separated ions are transferred to the TOF-analyser. [28-30]

The ion mobility *K* was directly calculated from the trapping electric field strength *E* and the velocity of the carrier gas stream v_g *via*

$$K = \frac{v_g}{E} = \frac{A}{U_{release} - U_{out}} \tag{1}$$

where *A* is a calibration constant (based on calibration standards), $U_{release}$ is the voltage at which the ions are released from the analyser and U_{out} is the voltage applied to the exit of the tube. The ion mobility is corrected to standard gas density *via*

$$K_0 = K \frac{P}{1013\ \mathrm{hPa}} \frac{237\ \mathrm{K}}{T} \tag{2}$$

to obtain the reduced mobility K_0, where *P* is the pressure and *T* is the temperature. By using the Mason-Schamp equation, the collisional cross-section Ω can be calculated:

$$\Omega = \frac{(18\pi)^{\frac{1}{2}}}{16} \frac{ze}{(k_B T)^{\frac{1}{2}}} \left[\frac{1}{\mu}\right]^{\frac{1}{2}} \frac{1}{K_0} \frac{1}{N_0} \tag{3}$$

where *ze* is the ion charge, k_B is the Boltzmann constant, μ is the reduced mass of analyte and carrier gas and N_0 is the number density of the neutral gas.[28-30]

For calibration of both the TIMS and TOF analysers, commercially available Agilent ESI tuning mix was used. The instrument was calibrated before each measurement, including each change in the ion mobility resolution mode ("imeX" settings: survey, detect or ultra).

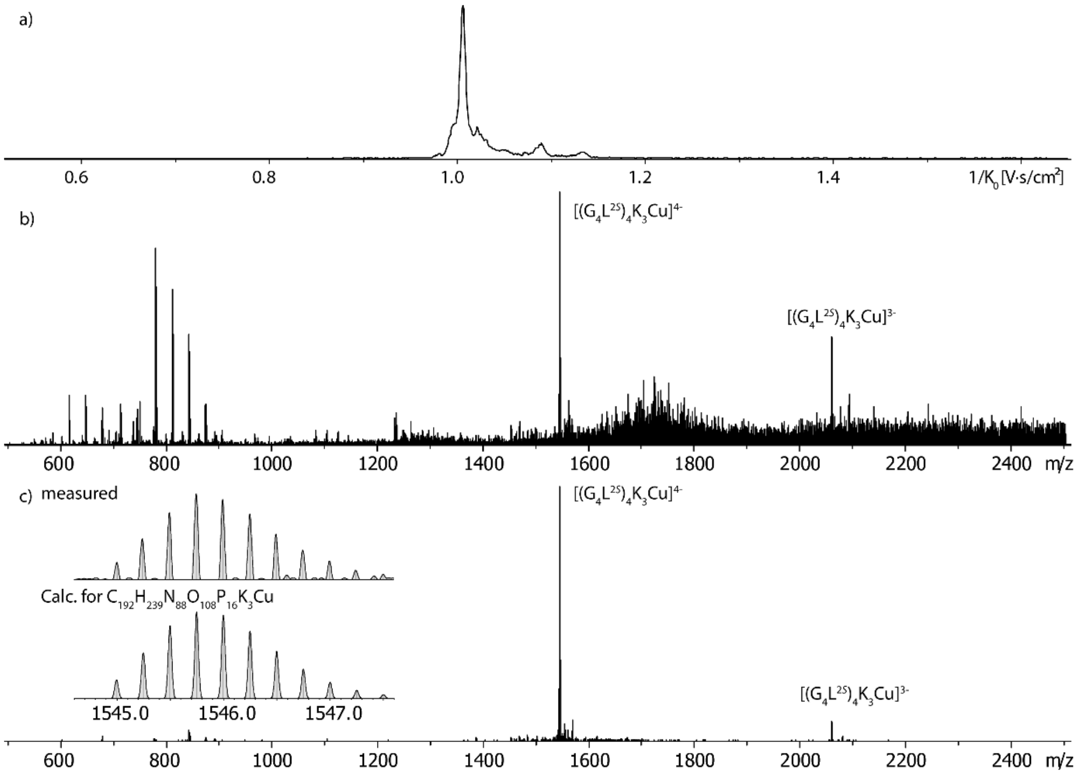

Figure 68. Native ESI-MS coupled with trapped ion mobility of G_4**L**^{2S} with 1 equiv. Cu(II): a) ion mobility distribution, b) measured mass spectrum and c) mobility-extracted mass spectrum.

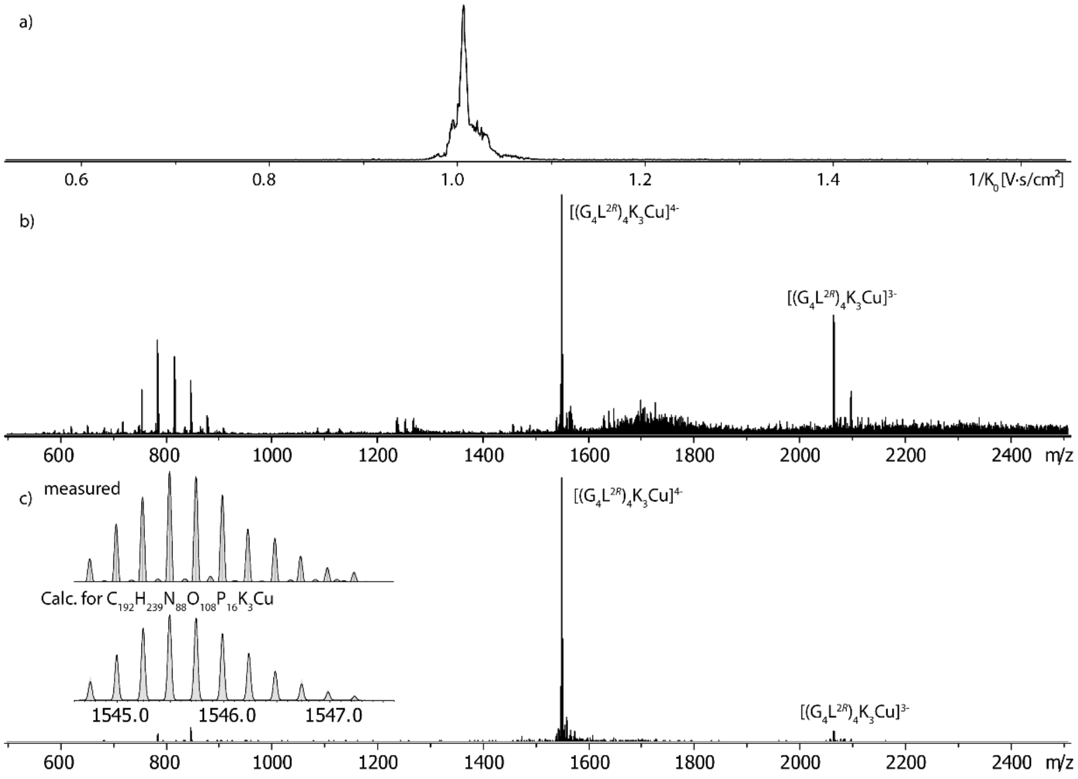

Figure 69. Native ESI-MS coupled with trapped Ion Mobility of $G_4\mathbf{L}^{2R}$ with 1 equiv. Cu(II). In a) ion mobility distribution, b) measured mass spectrum and c) mobility-extracted mass spectrum.

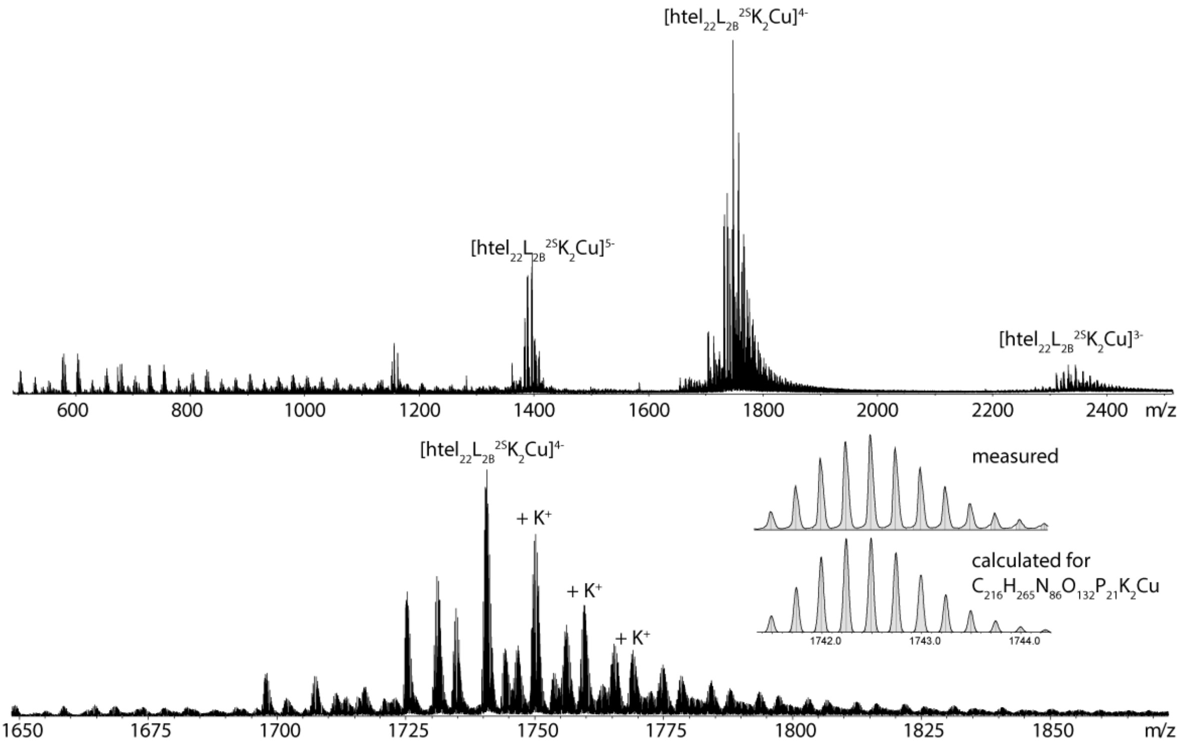

Figure 70. Native ESI-MS of htel$\mathbf{L}^{2S}{}_{2}$B with 1 equiv. Cu(II).

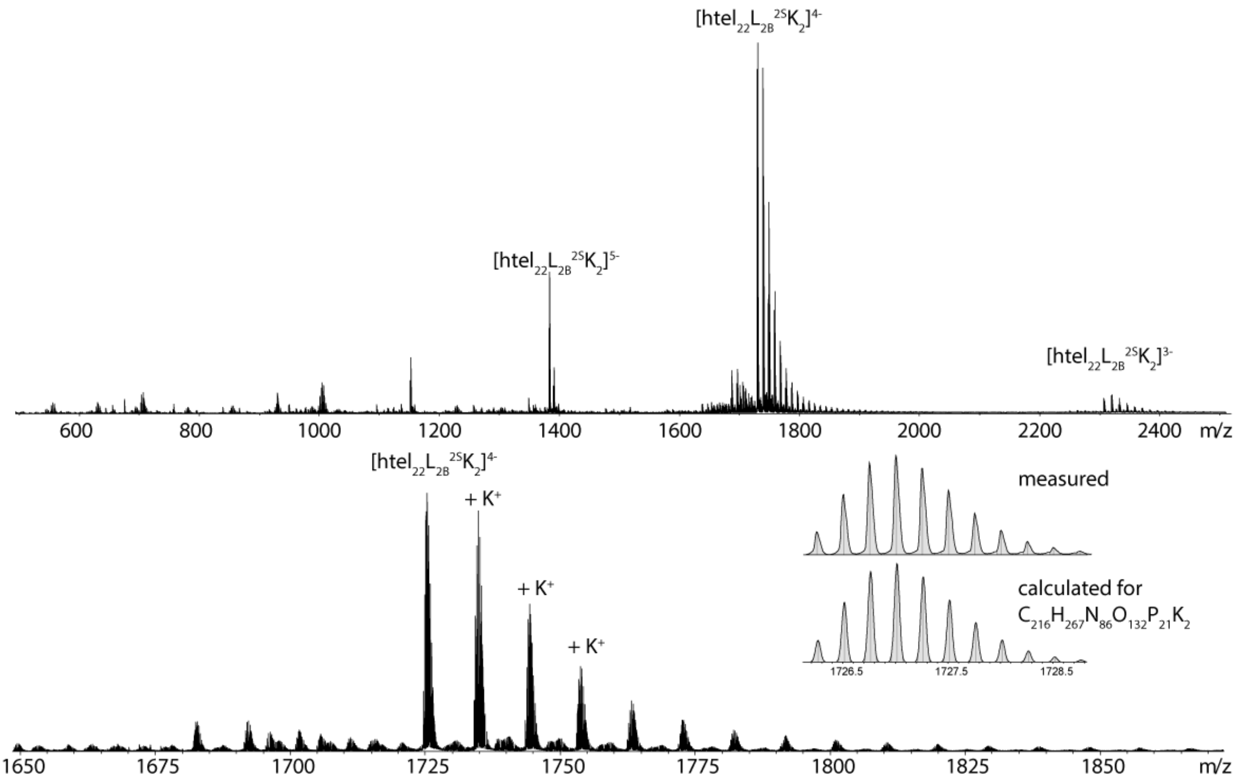

Figure 71. Native ESI-MS of htel$\mathbf{L}^{2S}{}_{2}$B in absence of transition metals.

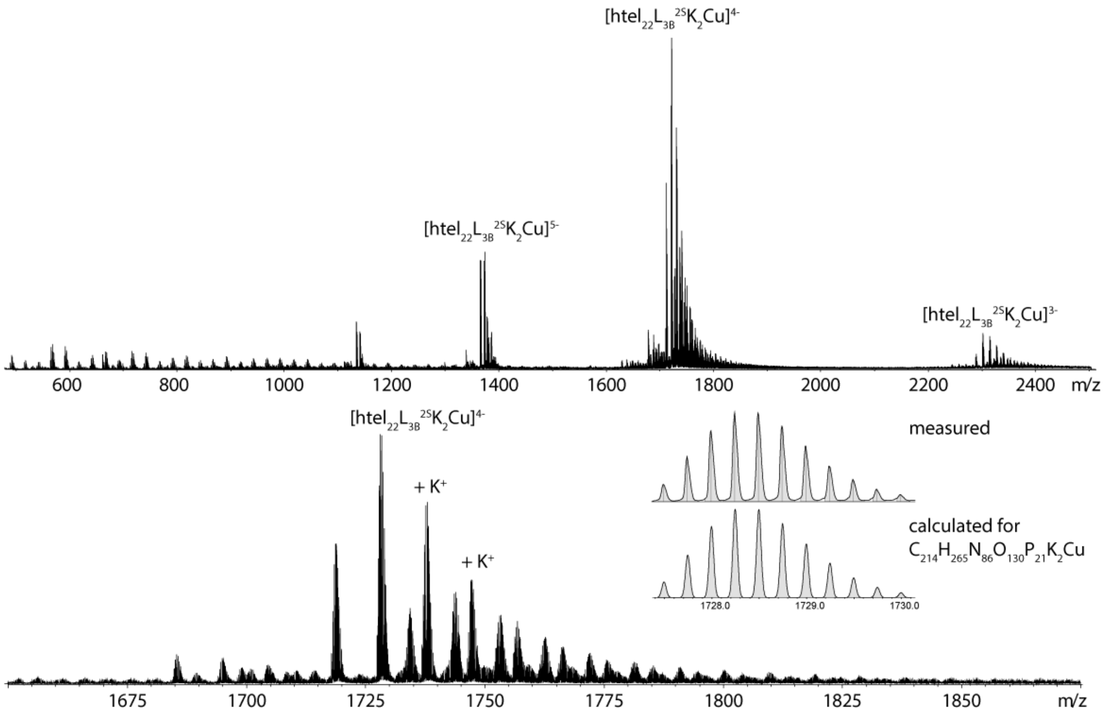

Figure 72. Native ESI-MS of htel$\mathbf{L}^{2S}{}_{3}$B with 1 equiv. Cu(II).

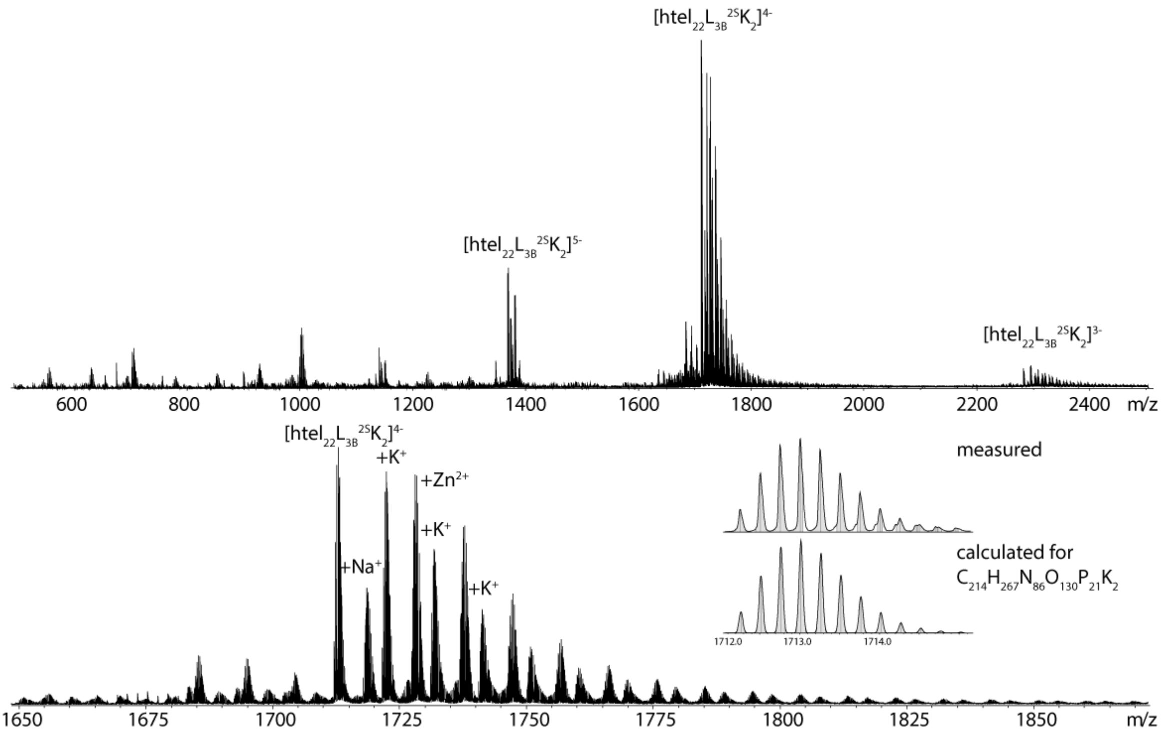

Figure 73. Native ESI-MS of htel$\mathbf{L}^{2S}{}_{3}$B in absence of transition metals

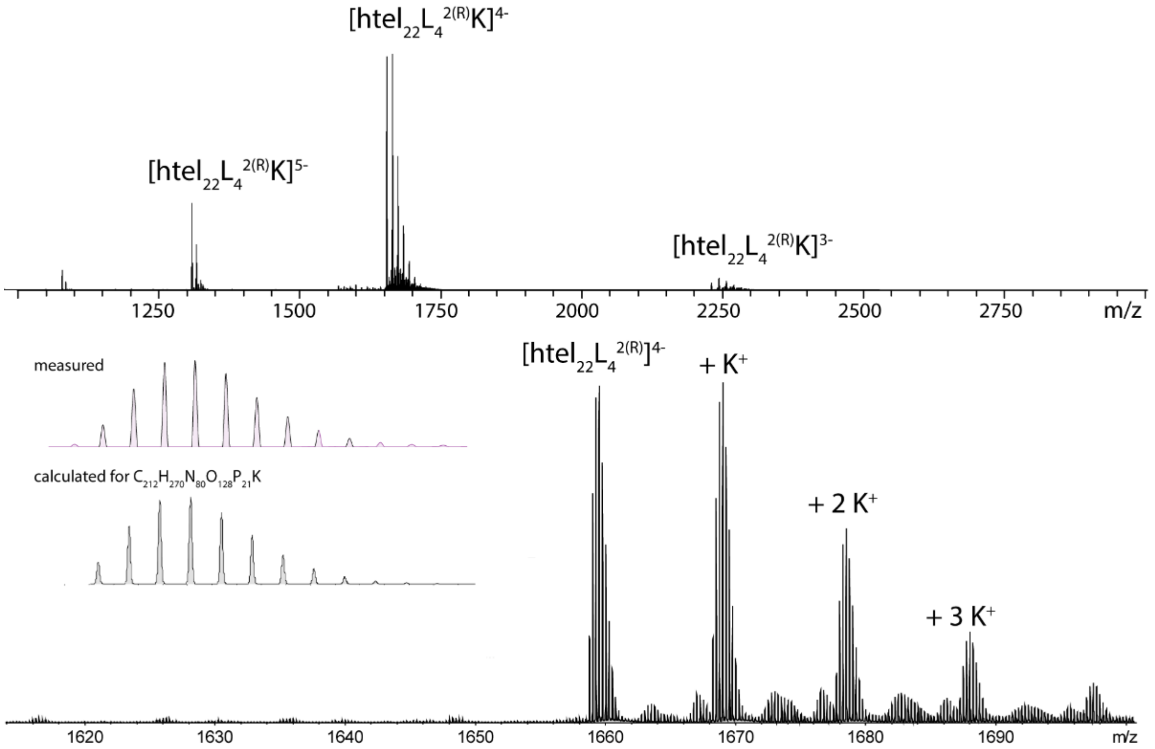

Figure 74. Native ESI-MS of htel$\mathbf{L}^{2R}{}_{4}$ in absence of any transition metal.

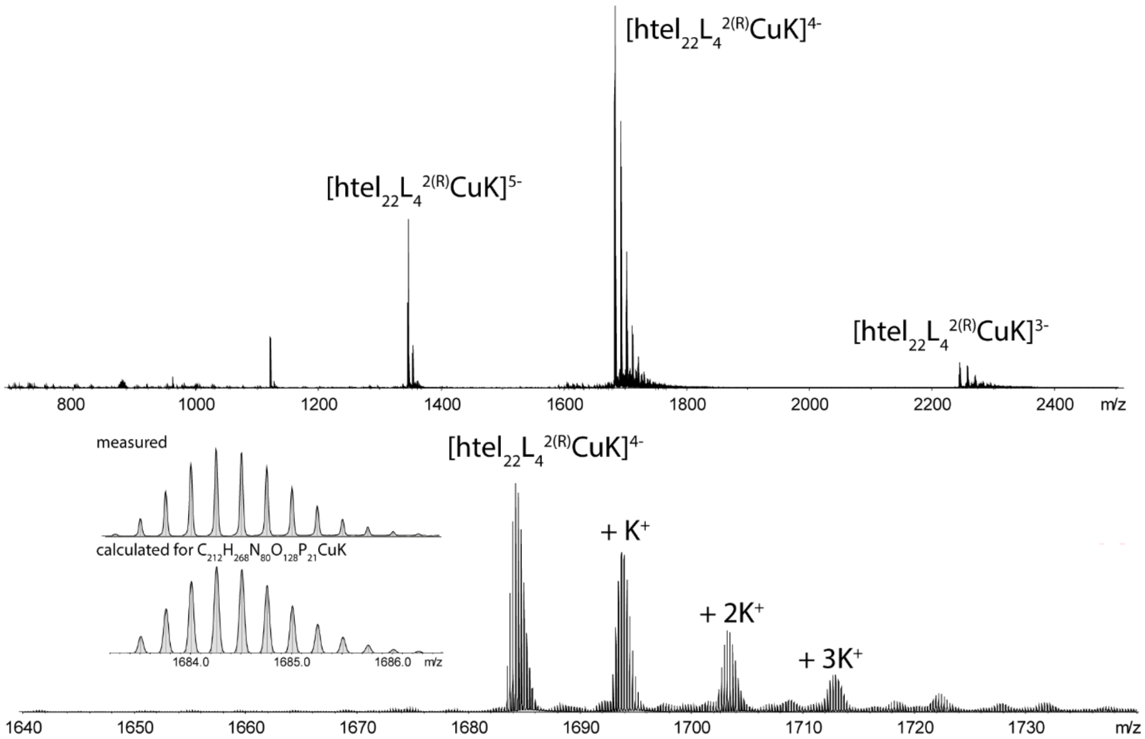

Figure 75. Native ESI-MS of htel**L**$^{2R}_4$ with 1 equiv. Cu(II).

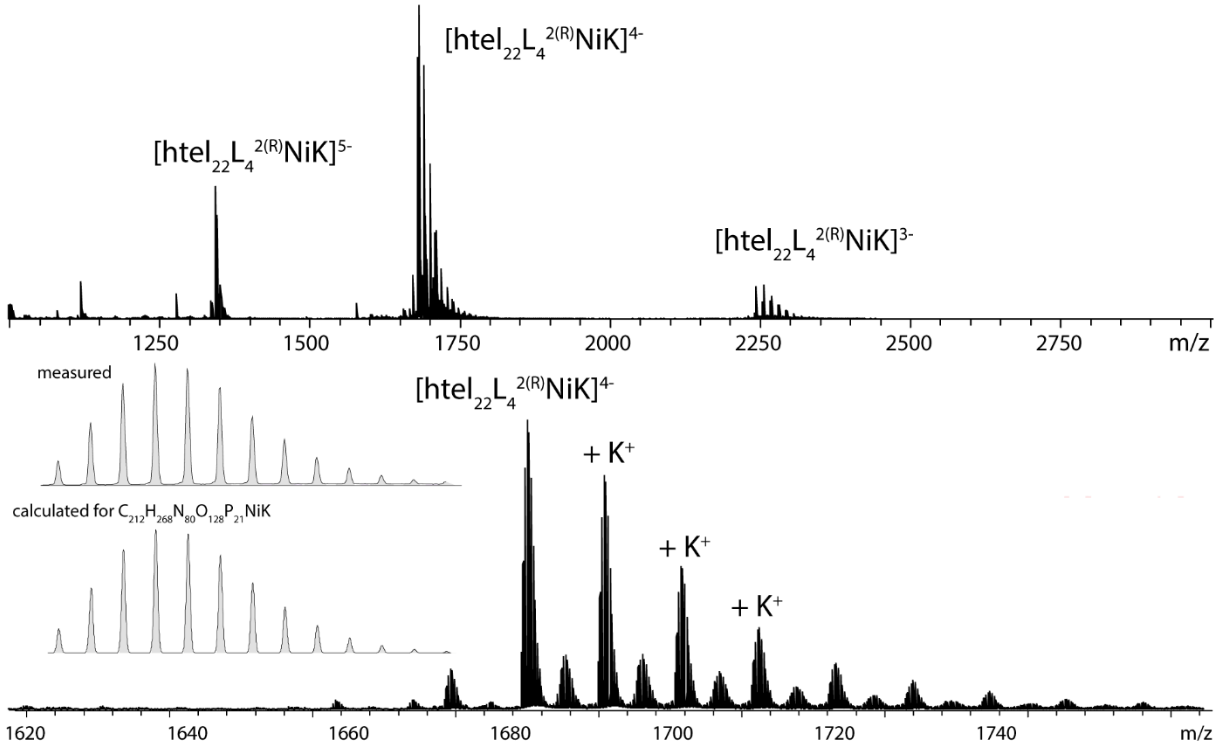

Figure 76. Native ESI-MS of htel**L**$^{2R}_4$ with 1 equiv. Ni(II).

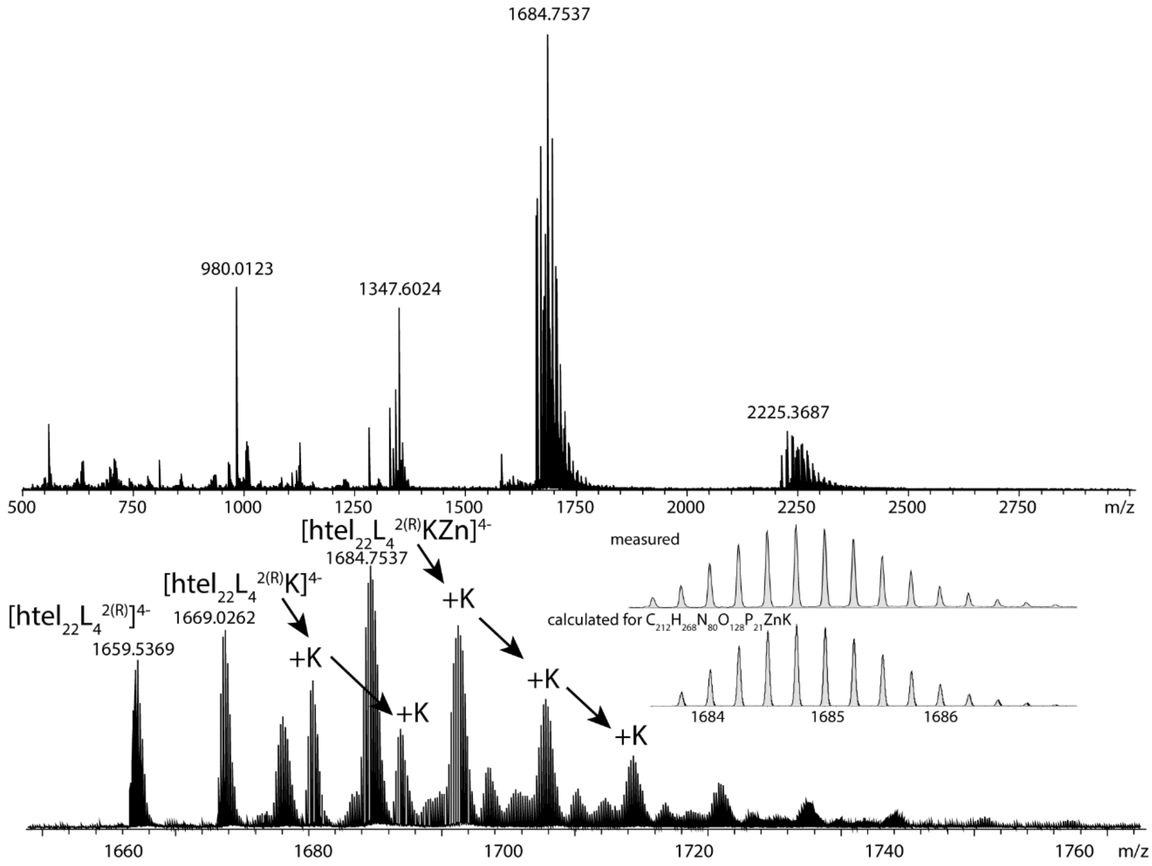

Figure 77. Native ESI-MS of htel$\mathbf{L}^{\mathbf{2}\boldsymbol{R}}{}_4$ with 1 equiv. Zn(II).

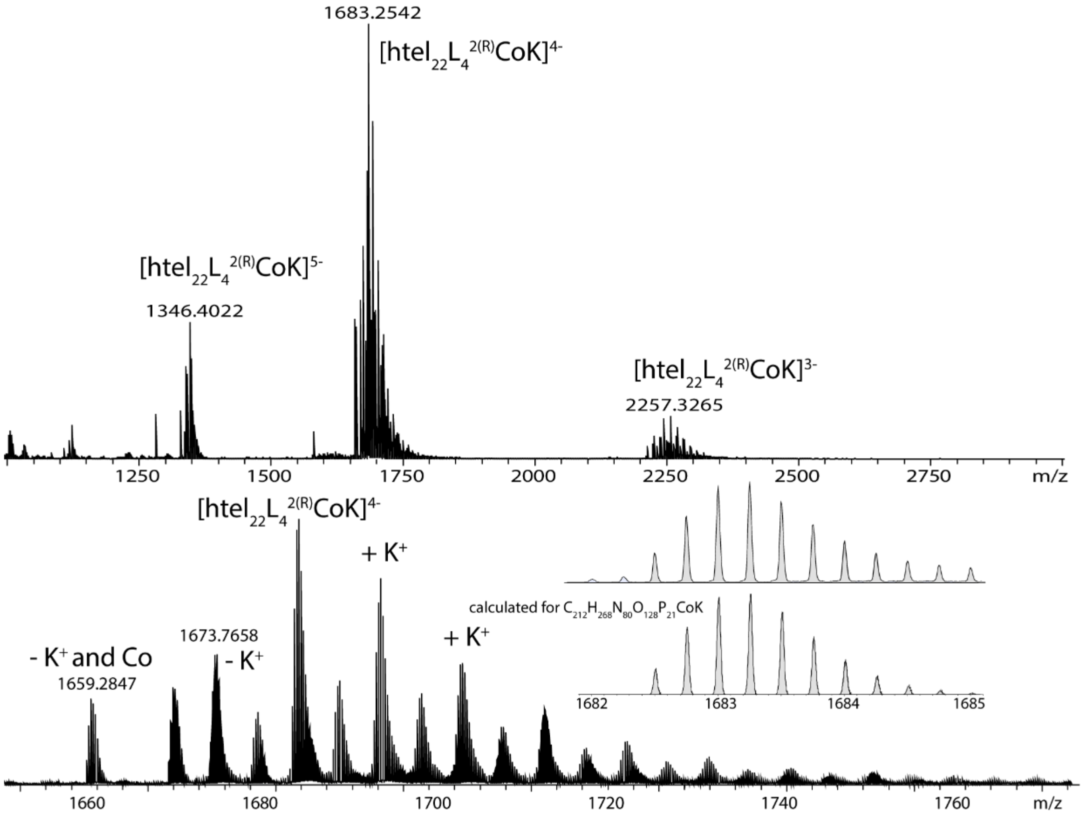

Figure 78. Native ESI-MS of htel$\mathbf{L}^{\mathbf{2}\boldsymbol{R}}{}_4$ with 1 equiv. Co(II).

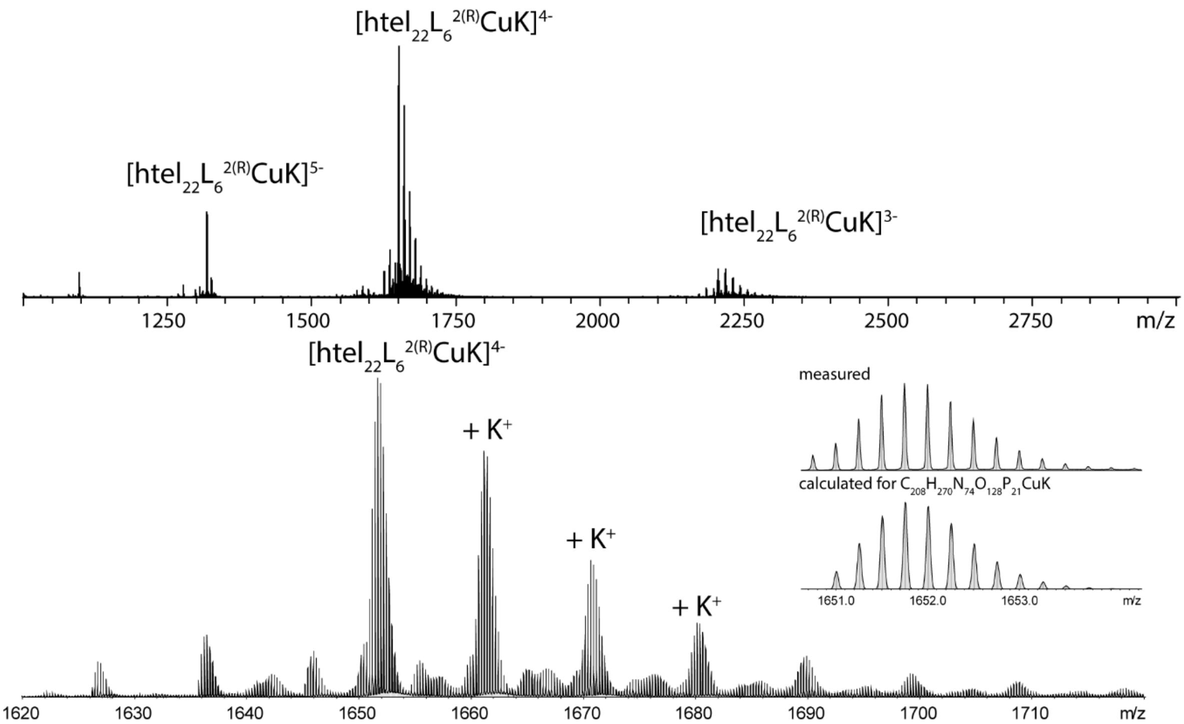

Figure 79. Native ESI-MS of htelL^{2R}_6 with 1 equiv. Cu(II).

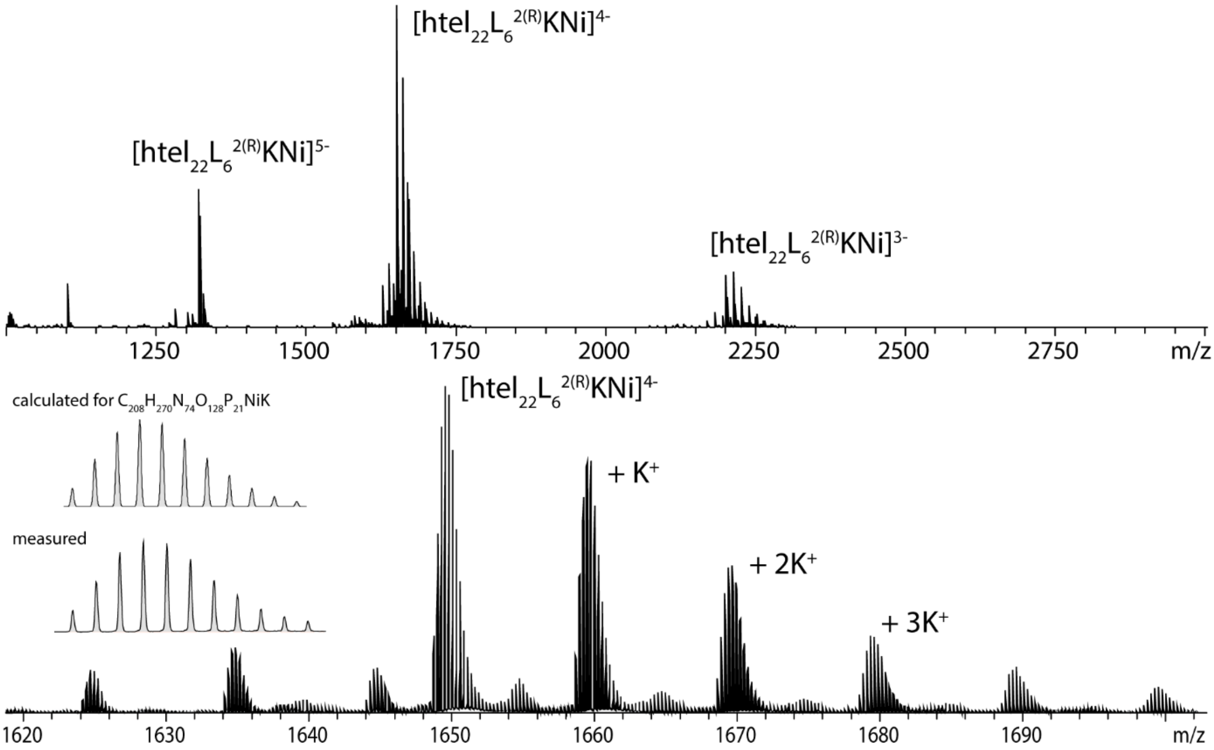

Figure 80. Native ESI-MS of htelL^{2R}_6 with 1 equiv. Ni(II).

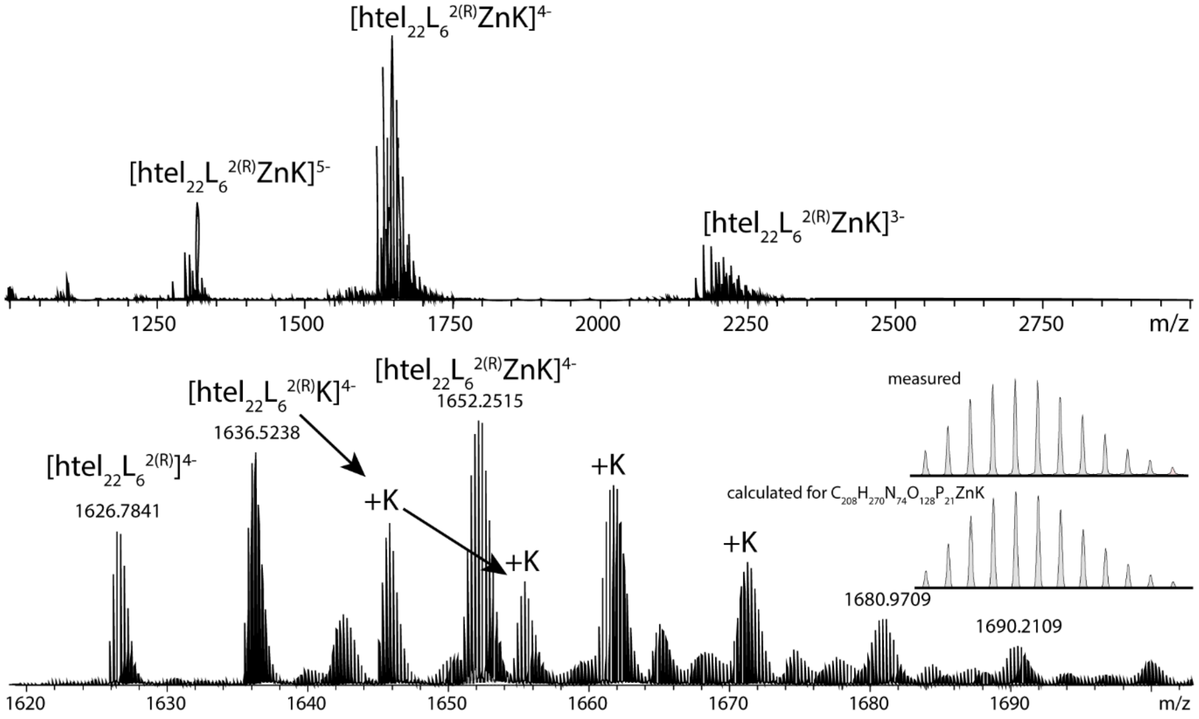

Figure 81. Native ESI-MS of htel$\mathbf{L}^{\mathbf{2}\boldsymbol{R}}_{6}$ with 1 equiv. Zn(II).

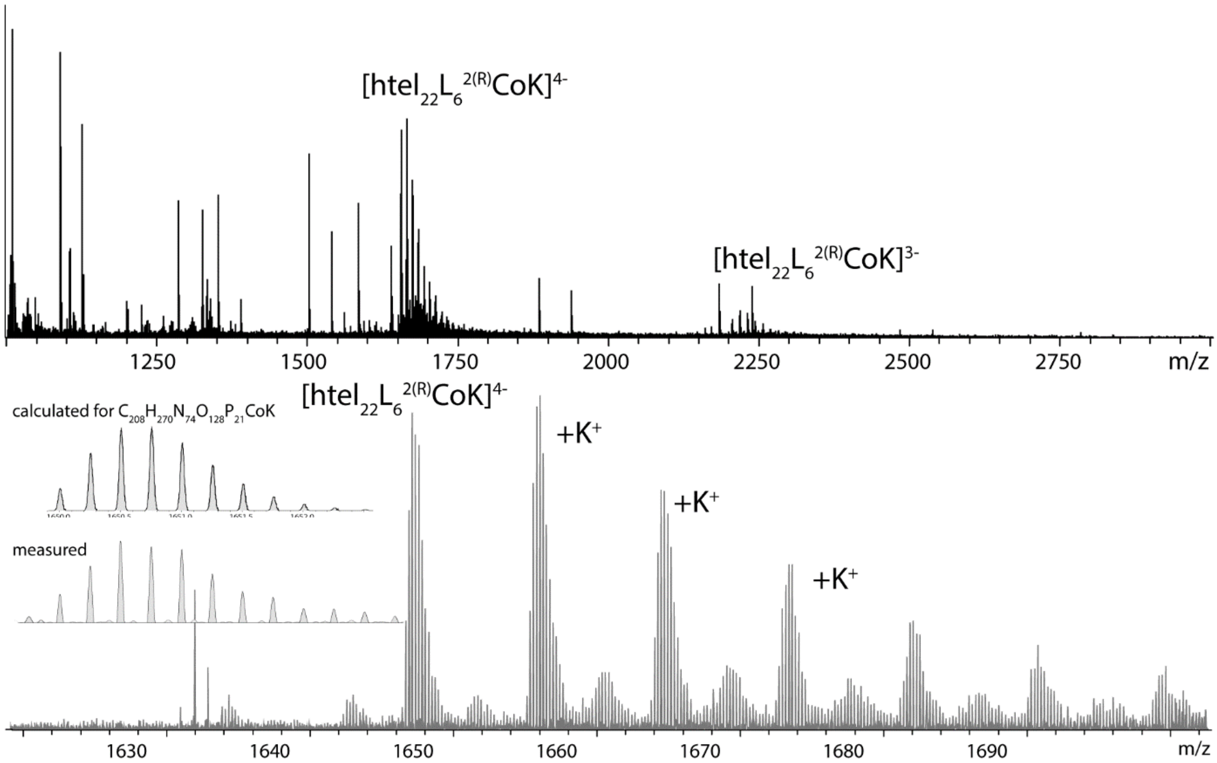

Figure 82. Native ESI-MS of htel$\mathbf{L}^{\mathbf{2}\boldsymbol{R}}_{6}$ with 1 equiv. Co(II).

7 MD simulations

Simulations were performed using GROMACS 2016.1 and GROMACS 2019.2.[3-9] The AMBER force field ff99bsc1 was modified with new force field parameters regarding the imidazole ligand according to a previously published protocol.[10-14] Missing forcefield parameters for the imidazole ligand were based on analogy to existing force field parameters. For both enantiomers, the same set of parameters was used. Parameters regarding the Cu(II) and Zn(II) complex were derived using VFFDT (Visual Force Field Derivation Toolkit) with a Gaussian '09 geometry optimized structure (B3LYP/6-311+G(d,p)).[10,15-18] To obtain a square-planar and tetrahedral coordination environment for Cu(II) and Zn(II), respectively, adjacent ligands were named differently, to avoid ambiguity in the parameterization. Improper dihedrals were used to maintain coplanarity between the imidazole plane and the imidazole – metal plane. The respective parameters for Cu(II) and Zn(II) were estimated based on literature values (Table 5).[17,18]

As starting structure, the crystal structure of a tetramolecular parallel stranded (pdb entry: 2O4F) and the NMR solution structure of an unimolecular antiparallel (143D, 1C35) G-quadruplex were used and modified.[19,20] Obtained starting structures where solvated in a rhombic bounding box with TIP3P water. Negative charges on the phosphates were neutralized with Na^+ (tetramolecular) or K^+ (unimolecular) cations and the concentration of Na/KCl was set to 100 mM. Prior to the MD simulation, the starting structure was subjected to three rounds of energy minimization and equilibrated at 298 K and 1 bar. The final MD was simulated for 100 ns in an NPT ensemble. For details see a previous publication.[10]

Partial charges (RESP)

Partial point charges were derived by RESP charge fitting. Therefore the imidazole ligands and the imidazole Cu(II) complex were fragmented according to the original capping scheme, structure optimized (B3LYP/6-311+G(d,p)) and submitted to the REDServer-Development (Table 4). Inter-molecular and intra-molecular charge constraints were used to maintain the correct total charge of the ligand. In all cases, the constrained charges were similar to the unconstrained values.[21-24]
For the Zn(II) complex a different procedure was applied. The complex was structure optimized (HF/6-31+G*) and used as input for antechamber to calculate the RESP charges.[25]

Table **4**. RESP charges for **L** in absence and presence of Cu(II) and Zn(II)

	Free ligand	Ligand in complex with Zn(II)	Ligand in complex with Cu(II)
O3'	-0.5155	-0.5155	-0.5155
C1	0.1600	0.1600	0.1600
H1	0.0978	0.0978	0.0978
C2	0.0465	0.0465	0.0465
H21	0.0578	0.0578	0.0578
H22	0.0578	0.0578	0.0578
O2	-0.5103	-0.5103	-0.5103
C3	-0.0023	-0.0023	-0.0023
H31	0.0851	0.0851	0.0851
H32	0.0851	0.0851	0.0851
O3	-0.3230	-0.361395	-0.3192
C4	0.0562	0.418549	0.1006
H41	0.0694	-0.033767	0.0693
H42	0.0694	-0.033767	0.0693
C5	-0.0772	-0.294730	-0.0844

H51	0.0773	0.143355	0.1129
H52	0.0773	0.143355	0.1129
N1	0.0761	0.221815	0.0283
C6	-0.2755	-0.287224	-0.1526
H6	0.1835	0.245354	0.2311
C7	0.0291	0.005499	-0.2316
H7	0.1429	0.134108	0.2126
NY	-0.5161	-0.418726	-0.0798
C8	0.0903	0.063523	-0.0253
H8	0.1463	0.135622	0.1800
P	1.2112	1.2112	1.2112
O4	-0.7996	-0.7996	-0.7948875
O5	-0.7996	-0.7996	-0.7948875
ZN		0.977716	
CU			0.3699

Table **5**: Forcefield parameters regarding the imidazole ligand L and the respective metal complexes with Cu and Zn

**Bond**				**bond length** b_0 **[nm]**	**force constant** k_b **[kJ mol⁻¹ nm⁻²]**	**comment**
CT	NB	1		0.1475	282001.6	Same as CT N*
CE	CI	1		0.1526	259408	Same as CE CT
CR	NX/Y	1		0.1335	408358.4	Same as CR NB
CV	NB	1		0.1394	343088	Same as CV NB
CV	CW	1		0.1375	428441.6	Same as CC CV
CW	NX/Y	1		0.1394	343088	Same as CV NB
NX/Y	CU	1		0.20219	69965.4	VFFDT
NX/Y	ZN	1		0.209	28325.7	VFFDT
**angle**				**angle**	**force constant** k_ϑ **[kJ mol⁻¹ nm⁻²]**	**comment**
OS	CI	CE	1	109.5	418.4	Same as OS CE CT
CE	CI	H1	1	109.5	418.4	Same as CE CT H1
CT	CI	CI	1	109.5	334.72	Same as CT CE CT
CI	CT	NB	1	109.5	418.4	Same as CT CT N*
CI	CE	OS	1	109.5	418.4	Same as OS CE CT
H1	CT	NB	1	109.5	418.4	Same as H1 CT N*
CW	CV	H4	1	120	418.4	Same as CC CV H4
CV	CW	H4	1	120	418.4	Same as CC CV H4
CR	NB	CT	1	128.8	585.76	Same as CK N* CT
CV	NB	CT	1	128.8	585.76	Same as CK N* CT
CW	CV	NB	1	120	585.76	Same as CW CC NB
CR	NX/Y	CW	1	120	585.76	Same as CR NA CW
NX/Y	CW	CV	1	120	585.76	Same as CC CW NA
NB	CR	NX/Y/Z	1	120	585.76	Same as NA CR NA
H4	CW	NX/Y/Z	1	120	418.4	Same as H4 CW NA
H5	CR	NX/Y/Z	1	120	418.4	Same as H5 CR NA

NX/Y	CU	NY/X	1		90	521.81	estimated
NX/Y	CU	NX/Y	1		180	512.5	estimated
CW	NX/Y	CU	1		126.8	890	VFFDT
CR	NX/Y	CU	1		126.9	857.2	VFFDT
CW	NX/Y	ZN	1		126.6	202.1	VFFDT
CR	NX/Y	ZN	1		126.6	199.6	VFFDT
NX/Y	ZN	NY/X	1		109.5	134.7	VFFDT
NX/Y	ZN	NX/Y	1		109.5	134.7	VFFDT
**improper dihedral**					**angle**	**force constant k_φ [kJ mol^{-1} nm^{-2}]**	**comment**
CW	CR	NX	CU	4	180	30	estimated
CW	CR	NX	ZN	4	180	30	estimated
**proper dihedral**					**anlge**	**force constant k_φ [kJ mol^{-1} nm^{-2}]**	**comment**
X	CV	CW	X	9	180	6.276	Same as X CC CW X
X	CT	NB	X	9	0	0	Same as X CT N* X
X	CW	NX/Y	X	9	180	9.7278	Same as X CW NA X
X	NX/Y	CR	X	9	180	9.7278	Same as X CW NA X
X	CE	CI	X	9	0	0.65084	same as X CT CT X
P	OS	CI	CE	9	297.765	4.518	same as P OS CE CT
CT	CE	CI	OS	9	348.09	4.0284	same as OS CI CT CT

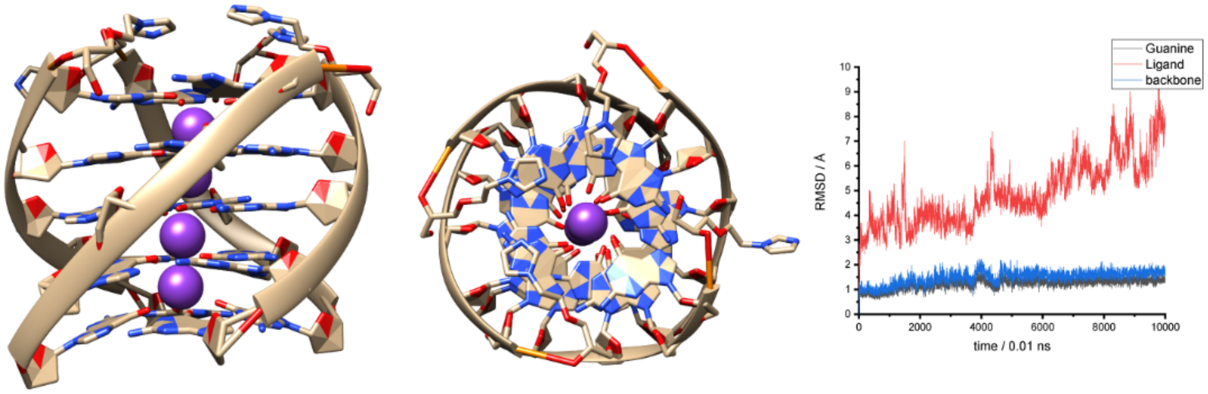

Figure 83. Representative MD-snapshot of G_5**L**^{2R}. Ligands were preferentially found to interact with the 5'-G-tetrad or the groove regions.

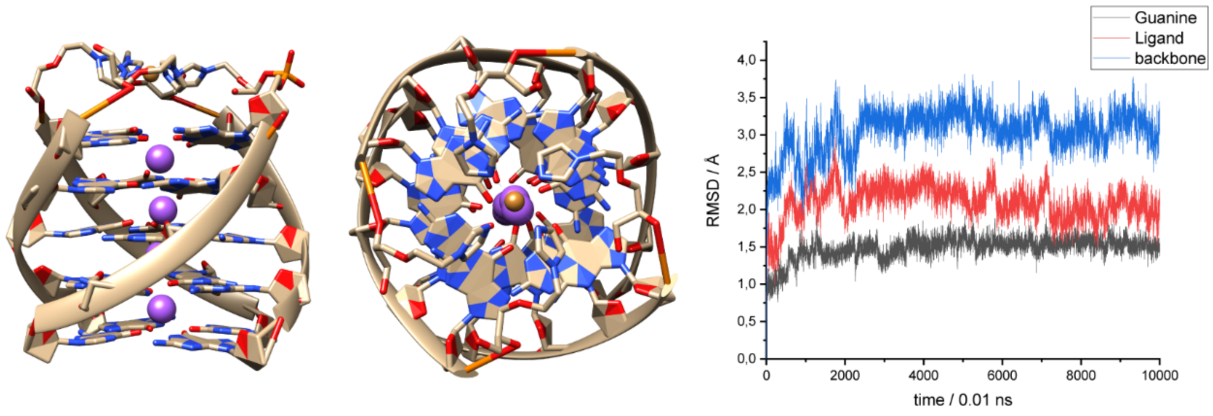

Figure 84. Representative MD-snapshot of G_5**L**^{2R} in complex with Cu(II). Coordination of **L** to Cu(II) led to a decreased RMSD compared to unbound **L** (Fig. 79).

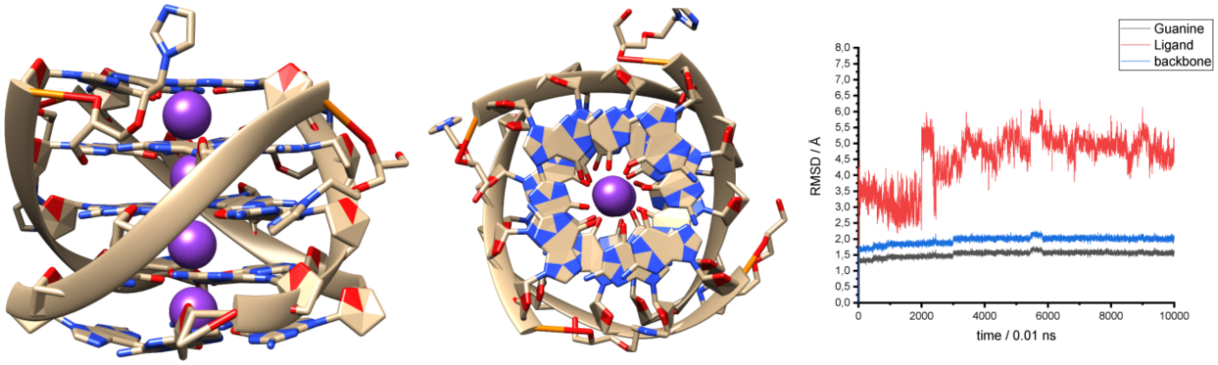

Figure 85. Representative MD-snapshot of G_5**L**^{2R} in the gas phase. **L** was mostly located in the groove regions of the G-quadruplex rather than interacting with the 5'-G-tetrad.

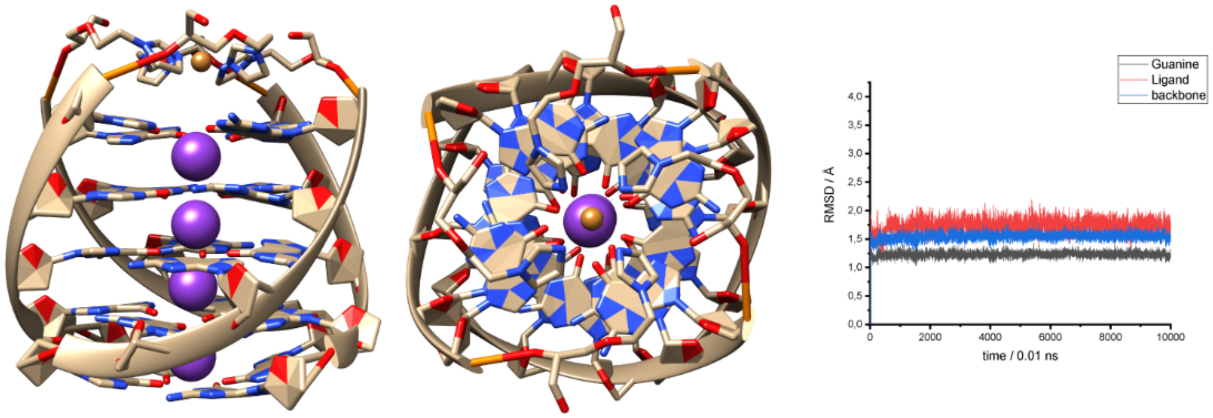

Figure 86. Representative MD-snapshot of G_5**L**^{2R} in complex with Cu(II) in the gas phase. **L** is coordinating Cu(II) in a propeller shaped square planar geometry and is not able to back fold into the groove regions (compare Fig. 81). This might cause the slight CCS increase after Cu(II) complexation.

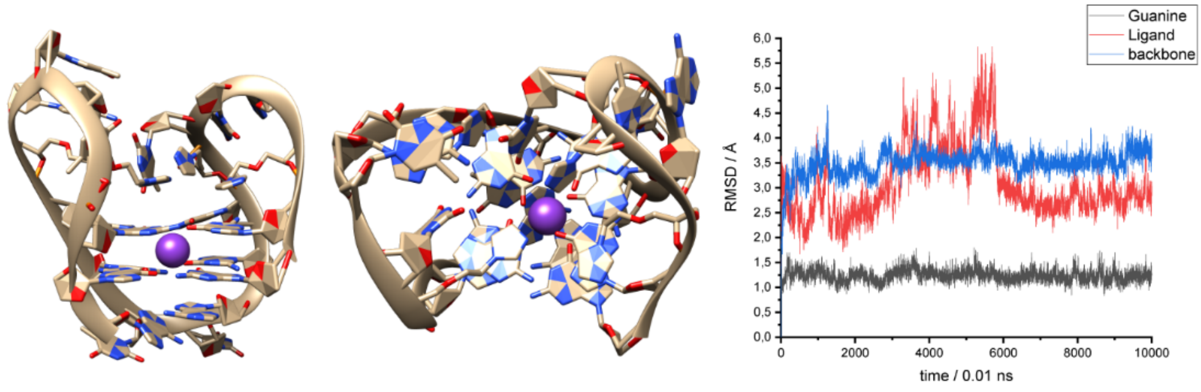

Figure 87. Representative MD-snapshot of htel**L**$^{2S}{}_{4}$. **L** was preferentially found to interact by π-π-stacking with the G-tetrad or nucleobases from the loop regions.

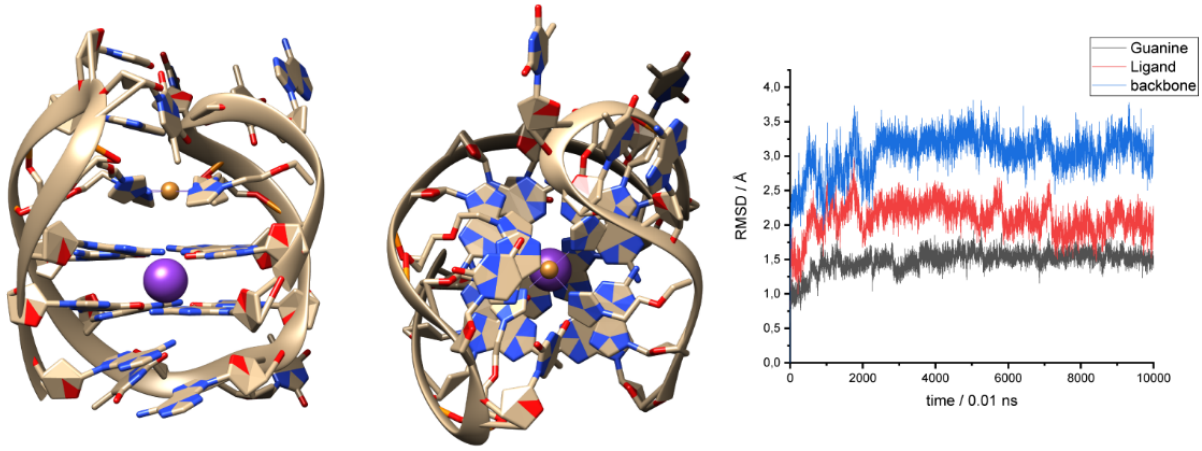

Figure 88. Representative MD-snapshot of htel**L**$^{2S}{}_{4}$ in complex with Cu(II). Complexation of Cu(II) leads to a decreased movement of **L** compared to the Cu(II) free sample as expressed by lower RMSD values.

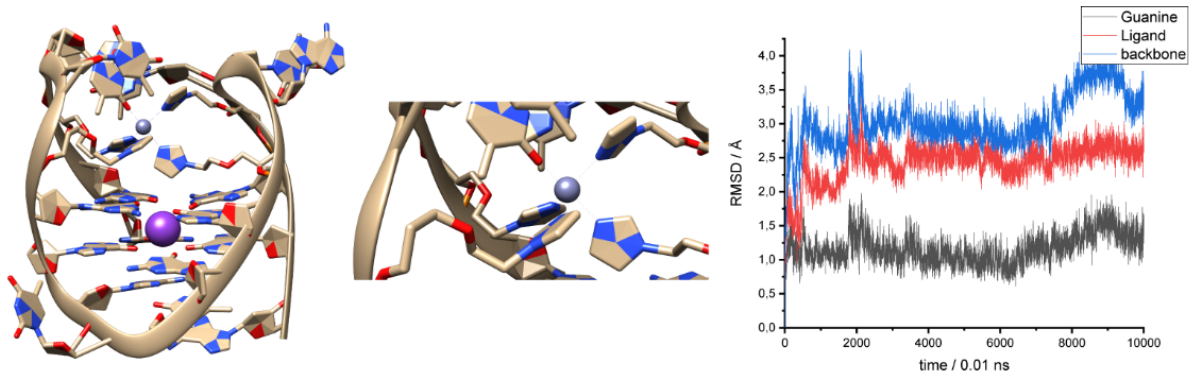

Figure 89. Representative MD-snapshot of htel**L**$^{2S}{}_{4}$ in complex with Zn(II). In contrast to Cu(II) for the Zn(II) complex five ligands were found coordinating to Zn(II) in a trigonal bipyramidal geometry. As additional ligand nucleobases from the loop regions as well as water were found. In this figure the carbonyl oxygen from thymine is coordinating to Zn(II).

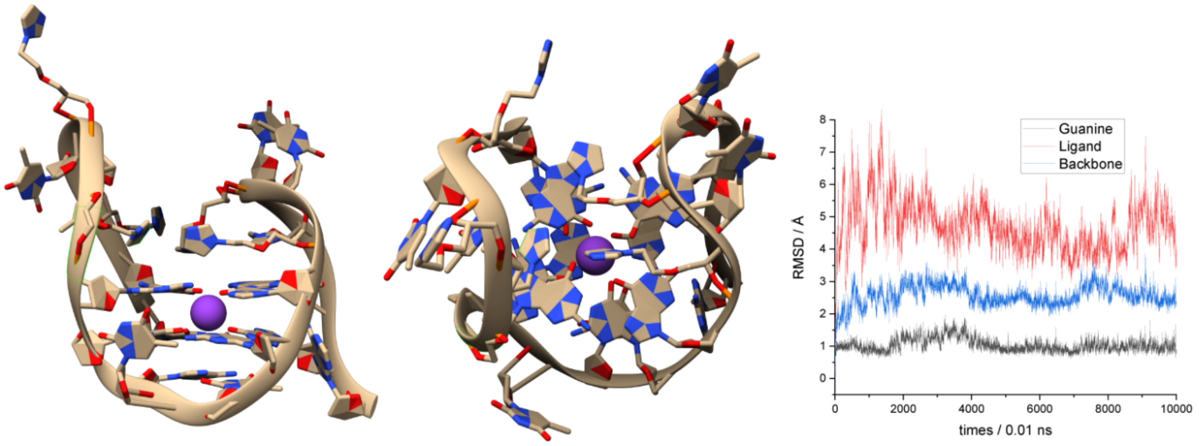

Figure 90. Representative MD-snapshot of htel**L**$^{2S}_{6}$. **L** was preferentially found to interact via π-π-stacking with the G-tetrad or nucleobases from the loop regions.

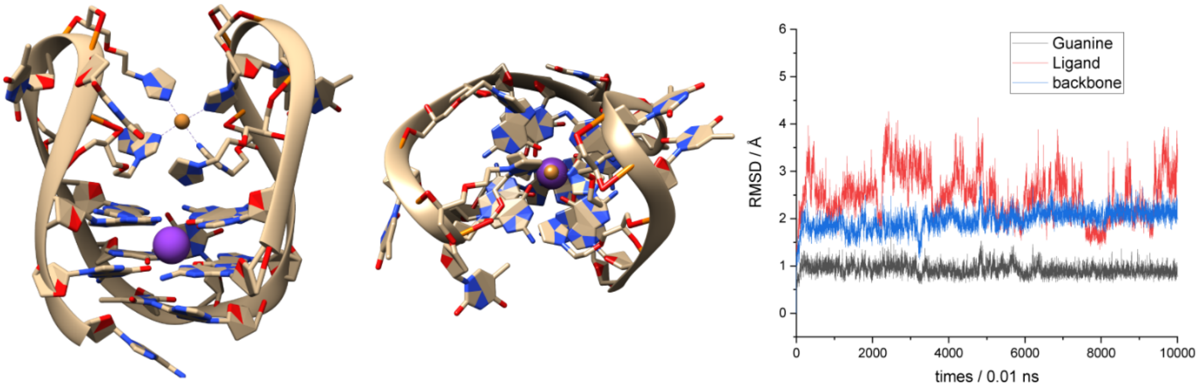

Figure 91. Representative MD-snapshot of htel**L**$^{2S}_{6}$ in complex with Cu(II). Complexation of Cu(II) leads to a decreased movement of **L** compared to the Cu(II) free sample as it is expressed in lower RMSD values.

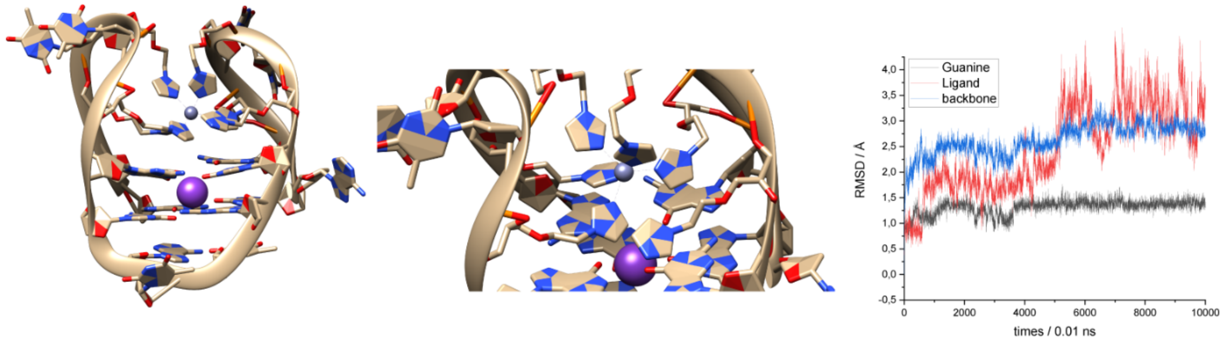

Figure 92. Representative MD-snapshot of htel**L**$^{2S}_{6}$ in complex with Zn(II). In contrast to Cu(II) for the Zn(II) complex five ligands were found coordinating to Zn(II) in a trigonal bipyramidal geometry. One of the two nonbonded imidazole ligands was usually involved in coordination.

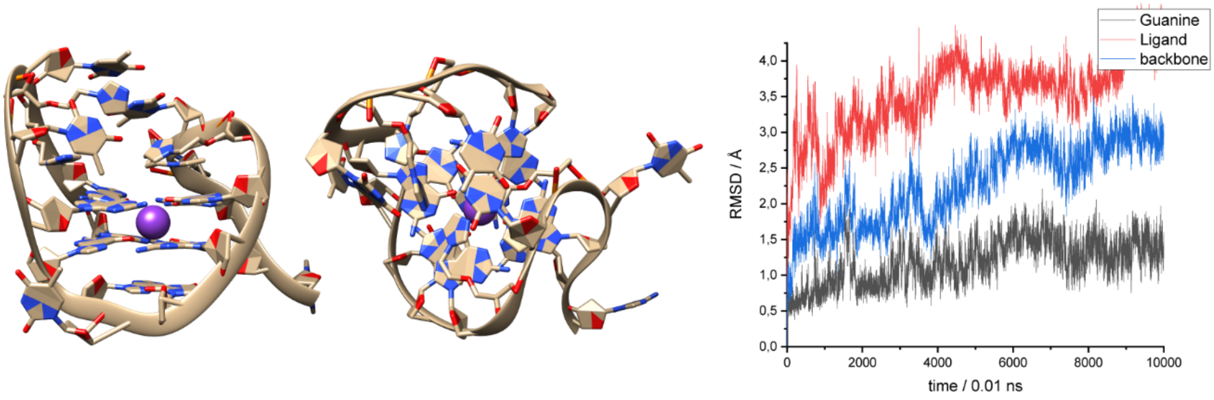

Figure 93. Representative MD-snapshot of htel**L**$^{2S}{}_{4}$C. **L** was preferentially found to interact via π-π-stacking with the G-tetrad or nucleobases from the loop regions.

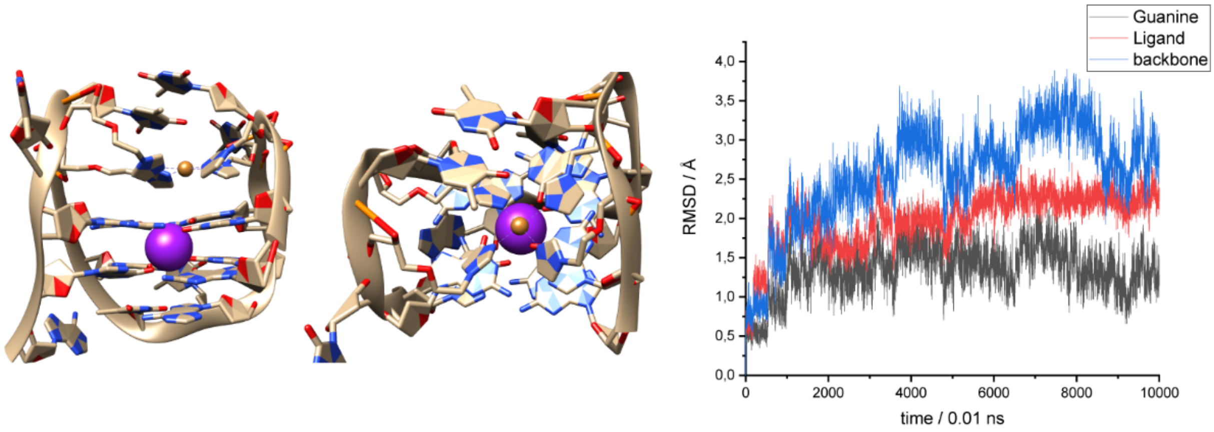

Figure 94. Representative MD-snapshot of htel**L**$^{2S}{}_{4}$C in complex with Cu(II). Complexation of Cu(II) leads to a decreased movement of **L** compared to the Cu(II) free sample as it is expressed in lower RMSD values.

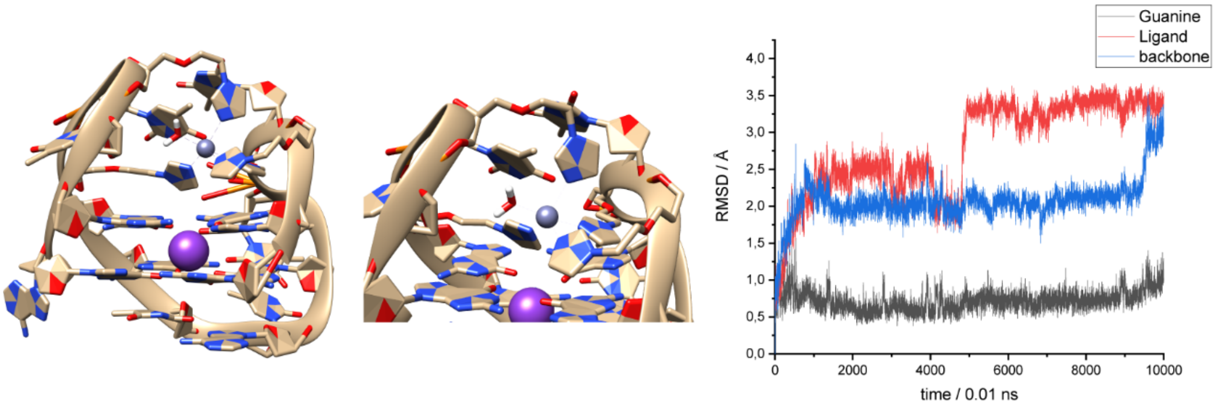

Figure 95. Representative MD-snapshot of htel**L**$^{2S}{}_{4}$C in complex with Zn(II). In contrast to Cu(II) for the Zn(II) complex five ligands were found coordinating to Zn(II) in a trigonal bipyramidal geometry. As additional ligand nucleobases from the loop regions as well as water were found. In this figure water is coordinating to Zn(II).

8 Oligonucleotide Analytics

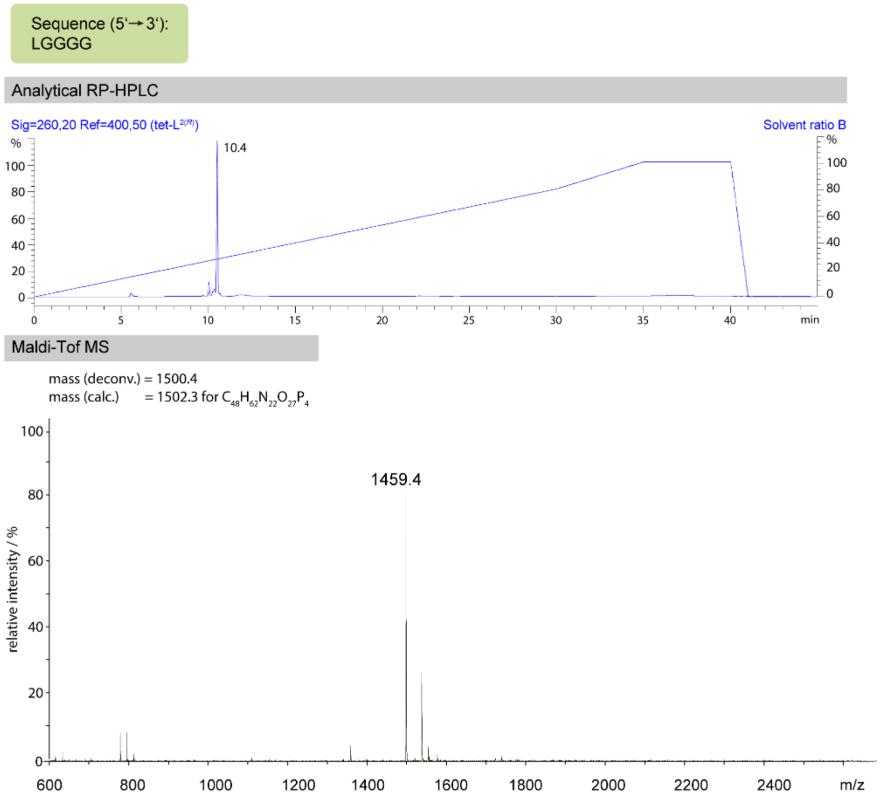

Figure 96. Analytical data for $G_4\mathbf{L}^{\mathbf{2}R}$

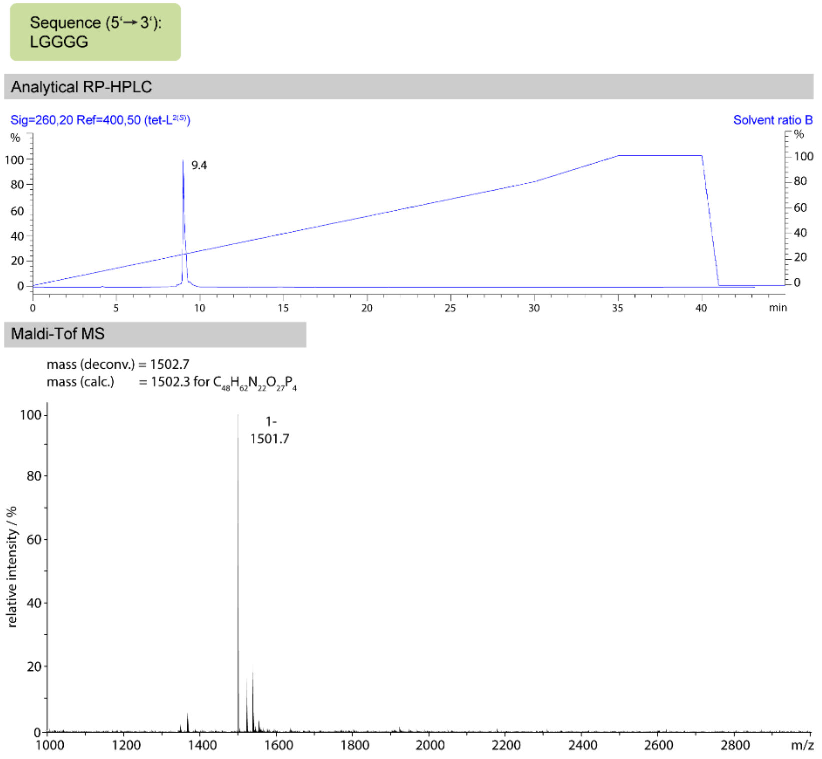

Figure 97. Analytical data for $G_4\mathbf{L}^{\mathbf{2}S}$

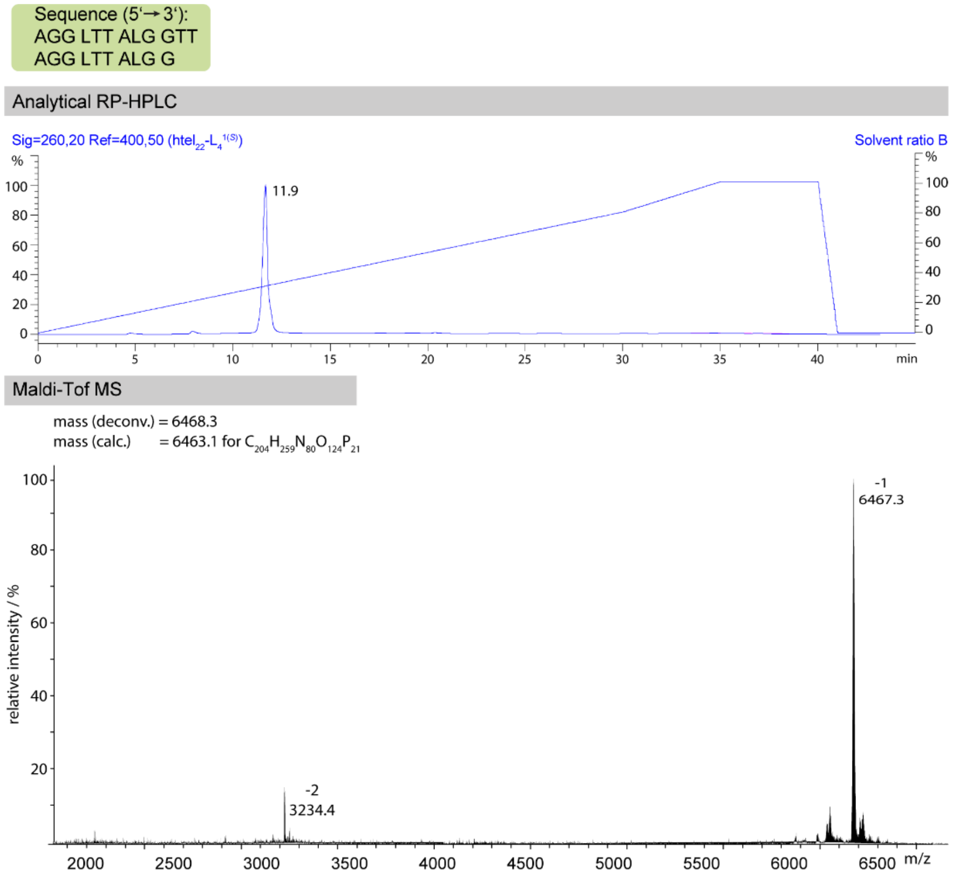

Figure 98. Analytical data for htel$\mathbf{L}^{1S}_4$

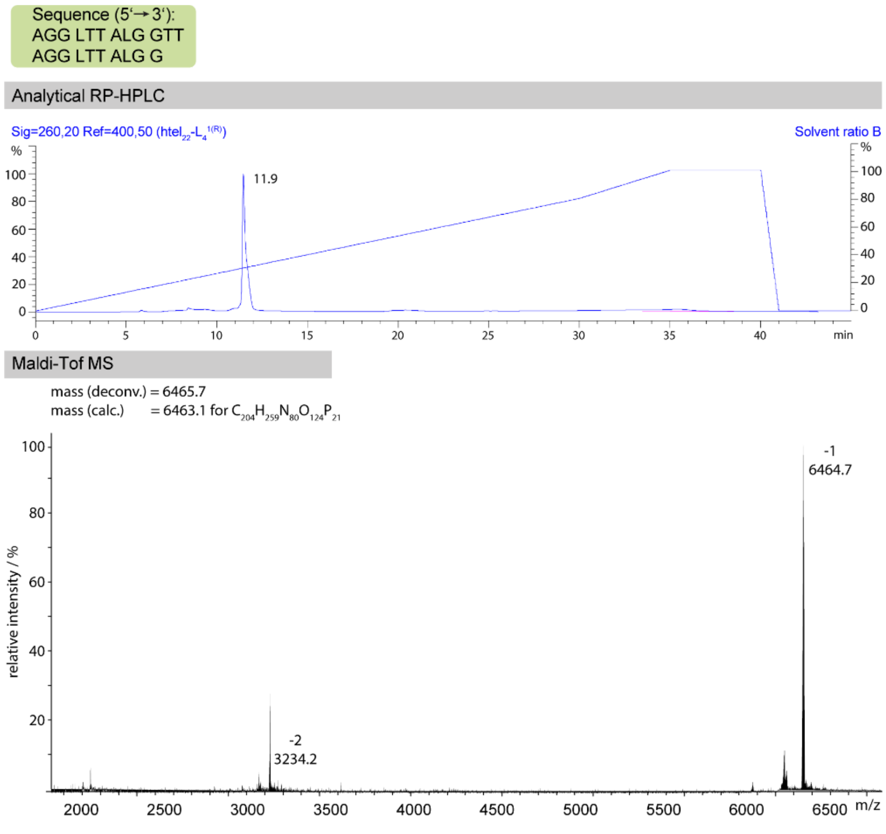

Figure 99. Analytical data for htel$\mathbf{L}^{1R}_4$

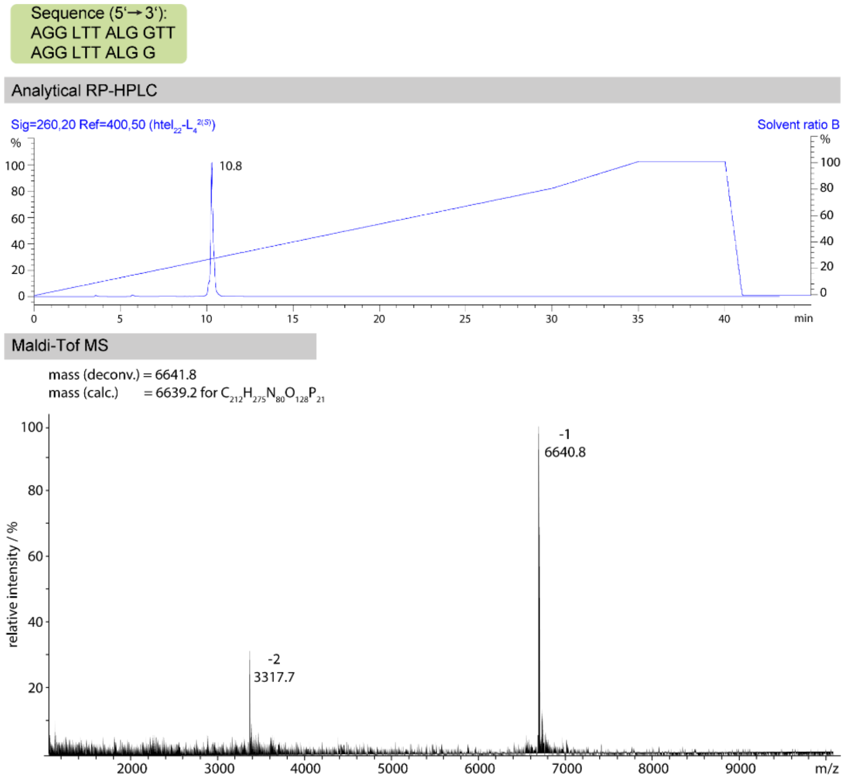

Figure 100. Analytical data for htel$\mathbf{L}^{2S}{}_4$

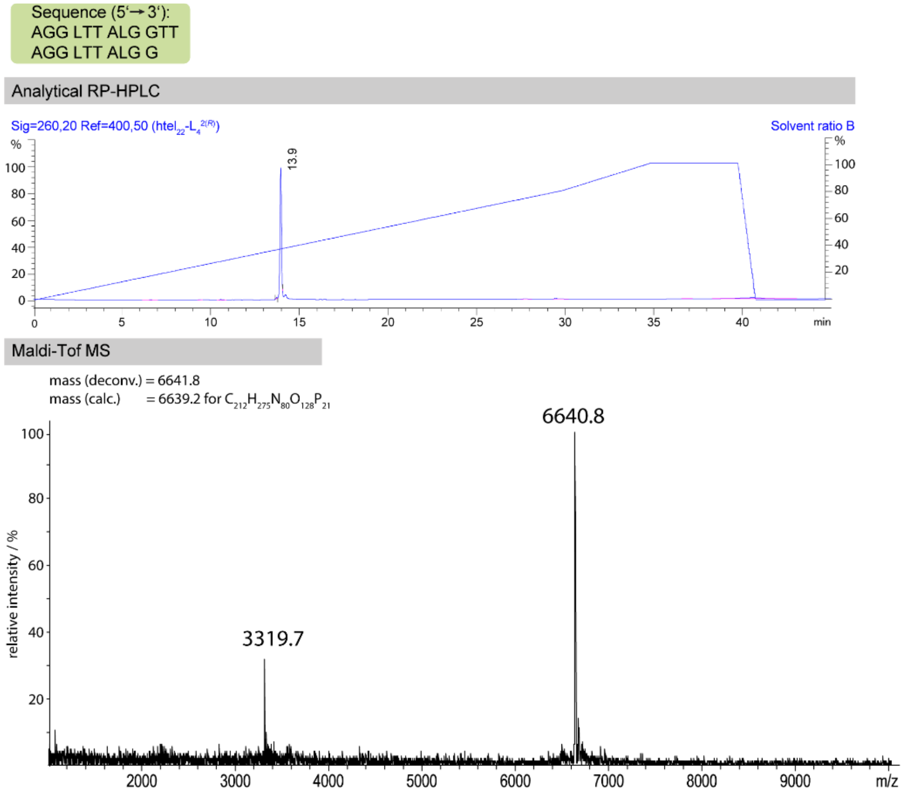

Figure 101. Analytical data for htel$\mathbf{L}^{2R}{}_4$

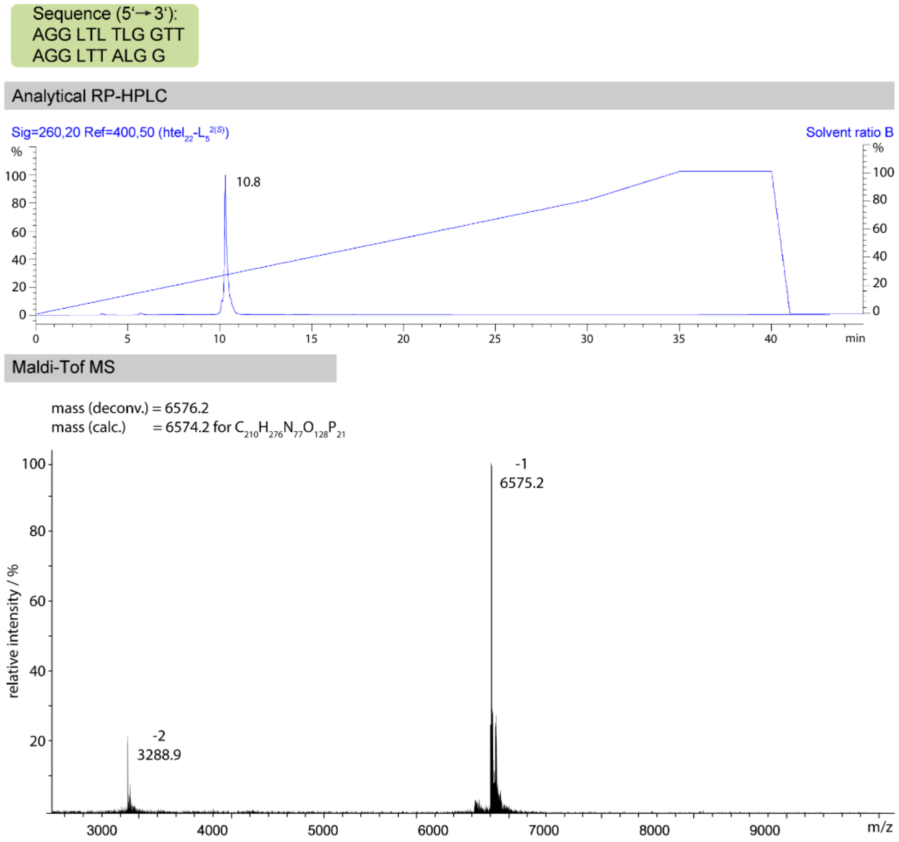

Figure 102. Analytical data for htel$\mathbf{L}^{2S}{}_5$

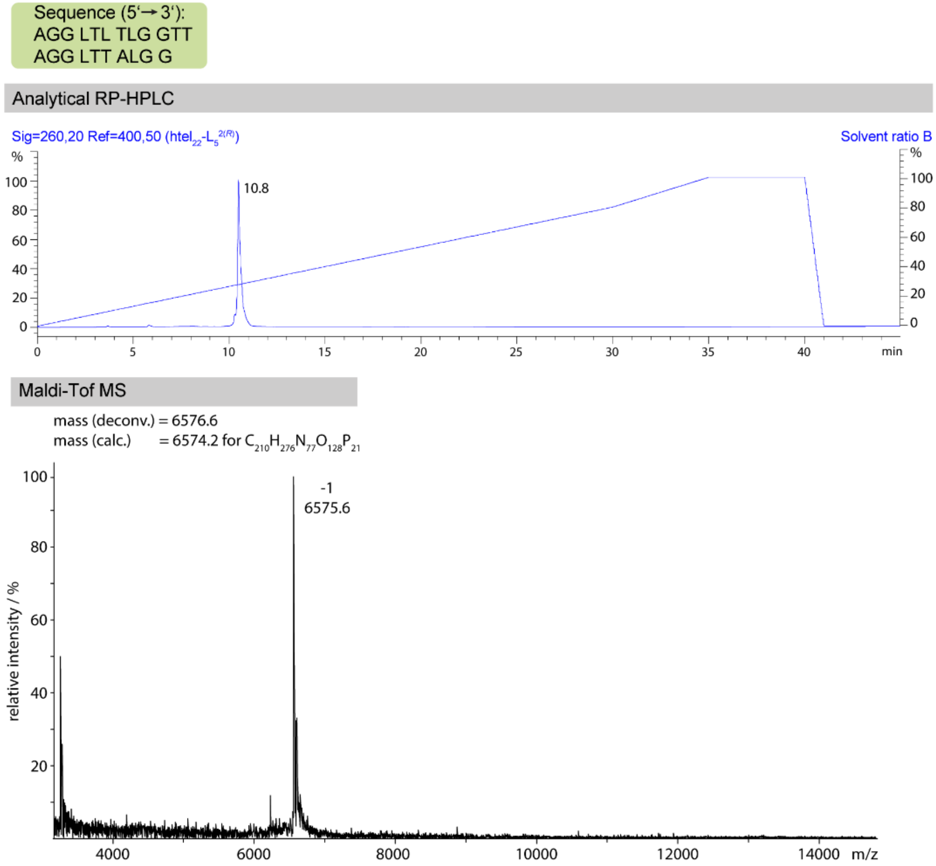

Figure 103. Analytical data for htel$\mathbf{L}^{2R}{}_5$

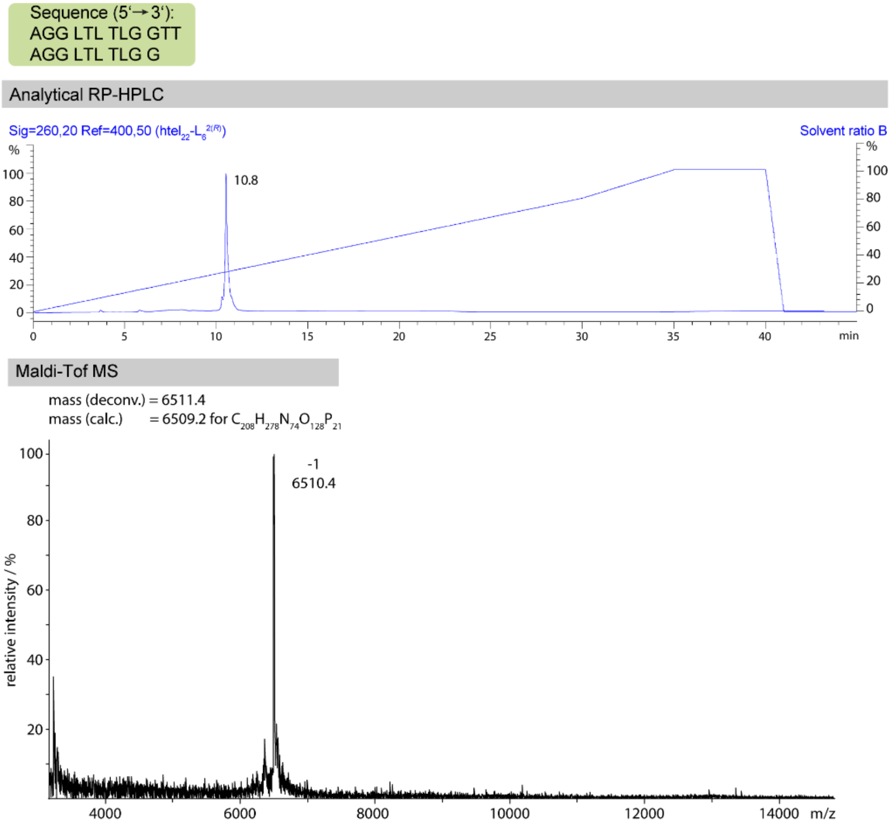

Figure 104. Analytical data for htel**L**^{2R}$_6$

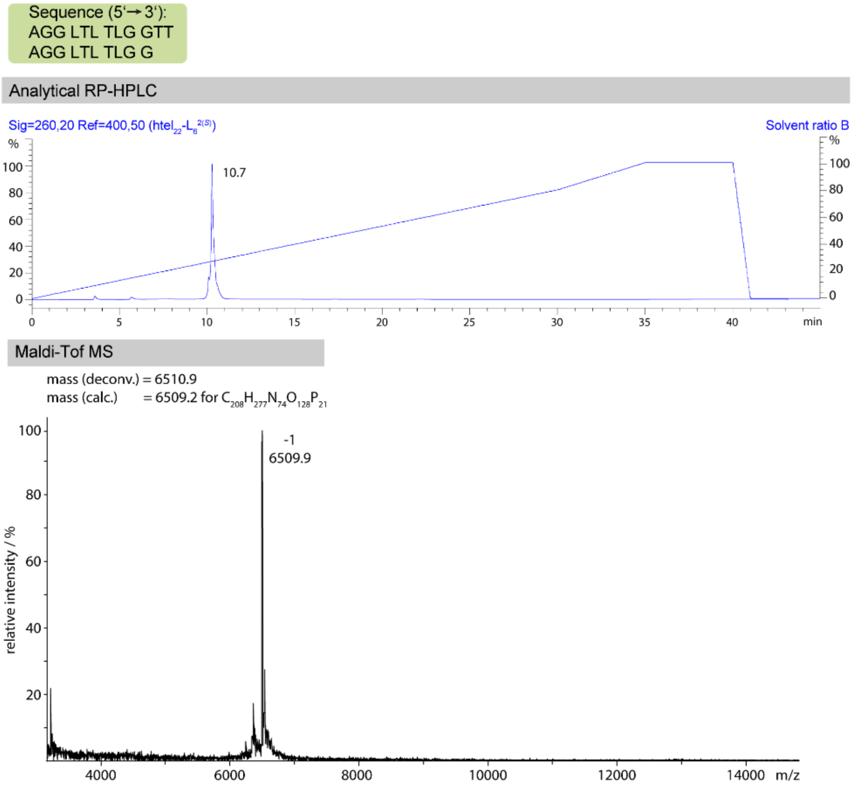

Figure 105. Analytical data for htel**L**^{2S}$_6$

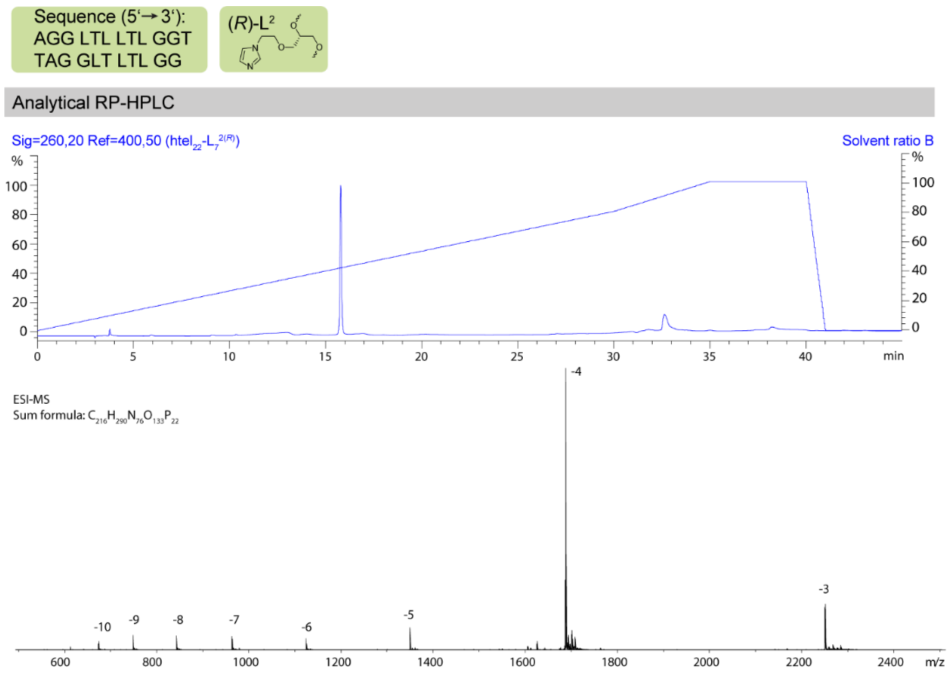

Figure 106. Analytical data for htel$\mathbf{L}^{2R}{}_{7}$

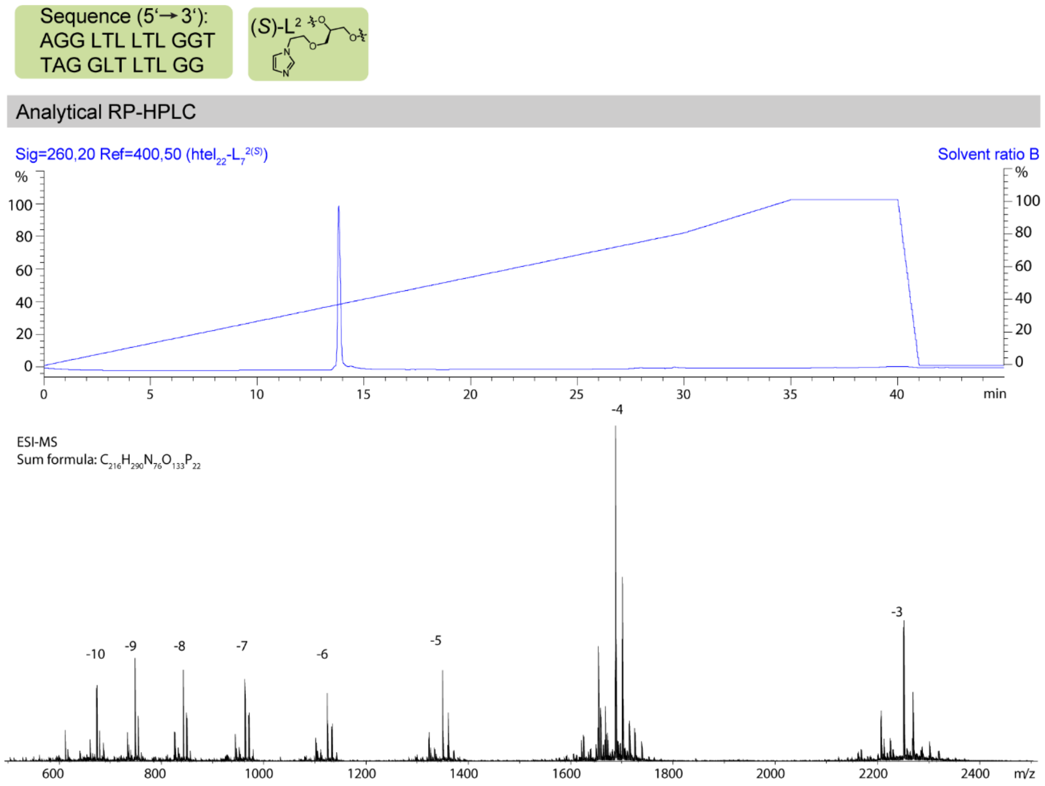

Figure 107. Analytical data for htel$\mathbf{L}^{2S}{}_{7}$

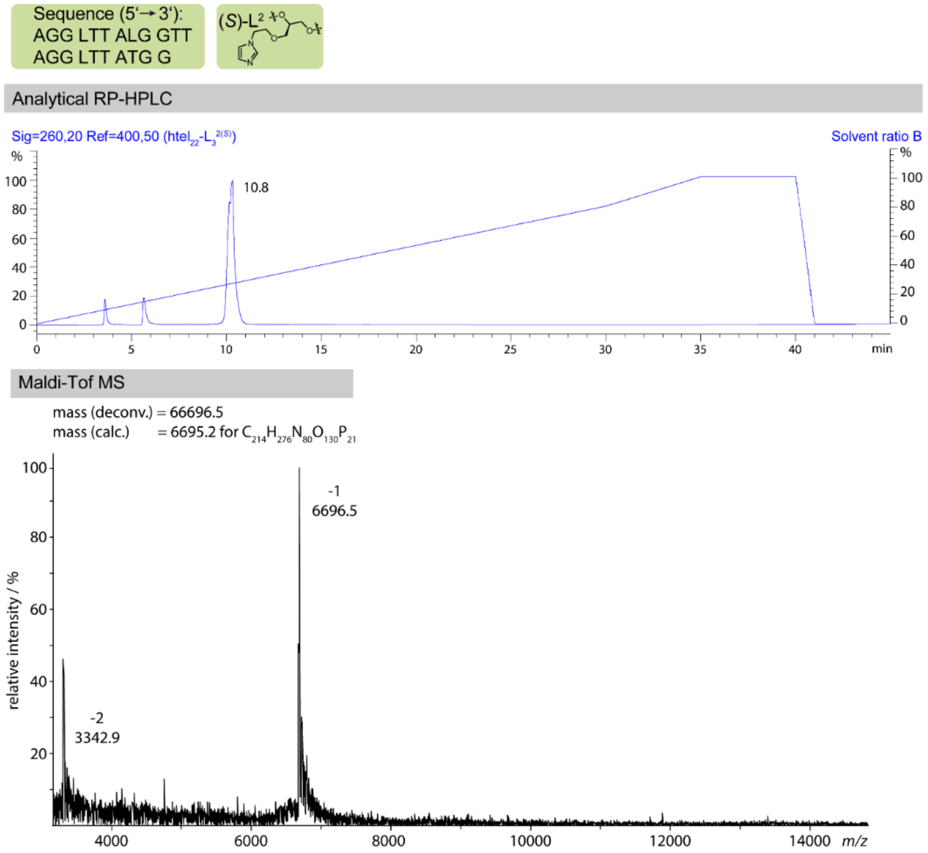

Figure 108. Analytical data for htel$\mathbf{L}^{2S}_{3}$

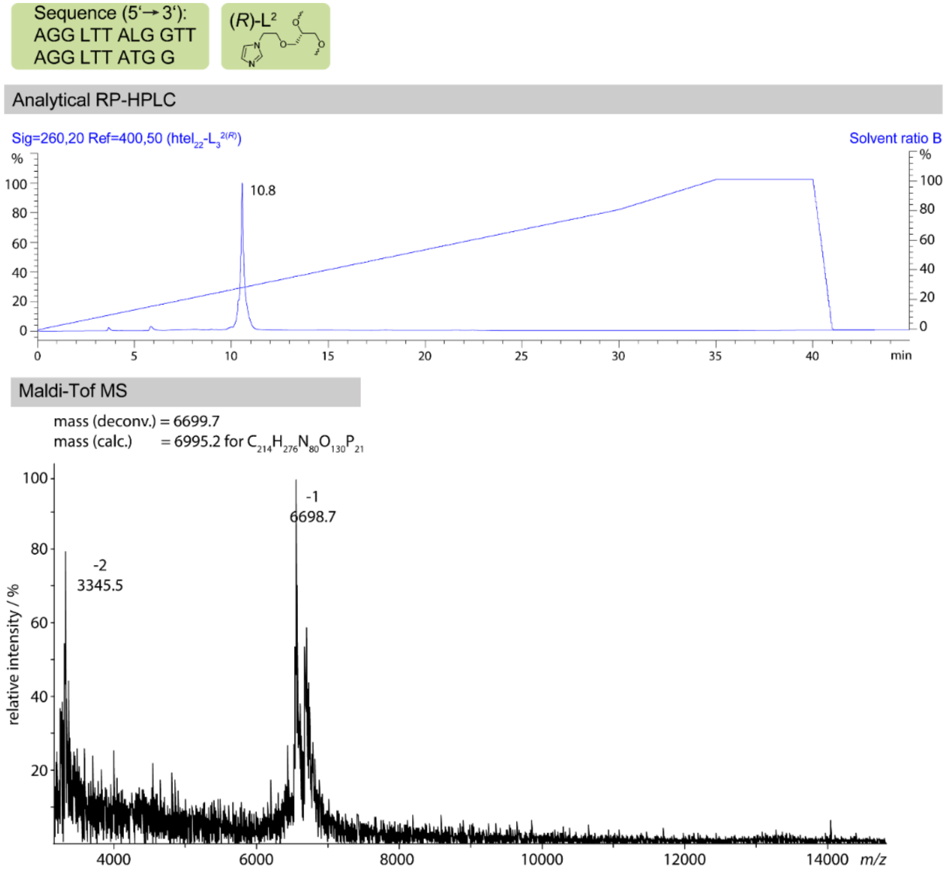

Figure 109. Analytical data for htel$\mathbf{L}^{2R}_{3}$

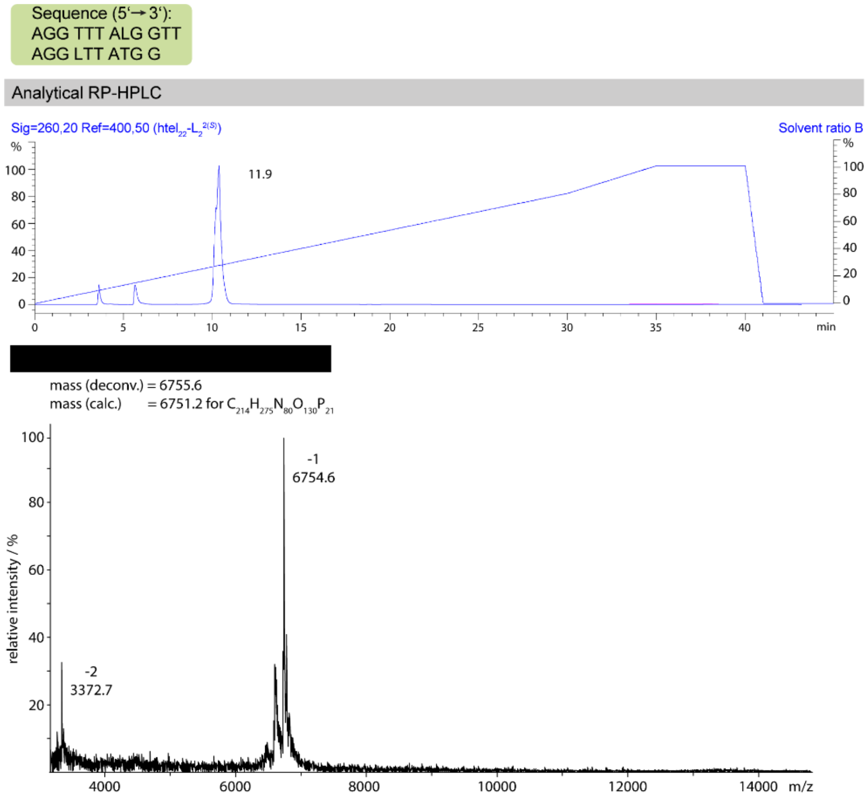

Figure 110. Analytical data for htel**L**$^{2S}_{2}$

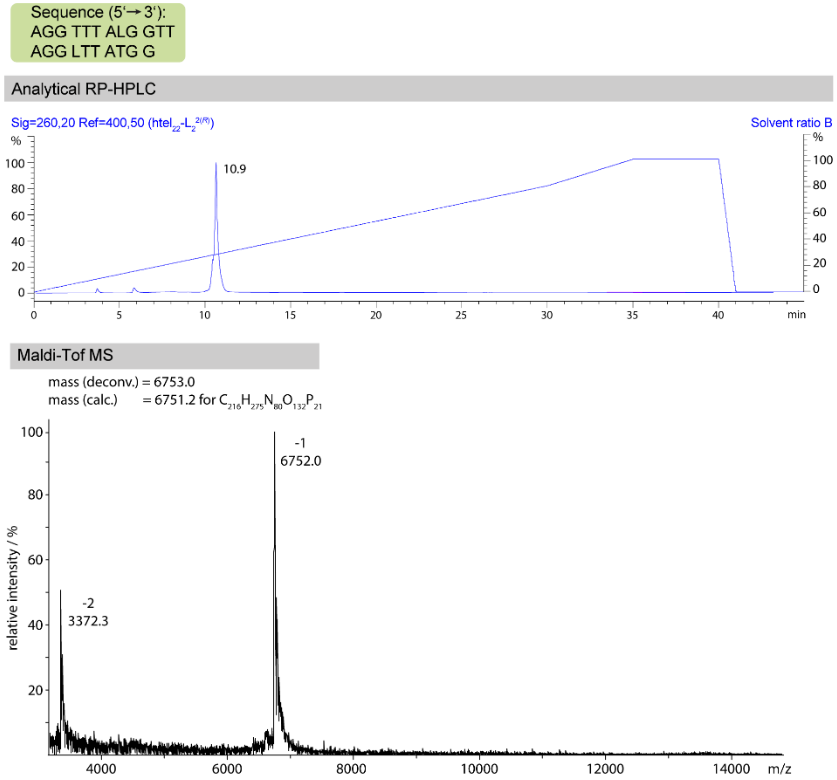

Figure 111. Analytical data for htel**L**$^{2R}_{2}$

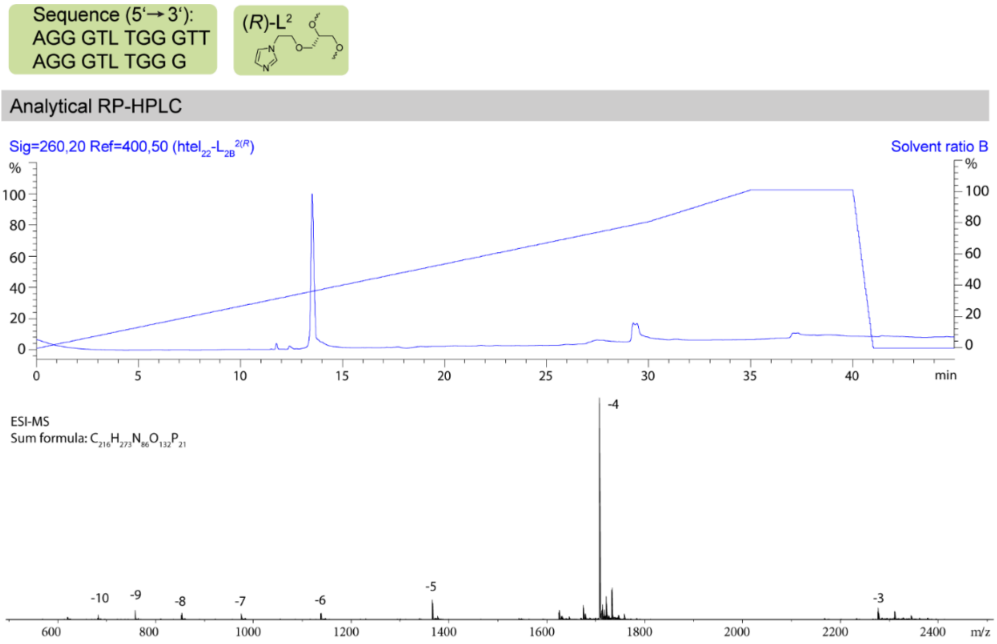

Figure 112. Analytical data for htel**L**$^{2R}_{2}$B

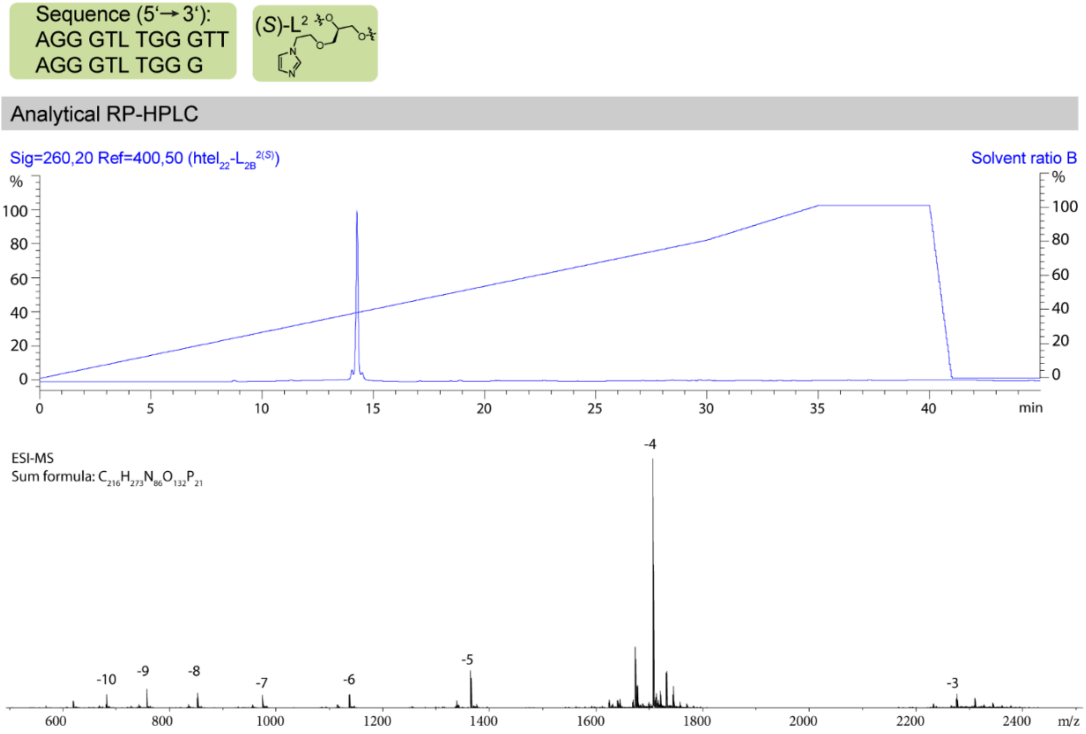

Figure 113. Analytical data for htel**L**$^{2S}_{2}$B

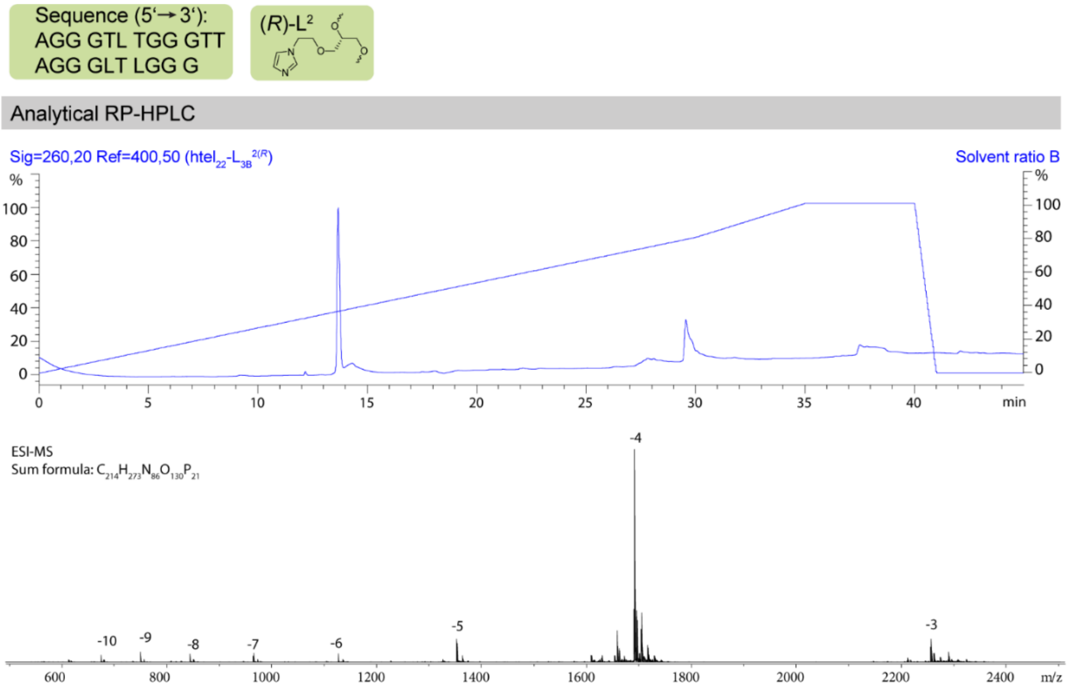

Figure 114. Analytical data for htel**L**$^{2R}_{3}$B

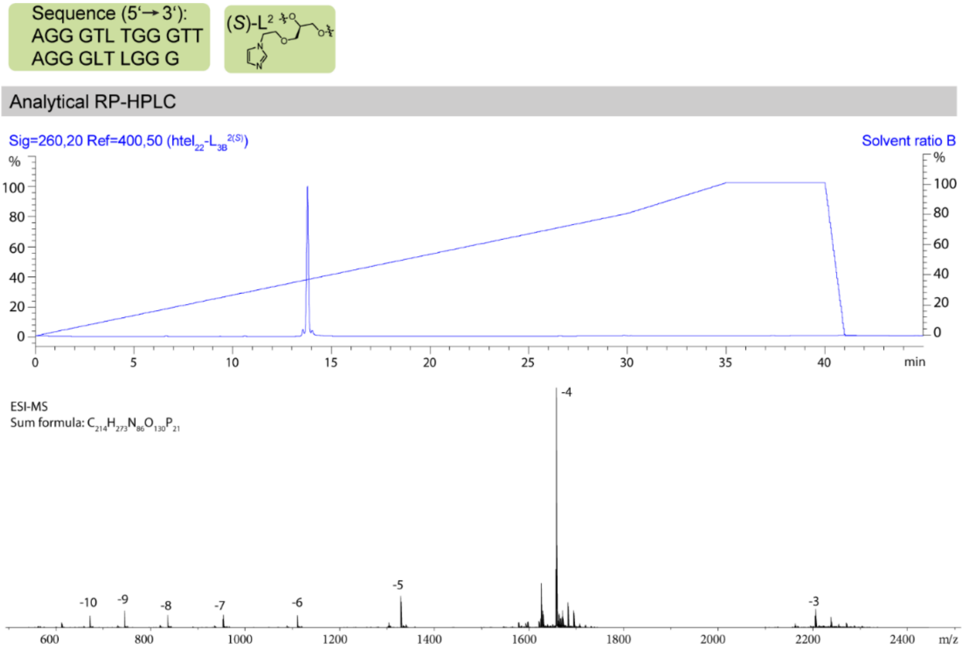

Figure 115. Analytical data for htel**L**$^{2S}_{3}$B

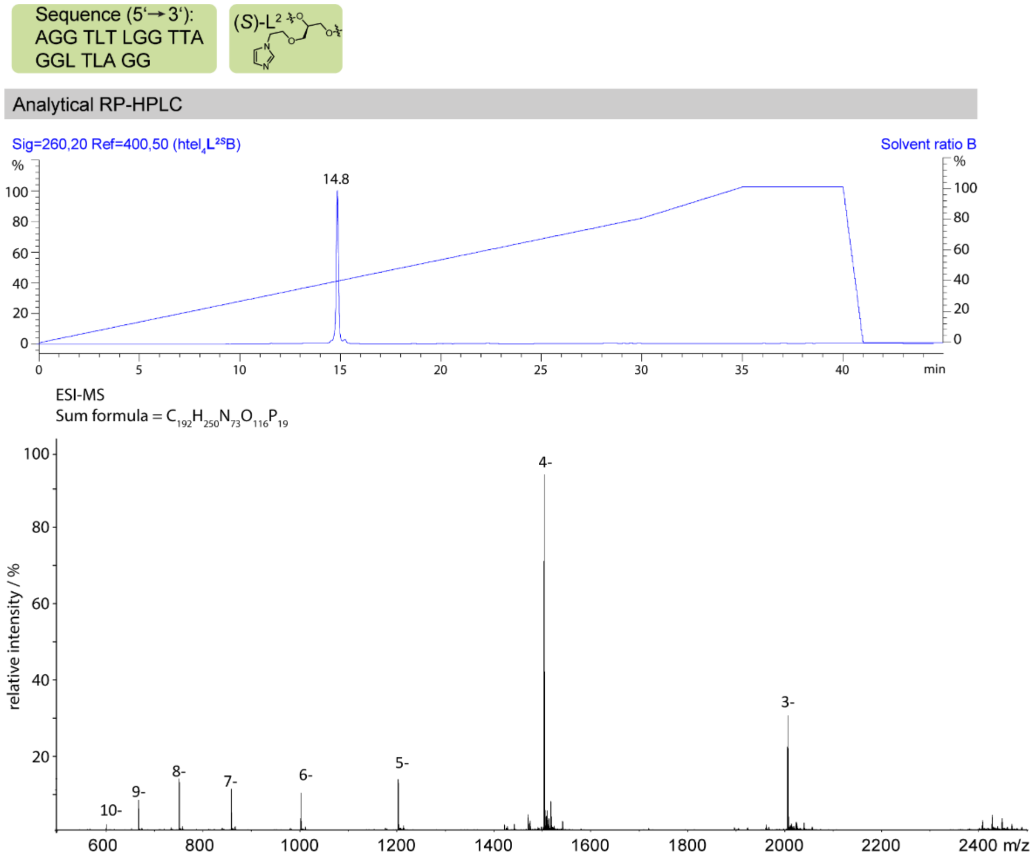

Figure 116. Analytical data for htel$\mathbf{L}^{2S}{}_4$B

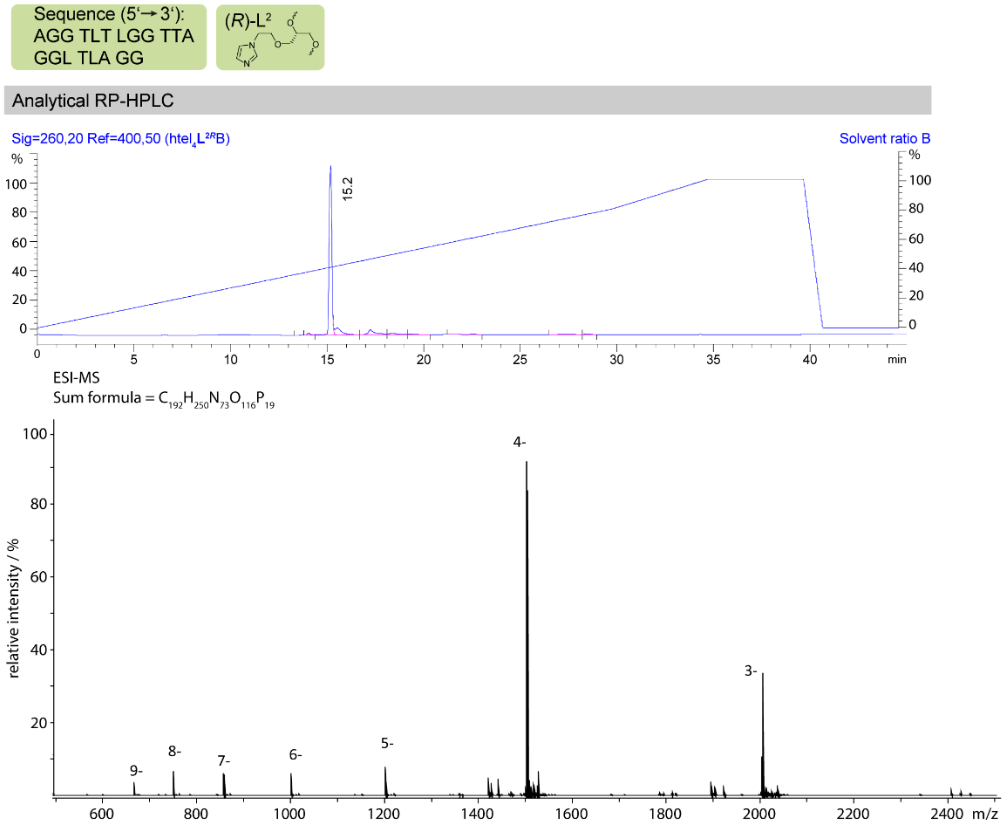

Figure 117. Analytical data for htel$\mathbf{L}^{2R}{}_4$B

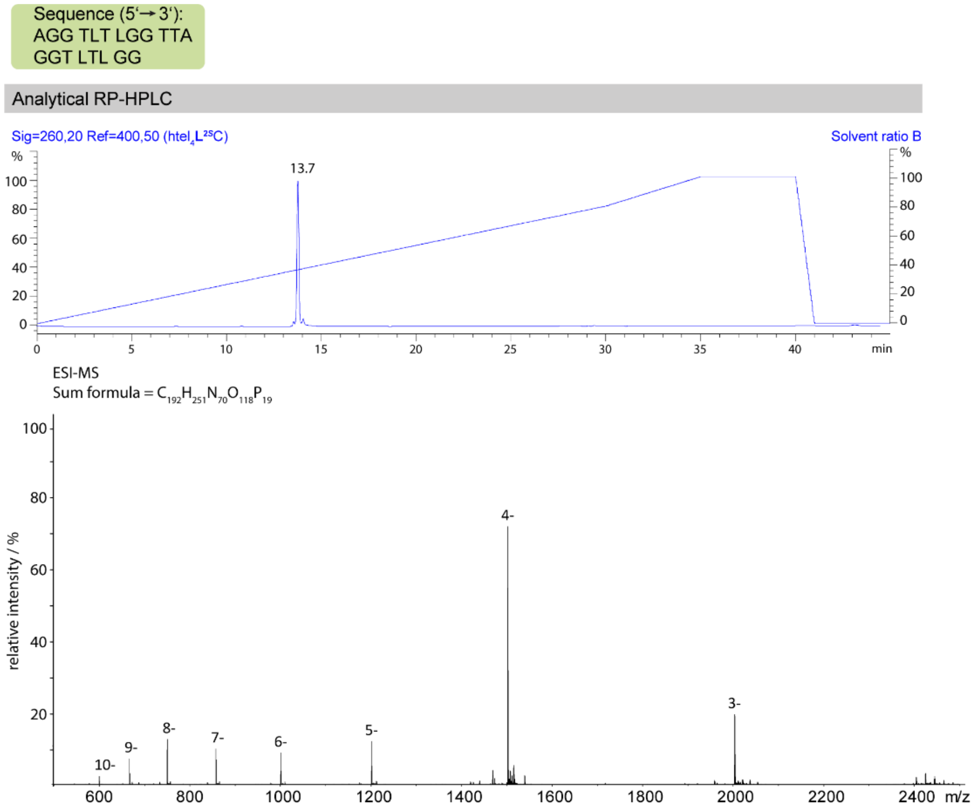

Figure 118. Analytical data for htel**L**^{2S}$_4$C

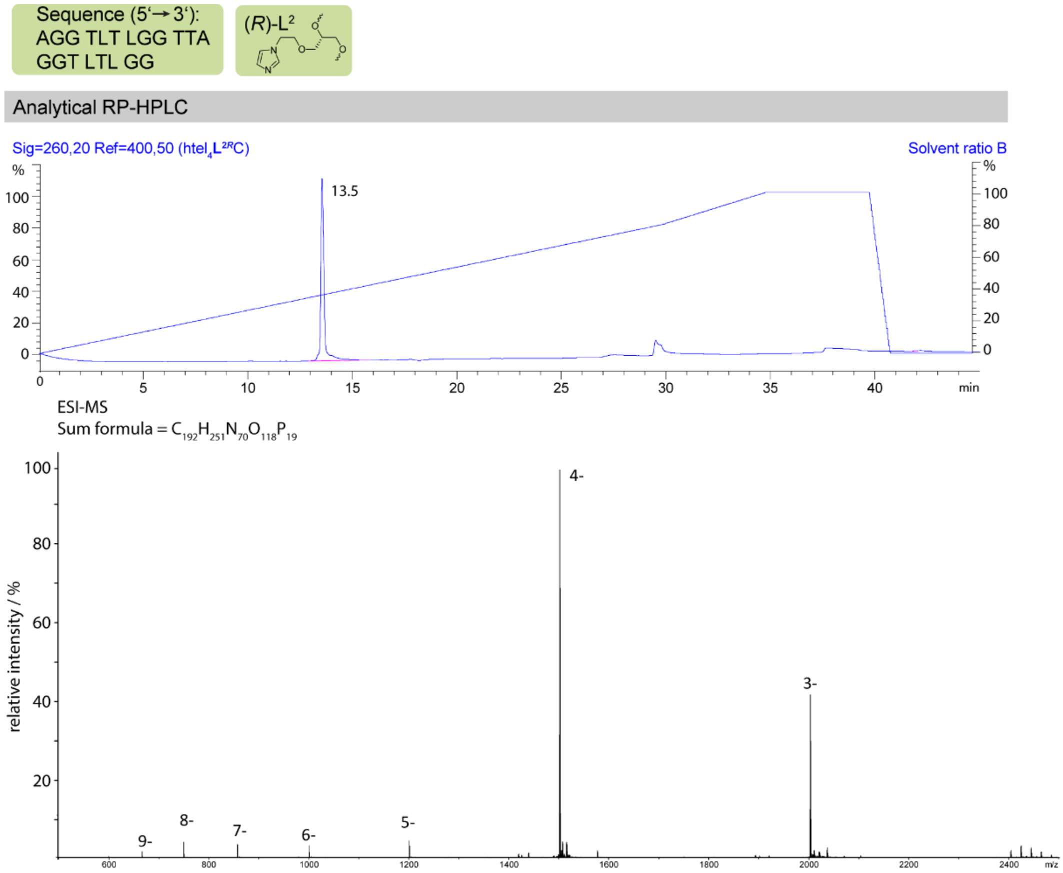

Figure 119. Analytical data for htel**L**^{2R}$_4$C

9 Literature

1 L. Zhang, A. E. Peritz, P. J. Carroll, E. Meggers, *Synthesis* **2006**, *4*, 645.
2 P. M. Punt, G. H. Clever, *Chem. Sci.* **2019**, *10*, 2513.
3 S. Pronk, S. Páll, R. Schulz, P. Larsson, P. Bjelkmar, R. Apostolov, M. R. Shirts, J. C. Smith, P. M. Kasson, D. van der Spoel, *Bioinformatics* **2013**, *29*, 845.
4 D. van der Spoel, E. Lindahl, B. Hess, G. Groenhof, A. E. Mark, H. J. C. Berendsen, *J. Comput. Chem.* **2005**, *26*, 1701.
5 B. Hess, H. Bekker, H. J. C. Berendsen, J. G. E. M. Fraaije, *J. Comput. Chem.* **1997**, *18*, 1463.
6 G. Bussi, D. Donadio, M. Parrinello, *J. Chem. Phys.* **2007**, *126*, 14101.
7 B. Hess, C. Kutzner, D. van der Spoel, E. Lindahl, *J. Chem. Theory Comput*. **2008**, *4*, 435.
8 M. J. Abraham, T. Murtola, R. Schulz, S. Páll, J. C. Smith, B. Hess, E. Lindahl, *SoftwareX* **2015**, *1-2*, 19.
9 S. Páll, M. J. Abraham, C. Kutzner, B. Hess, E. Lindahl in *Lecture Notes in Computer Science* (Eds.: S. Markidis, E. Laure), Springer International Publishing, Cham, **2015**.
10 D. M. Engelhard, J. Nowack and G. H. Clever, *Angew. Chem. Int. Ed*. **2017**, *56*, 11640.
11 D. M. Engelhard, L. M. Stratmann, G. H. Clever, *Chem. Eur. J.* **2018**, *24*, 2117.
12 D. M. Engelhard, A. Meyer, A. Berndhäuser, O. Schiemann and G. H. Clever, *Chem. Comm.* **2018**, *54*, 7455.
13 I. Ivani, P. D. Dans, A. Noy, A. Pérez, I. Faustino, A. Hospital, J. Walther, P. Andrio, R. Goñi, A. Balaceanu, G. Portella, F. Battistini, J. L. Gelpí, C. González, M. Vendruscolo, C. A. Laughton, S. A. Harris, D. A. Case, M. Orozco, *Nat. Methods* **2016**, *13*, 55.
14 A. Pérez, I. Marchán, D. Svozil, J. Šponer, T. E. Cheatham III, C. A. Laughton, M. Orozco, *Biophys. J.* **2007**, *92*, 3817.
15 M. J. Frisch, G. W. Trucks, H. B. Schlegel, G. E. Scuseria, M. A. Robb, J. R. Cheeseman, G. Scalmani, V. Barone, B. Mennucci, G. A. Petersson et al., *Gaussian 09, Revision D.01*, Gaussian, Inc., Wallingford CT, **2013**.
16 S. Zheng, Q. Tang, J. He, S. Du, S. Xu, C. Wang, Y. Xu, F. Lin, *J. Chem. Inf. Model.* **2016**, *56*, 811.
17 M. B. Peters, Y. Yang,B. Wang,L. Füsti-Molnár, M. N. Weave, K. M. Jr. Merz*, J. Chem. Theory. Comput.* **2010**, *6*, 2935.
18 R. Mera-Adasme, K. Sadeghian, D. Sundholm, C. Ochsenfeld, *J. Phys. Chem. B* **2014**, *118*, 13106.
19 C. Creze,B. Rinaldi,R. Haser, P. Bouvet,P. Gouet, *Acta Cryst. D* **2007**, *63*, 682.
20 a) Y. Wang, D. J. Patel, *Structure* **1993**, *1*, 263; b) V. M. Marathias, P. H. Bolton, *Nucleic Acids Res.* **2000**, *28*, 1969.
21 C. I. Bayly, P. Cieplak, W. Cornell, P. A. Kollman, *J. Phys. Chem.* **1993**, *97*, 10269.
22 F.-Y. Dupradeau, A. Pigache, T. Zaffran, C. Savineau, R. Lelong, N. Grivel, D. Lelong, W. Rosanski, P. Cieplak, *Phys. Chem. Chem. Phys.* **2010**, *12*, 7821.
23 E. Vanquelef, S. Simon, G. Marquant, E. Garcia, G. Klimerak, J. C. Delepine, P. Cieplak, F.-Y. Dupradeau, *Nucleic Acids Res.,* **2011**, *39*, 511.
24 F. Wang, J.-P. Becker, P. Cieplak, F.-Y. Dupradeau, R.E.D. Python: Object oriented programming for Amber force fields, Université de Picardie - Jules Verne, Sanford Burnham Prebys Medical Discovery Institute, **2013**.
25 J. Wang, W. Wang, P. A. Kollman, D. A. Case, *J Mol. Graph. Model.* **2006**, *25*, 247.
26 J.-L. Mergny, A. de Cian, A. Ghelab, B. Sacca, L. Lacroix, *Nucleic Acids Res.* **2005**, *33*, 81.
27 J. R. Wyatt, P. W. Davis, S. M. Freier, *Biochemistry* **1996**, *35*, 8002.
28 F. A. Fernandez-Lima, D. A. Kaplan, M. A. Park, *Rev. Sci. Instrum.* **2011**, *82*, 126106.
29 D. R. Hernandez, J. D. DeBord, M. E. Ridgeway, D. A. Kaplan, M. A. Park, F. Fernandez-Lima, *Analyst* **2014**, *139*, 1913.
30 J.-F. Greisch, J. Chmela, M. E. Harding, D. Wunderlich, B. Schäfer, M. Ruben, W. Klopper, D. Schooss, M. M. Kappes, *Phys. Chem. Chem. Phys.* **2017**, *19*, 6105.
31 Q. Zhai, M. Deng, L. Xu, X. Zhang, X. Zhou*, Bioorg. Med. Chem. Lett.* **2012**, *22*, 1142.
